# Photoredox Propargylation of Aldehydes Catalytic in Titanium

**DOI:** 10.1021/acs.joc.1c00521

**Published:** 2021-04-22

**Authors:** Francesco Calogero, Andrea Gualandi, Marco Di Matteo, Simone Potenti, Andrea Fermi, Giacomo Bergamini, Pier Giorgio Cozzi

**Affiliations:** † Alma Mater Studiorum, Università di Bologna, Dipartimento di Chimica “G. Ciamician”, Via Selmi 2, 40126 Bologna, Italy; ‡ Scuola Normale Superiore, Piazza dei Cavalieri 7, 56126 Pisa, Italy

## Abstract

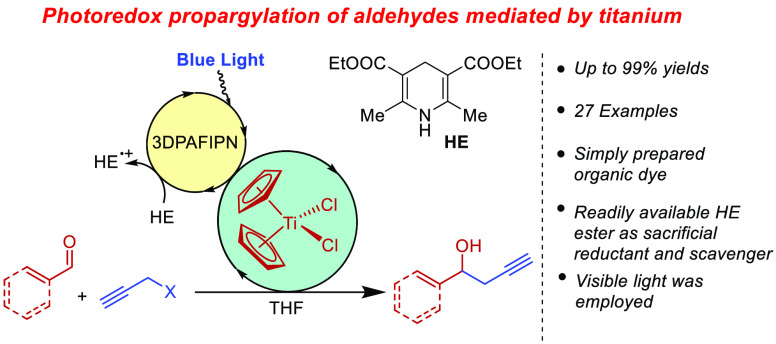

A practical and effective photoredox propargylation of aldehydes promoted by 10 mol % of [Cp_2_TiCl_2_] is presented. No stoichiometric metals or scavengers are used for the process. A catalytic amount of the cheap and simply prepared organic dye 3DPAFIPN is used as the reductant for titanium. The reaction displayed a broad scope, and no traces of allenyl isomers were detected for simple propargyl bromide, whereas mixtures of propargyl and allenyl isomers were observed for substituted propargyl bromides.

In just a few years, photoredox catalysis has reached an extraordinary level of advancement, introducing new and exciting methodologies in organic chemistry.^1^ Besides electron transfer, many interesting reactions can also be promoted by photocatalytic methodologies using energy transfer (EnT) to reach a reactive transition state in molecules or complexes.^2^ Now, dual photoredox catalysis,^3^ that is the combination of metal-promoted processes with photoredox cycles, is in continuous development.^4^ From the application of synergistic dual photoredox catalysis to cross-coupling reactions, metal-catalyzed processes were addressed to radical to polar cross-over reactions (RPC),^5^ with the aim of developing important C–C bond-forming reactions.^6^ In this context, allylation methodologies were introduced with chromium,^7^ nickel,^8^ and titanium.^9^ Particularly, the earth-abundant titanium can give important advantages in terms of sustainability and eco-friendliness of the process,^10^ and its use in combination with photoredox catalysis was first explored by Gansäuer.^11^ In addition, the interesting photophysical properties of titanium complexes make the further exploration of their chemistry in the excited state even more intriguing.^12^ We recently have reported the allylation reaction of aldehydes, employing 10 mol % of the inexpensive [Cp_2_TiCl_2_], in the presence of an organic dye, 3DPAFIPN,^13^ and Hantzsch’s ester as the stoichiometric reductant.^9^ Quite recently, Glorius has reported an interesting carbonyl propargylation^14^ via dual chromium/photoredox catalysis, taking advantage of a catalytic radical three components coupling of 1,3-enynes, aldehydes, and radical precursors, in the presence of CrCl_3_ and visible light.^15^ However, the direct use of propargylic halides or alkynes for the generation of propargyl chromium species is still not reported by means of the emerging dual photoredox catalytic system.

Preliminarily, we have also mentioned that the propargylation reaction of aldehydes was also accessible by the dual photoredox-mediated catalysis with titanium,^9,16^ and herein we illustrate the potentiality, cleanness, and simplicity of the propargylation reaction conducted under photoredox conditions by the use of propargyl bromide.

Starting by employing hydrocinnamic aldehyde **1a**, we have optimized the model propargylation reaction using propargyl bromide, and in general, it was found that the reaction was promoted by the organic dyes 3DPAFIPN, affording the product **3a** in good yields. 4CzIPN^17^ and 3CzClIPN^18^ were also tested in the model reactions and gave inferior results (Table 1, entries 3 and 4, respectively). All reactions were performed with the cheap and commercially available [Cp_2_TiCl_2_]. As reported in our allylation reaction,^9a^ the use of THF, with a substrate concentration of 0.05 M, was important to allow the desired transformation due to the strong overlap in the absorbance of the photocatalyst and the titanium complex. The optimized conditions are in line with the photophysical observation because the concentration of the red titanium complex (ε_455nm_ ≈ 250 M^–1^ cm^–1^) allows a significant absorption of the blue photons by 3DPAFIPN (ε_455nm_ ≈ 2900 M^–1^ cm^–1^) to promote the photoinduced electron transfer. Hantzsch’s ester was found to be the best choice as the stoichiometric reductant since various sacrificial reductants (i.e., different amines) were proved to be not suited for the propargylation reaction. This is in part related to the lack of stability of [Cp_2_TiCl_2_] in the presence of different amines under irradiation.^16^ The propargylation reaction was quite sensitive to traces of oxygen and water, and low yields were isolated, performing the reaction in the presence of oxygen (Table 1, entry 8). It is worth noting that the photocatalyst does not decompose during the photoreaction and can be easily recovered at the end of the reaction by flash chromatography.

**Table 1:**
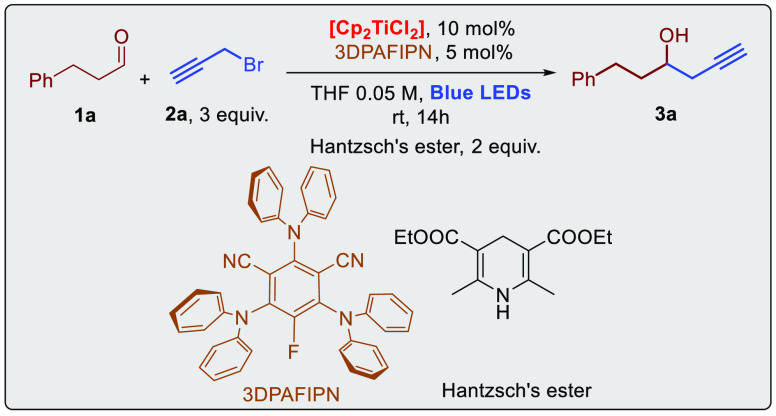
Propargylation Reaction of Hydrocinnamaldehyde and Variation of Some Reaction Parameters

entry^a^	deviations from standard conditions	yield (%)^b^
1^c^	none	>99(98)
2	1.0 mmol scale, 48 h, 3 mol % of 3DPAFIPN	>99(93)
3	4CzIPN instead of 3DPAFIPN	62
4	3CzClIPN instead of 3DPAFIPN	57
5	no Light	0
6	no Hantzsch’s ester	0
7	no [Cp_2_TiCl_2_]	0
8	no degassed solvent	75
9	no photocatalyst, with irradiation at 456 nm	0
10	in the presence of TEMPO (1 equiv)	10
11	DMF instead of THF	traces
12	CH_3_CN instead of THF	55

a Reactions performed on a 0.1 mmol scale irradiating with a 40W Kessil lamp, 456 nm.

b Determined by ^1^H NMR analysis. Values in parentheses represent the yield after chromatographic purification.

c Reaction performed on a 0.2 mmol scale.

It was possible to scale the reaction up to 1.0 mmol, increasing the reaction time to 48 h without observing an appreciable decrease of the yield (Table 1, entry 2).

The selected reaction conditions were employed to test the scope of the reaction with aromatic and aliphatic aldehydes. As evident by the data reported in Scheme 1, aromatic and heteroaromatic aldehydes are suitable substrates for the reaction. Yields are, in general, from good to moderate. The presence of electron-withdrawing groups on the aromatic ring reduced the yield of the reaction. Sterical hindrance in the *ortho*-position does not hamper the reactivity, with either activating or deactivating groups. Although the oxidized product of Hantzsch’s ester (the corresponding protonated pyridinium) is strongly acidic and could favor undesired reaction pathways involving the indole ring, indole substrates are reactive in the propargylation reaction, giving much better yields compared to what was observed for the allylation reaction.^9a^ Variously substituted thiophene carboxaldehydes are suitable substrates for the transformation. As already noted in the allylation reaction, no additives or scavengers are required for the release of the desired homopropargylic alcohol with the concomitant restoration of the titanium complex from homopropargylic alkoxide. As a matter of fact, the protonated pyridine derivative obtained by the oxidation of the Hantzsch’s ester behaves as a scavenger for the reaction, enabling the desired turnover of the employed titanium complex. As was observed for the allylation reaction, also in the photoredox propargylation reaction, 10 mol % of the [Cp_2_TiCl_2_] complex gave the optimal catalyst concentration. In all reported examples with aromatic aldehydes, no traces of the possible allenylic product were detected by ^1^H NMR analysis of the crude reaction mixture. The reaction is also quite effective with aliphatic aldehydes (**3q**–**v**), and excellent results were obtained (Scheme 2). Branched aliphatic aldehydes were found to be reactive substrates, furnishing the respective homopropargylic alcohols in good yields. In addition, aliphatic aldehydes with acidic protons, whose propargylation products can suffer from undesired water elimination pathways, are suitable substrates. No modifications in the conditions were made to perform the reaction with aliphatic aldehydes with respect to aromatic substrates.

**Scheme 1:**
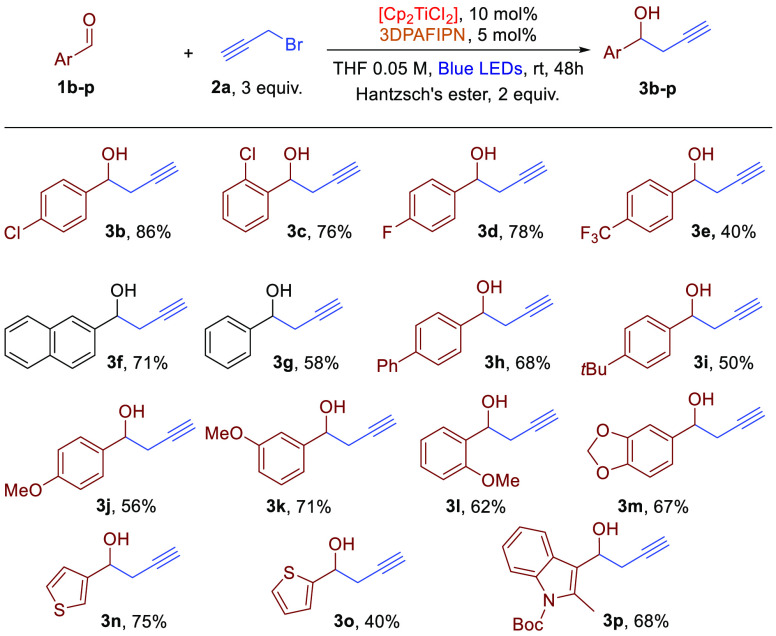
Dual Photoredox Propargylation of Aromatic Aldehydes

**Scheme 2:**
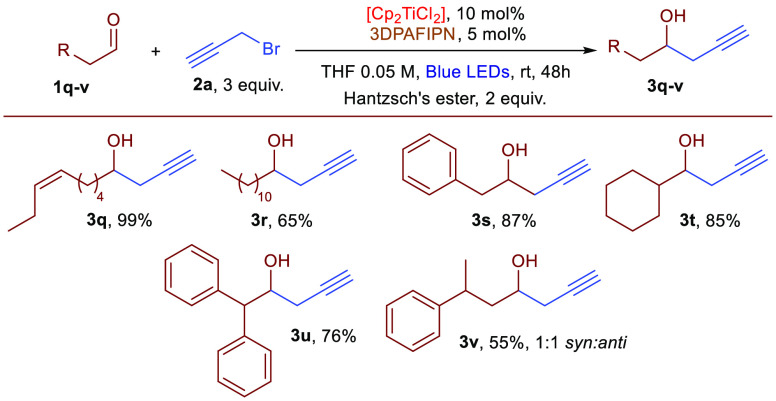
Dual Photoredox Propargylation of Aliphatic Aldehydes

In general, the reactions ran smoothly without any significant inconveniences. Also, with aliphatic aldehydes, the reactions favored the propargylic derivatives in all examined cases.

We have briefly investigated the outcome of the reaction in the case of different propargylic bromides (Scheme 3). Interestingly, the presence of aliphatic or aromatic substituents on the propargylic moiety favors the allenylic product, probably due to the major sterical hindrance of the allenylic titanium intermediate, compared to the propargylic. The synthesis and structural characterizations of allenyl titanocene(IV) and propargyl titanocene(IV) were reported in literature.^19^ These compounds are involved in fast metallotropic allenyl–propargyl equilibria in solution prior to the electrophilic quenching,^20^ as confirmed by DFT calculations. Therefore, the reactivity of differently substituted propargylic halides are controlled by the different energy barriers in transition states relative to the reaction of the propargyl and allenyl titanium(IV) precursors with carbonyls via S_E_2 mechanism and not by the metallotropic equilibria.^19^

**Scheme 3:**
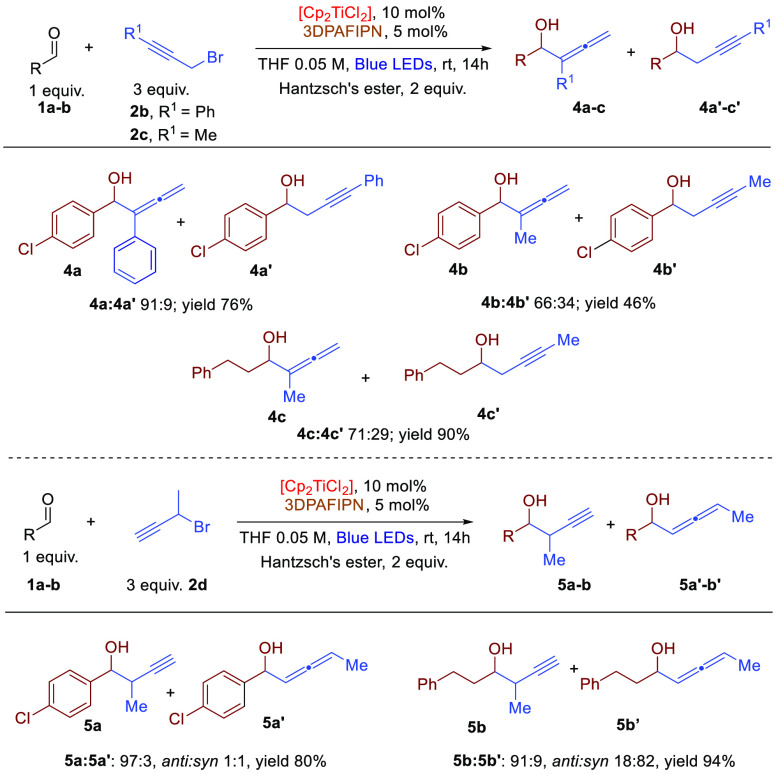
Dual Photoredox Propargylation of Aliphatic Aldehydes

The reaction with secondary propargyl bromides gave unsatisfactory simple diastereoselection; in this case, the result could be imputable to the absence of control in the formation of the allenyl titanium intermediate. We have conducted the Stern–Volmer analysis of the reaction, similarity to our previous study of allylation, by simply varying the concentration of propargyl bromide added to the 3DPAFIPN. As illustrated in Figure S3B (see Supporting Information), in air-equilibrated solution, the emission intensity of 3DPAFIPN is barely decreasing upon increasing the concentration of propargyl bromide, thus highlighting a slow diffusional quenching (*k*_q_ 1.0 × 10^8^ M^–1^ s^–1^).

The same conclusions can be drawn by observing the negligible changes in the emission lifetime in the presence of propargyl bromide at concentrations up to ca. 0.13 M (Figure S3C). In degassed solutions, the long-excited state lifetime of pristine 3DPAFIPN (172 μs) is decreasing to 41 μs upon the addition of propargyl bromide at high concentrations (ca. 0.11 M). The estimated quenching constant is 3 orders of magnitude lower than that determined for [Cp_2_TiCl_2_] in the same experimental conditions (*k*_q_ ≈ 10^5^ and 5.2 × 10^8^ M^–1^ s^–1^, respectively;^8^ see Figure S4B), pointing out that a photoinduced electron transfer is more likely to occur to the latter.

We have already reported^9^ that Hantzsch’s ester and [Cp_2_TiCl_2_] are effective quenchers of the photocatalyst. It is also worth mentioning that we have also established that the pyridine salt formed in the reaction is not a quencher at any concentration. In the case of propargylation, the absence of quenching with propargylic bromide suggests the mechanism illustrated in Figure 1. The oxidative quenching of *3DPAFIPN is responsible for the formation of [Cp_2_TiCl] and 3DPAFIPN^•+^. The latter is a strong oxidant (*E*(PC^•+^/PC) = +1.30 vs SCE),^13^ and the photoredox cycle is closed by the Hantzsch’s ester (*E*(HE^•+^/HE) = +1.0 vs SCE), which reduces the 3DPAFIPN^•+^ back to 3DPAFIPN. The reaction produces the strong reductant HE^•+^ that can participate in further electron transfer events,^21^ generating the rearomatized Hantzsch’s ester, in the form of its pyridinium salt. Furthermore, the oxidative quenching of the photocatalyst in its excited state by the titanium complex [Cp_2_TiCl_2_] generates the [Cp_2_TiCl] species. A radical-mediated addition^22^ of [Cp_2_TiCl] to the propargyl bromide gave the corresponding allenylic/propargylic titanium reagents. Subsequent reaction of the allenylic species with aldehydes gave the titanium-alkoxy derivatives that are transformed into the corresponding alcohols by acidic protons available from the oxidized Hantzsch’s ester pyridinium salt. In fact, the latter is an acidic compound, and it features a low p*K*_a_ compared to other reagents used as scavengers in the catalytic redox reaction promoted by titanium chemistry (such as collidine·HCl).^23^

**Figure 1:**
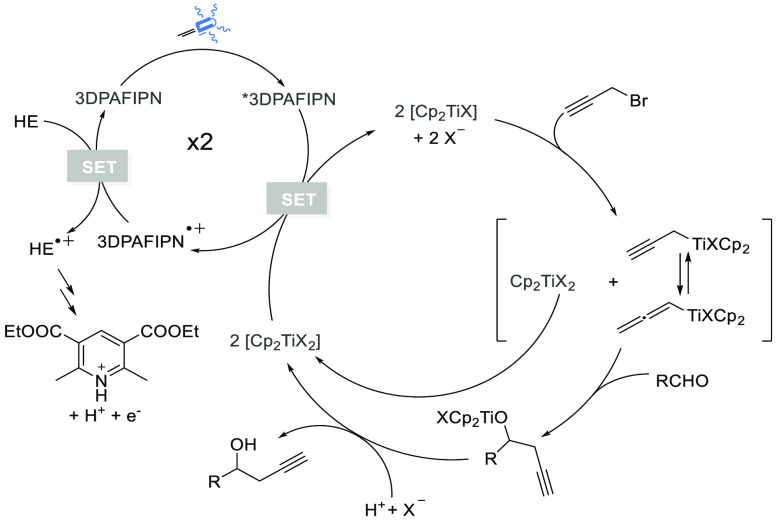
Proposed catalytic cycle.

In summary, we have described a direct photoredox propargylation reaction mediated by a cheap and not toxic titanium complex that affords the desired homopropargylic alcohols in good yields with both aromatic and aliphatic aldehydes. Further studies about metal photoredox-mediated reactions^24^ are in progress in our laboratory.

## Experimental Section

### General Methods and Materials

^1^H NMR, ^13^C NMR, and ^19^F NMR spectra were recorded on a Varian Mercury 400 spectrometer. The residual protic signal of the solvent for the ^1^H and ^13^CDCl_3_ signals for ^13^C were used as references for spectra recorded in CDCl_3_ (7.26 and 77.16 ppm, respectively). The trifluoroacetic acid signal (−76.55 ppm) was used as a reference for ^19^F NMR spectra. Chemical shifts are reported in parts per million (ppm) of the δ scale relative to TMS for ^1^H and ^13^C NMR spectra and CFCl_3_ for ^19^F NMR spectra. Data are reported as follows: chemical shift, multiplicity (s = singlet, d = doublet, t = triplet, dd = doublet of doublet, ddd = doublet of doublet of doublet, td = triplet of doublet, m = multiplet), coupling constants (Hz). Chromatographic purifications were performed with 240–400 mesh silica gel. HPLC-MS analyses were performed on an Agilent Technologies HP1100 instrument coupled with an Agilent Technologies MSD1100 single-quadrupole mass spectrometer using a Phenomenex Gemini C18 3 μm (100 mm × 3 mm) column; mass spectrometric detection was performed as follows: in full-scan mode from *m*/*z* 50 to 2500, scan time 0.1 s in positive ion mode, ESI spray voltage 4500 V, nitrogen gas 35 psi, drying gas flow rate 11.5 mL min^–1^, and fragmentor voltage 30 V. HRMS was performed on a Waters Xevo G2-XS QTof, ESI^+^, cone voltage 40 V, Capillary 3KV, with a source temperature of 120 °C. All reactions were set up under an argon atmosphere in oven-dried glassware (borosilicate) using standard Schlenk techniques. The reaction mixture was irradiated with a Kessil PR160L@456 nm. Lamp technical specifications are available on the manufacturer’s web site.^25^ The reaction vessel was placed 10 cm approximately from the lamp, and the temperature was maintained at room temperature by cooling with a PR160 ring w/fan kit.^26^ 3DPAFIPN,^13^ 4CzIPN,^13^ 3CzClIPN,^13^ and diethyl 2,6-dimethyl-1,4-dihydropyridine-3,5-dicarboxylate (Hantzsch’s Ester)^27^ were prepared following literature procedures.

### Standard Procedure for Photoredox Titanium-Catalyzed Propargylation of Aldehydes

All reactions were performed on a 0.2 mmol scale of aldehyde. A dry 10 mL Schlenk tube, equipped with a Rotaflo stopcock, magnetic stirring bar, and argon supply tube, was first charged under argon with the organic photocatalyst 2,4,6-tris(diphenylamino)-5-fluoroisophthalonitrile **3DPAFIPN** (5 mol %, 0.010 mmol, 6.4 mg), [Cp_2_TiCl_2_] catalyst (10 mol %, 0.02 mmol, 5.0 mg), and diethyl 1,4-dihydro-2,6-dimethyl-3,5-pyridinedicarboxylate, i.e. Hantzsch’s ester (2 equiv, 0.4 mmol, 100 mg). Inhibitor-free dry THF (4 mL, in order to obtain a 0.05 M solution of aldehyde) was then added, the reaction mixture was further subjected to a freeze–pump–thaw procedure (three cycles), and the vessel was refilled with argon. Then, propargyl bromide derivative **2a**–**d** (0.6 mmol, 3 equiv) and the substrate **1a**–**v** (0.2 mmol) were added. The reaction was irradiated under vigorous stirring for 14 h. After that, the reaction mixture was quenched with H_2_O (approximately 5 mL) and extracted with AcOEt (4 × 5 mL). The combined organic layers were dried over anhydrous Na_2_SO_4_, and the solvent was removed under reduced pressure. The crude was subject of flash column chromatography (SiO_2_) to afford the products **3a**–**v**, **4a**–**c**, and **5a**–**b** in the stated yields.

### Procedure for 1 mmol Scale

A dry 50 mL Schlenk tube, equipped with a magnetic stirring bar and argon supply tube, was first charged under argon with the organic photocatalyst 3DPAFIPN 2,4,6-tris(diphenylamino)-5-fluoroisophthalonitrile (3 mol %, 0.03 mmol, 19 mg), [Cp_2_TiCl_2_] catalyst (8 mol %, 0.08 mmol, 0.020 g), and diethyl 1,4-dihydro-2,6-dimethyl-3,5-pyridinedicarboxylate, namely, Hantzsch’s ester (2 equiv, 2 mmol, 0.500 g). Inhibitor-free dry THF (20 mL in order to obtain a 0.05 M solution of aldehyde) was then added, the reaction mixture was further subjected to a freeze–pump–thaw procedure (four cycles), and the vessel refilled with argon. Then, propargyl bromide **2a** (80% v/v in toluene, 3 mmol, 3 equiv, 0.280 mL) and the substrate **1a** (1 mmol, 0.134 g, 0.132 mL) were added. The reaction was irradiated under vigorous stirring for 48 h. After that, the solvent was removed under reduced pressure. The crude was subjected to flash column chromatography (SiO_2_) to afford the products **3a** in 93% yield (0.93 mmol, 0.162 g).

### 1-Phenylhex-5-yn-3-ol (**3a**):

brown oil, 98% (0.195 mmol, 34 mg). The general procedure was applied using **1a** (0.2 mmol, 26 μL) previously distilled and **2a** (solution 80% v/v in toluene, 0.6 mmol, 56 μL, 3 equiv). The title compound was isolated by flash column chromatography (100% DCM). Spectroscopic data were in agreement with those reported in literature.^28^

### 1-(4-Chlorophenyl)but-3-yn-1-ol (**3b**):

brown oil, 86% (0.17 mmol, 31 mg). The general procedure was applied using **1b** (0.2 mmol, 28 mg) and **2a** (solution 80% v/v in toluene, 0.6 mmol, 56 μL, 3 equiv). The title compound was isolated by flash column chromatography (100% DCM). Spectroscopic data were in agreement with those reported in literature.^29^

### 1-(2-Chlorophenyl)but-3-yn-1-ol (**3c**):

brown oil, 76% (0.15 mmol, 27 mg). The general procedure was applied using **1c** (0.2 mmol, 22.4 μL) previously distilled and **2a** (solution 80% v/v in toluene, 0.6 mmol, 56 μL, 3 equiv). The title compound was isolated by flash column chromatography (100% DCM). Spectroscopic data were in agreement with those reported in literature.^28^

### 1-(4-Fluorophenyl)but-3-yn-1-ol (**3d**):

brown oil, 78% (0.16 mmol, 22.3 mg). The general procedure was applied using **1d** (0.2 mmol, 22 μL) previously distilled and **2a** (solution 80% v/v in toluene, 0.6 mmol, 56 μL, 3 equiv). The title compound was isolated by flash column chromatography (100% DCM). Spectroscopic data were in agreement with those reported in literature.^30^

### 1-(4-(Trifluoromethyl)phenyl)but-3-yn-1-ol (**3e**):

brown oil, 40% (0.08 mmol, 18 mg). The general procedure was applied using **1e** (0.2 mmol, 28 μL) previously distilled and **2a** (solution 80% v/v in toluene, 0.6 mmol, 56 μL, 3 equiv). The title compound was isolated by flash column chromatography (100% DCM). Spectroscopic data were in agreement with those reported in literature.^31^

### 1-(Naphthalen-2-yl)but-3-yn-1-ol (**3f**):

brown oil, 71% (0.14 mmol, 28 mg). The general procedure was applied using **1f** (0.2 mmol, 32 mg) and **2a** (solution 80% v/v in toluene, 0.6 mmol, 56 μL, 3 equiv). The title compound was isolated by flash column chromatography (100% DCM). Spectroscopic data were in agreement with those reported in literature.^8^

### 1-Phenylbut-3-yn-1-ol (**3g**):

brown oil, 58% (0.12 mmol, 18 mg). The general procedure was applied using **1g** (0.2 mmol, 20.4 μL) previously distilled and (solution 80% v/v in toluene, 0.6 mmol, 56 μL, 3 equiv). The title compound was isolated by flash column chromatography (100% DCM). Spectroscopic data were in agreement with those reported in literature.^32^

### 1-([1,1′-Biphenyl]-4-yl)but-3-yn-1-ol (**3h**):

brown oil, 68% (0.14 mmol, 30.2 mg). The general procedure was applied using **1h** (0.2 mmol, 36 mg) and **2a** (solution 80% v/v in toluene, 0.6 mmol, 56 μL, 3 equiv). The title compound was isolated by flash column chromatography (100% DCM). Spectroscopic data were in agreement with those reported in literature.^33^

### 1-(4-(*tert*-Butyl)phenyl)but-3-yn-1-ol (**3i**):

brown oil, 50% (0.1 mmol, 20.2 mg). The general procedure was applied using **1i** (0.2 mmol, 32 μL) previously distilled and **2a** (solution 80% v/v in toluene, 0.6 mmol, 56 μL, 3 equiv). The title compound was isolated by flash column chromatography (100% DCM). Spectroscopic data were in agreement with those reported in literature.^34^

### 1-(4-Methoxyphenyl)but-3-yn-1-ol (**3j**):

brown oil, 56% (0.11 mmol, 19 mg). The general procedure was applied using **1j** (0.2 mmol, 26 μL) and **2a** (solution 80% v/v in toluene, 0.6 mmol, 56 μL, 3 equiv). The title compound was isolated by flash column chromatography (100% DCM). Spectroscopic data were in agreement with those reported in literature.^35^

### 1-(3-Methoxyphenyl)but-3-yn-1-ol (**3k**):

brown oil, 71% (0.14 mmol, 25 mg). The general procedure was applied using **1k** (0.2 mmol, 26 μL) and **2a** (solution 80% v/v in toluene, 0.6 mmol, 56 μL, 3 equiv). The title compound was isolated by flash column chromatography (100% DCM). Spectroscopic data were in agreement with those reported in literature.^29^

### 1-(2-Methoxyphenyl)but-3-yn-1-ol (**3l**):

brown oil, 62% (0.12 mmol, 22 mg). The general procedure was applied using **1l** (0.2 mmol, 26 μL) and **2a** (solution 80% v/v in toluene, 0.6 mmol, 56 μL, 3 equiv). The title compound was isolated by flash column chromatography (100% DCM). Spectroscopic data were in agreement with those reported in literature.^31^

### 1-(Benzo[*d*][1,3]dioxol-5-yl)but-3-yn-1-ol (**3m**):

brown oil, 67% (0.13 mmol, 25 mg). The general procedure was applied using **1m** (0.2 mmol, 38 mg) and **2a** (solution 80% v/v in toluene, 0.6 mmol, 56 μL, 3 equiv). The title compound was isolated by flash column chromatography(100% DCM). Spectroscopic data were in agreement with those reported in literature.^29^

### 1-(Thiophen-3-yl)but-3-yn-1-ol (**3n**):

brown oil, 75% (0.15 mmol, 23 mg). The general procedure was applied using **1n** (0.2 mmol, 18 μL) and **2a** (solution 80% v/v in toluene, 0.6 mmol, 56 μL, 3 equiv). The title compound was isolated by flash column chromatography (100% DCM). Spectroscopic data were in agreement with those reported in literature.^36^

### 1-(Thiophen-2-yl)but-3-yn-1-ol (**3o**):

brown oil, 40% (0.08 mmol, 12 mg). The general procedure was applied using **1o** (0.2 mmol, 18 μL) and **2a** (solution 80% v/v in toluene, 0.6 mmol, 56 μL, 3 equiv). The title compound was isolated by flash column chromatography (100% DCM). Spectroscopic data were in agreement with those reported in literature.^32^

### *tert*-Butyl 3-(1-Hydroxybut-3-yn-1-yl)-2-methyl-1*H*-indole-1-carboxylate (**3p**):

brown oil, 68% (0.14 mmol, 41 mg). The general procedure was applied using **1p** (0.2 mmol, 52 mg) and **2a** (solution 80% v/v in toluene, 0.6 mmol, 56 μL, 3 equiv). The title compound was isolated by flash column chromatography (100% DCM). ^1^H NMR (401 MHz, CDCl_3_): δ 8.10 (d, *J* = 8.0 Hz, 1H), 7.77 (d, *J* = 7.6 Hz, 1H), 7.30–7.16 (m, 2H overlapped with the residual peak of the solvent), 5.22 (dd, *J* = 7.9, 6.2 Hz, 1H), 2.96 (ddd, *J* = 16.7, 8.2, 2.4 Hz, 1H), 2.68 (ddd, *J* = 16.7, 5.8, 2.4 Hz, 1H), 2.60 (s, 3H), 2.35 (s, 1H), 2.06 (t, *J* = 2.3 Hz, 1H), 1.68 (s, 9H). ^13^C{^1^H} NMR (101 MHz, CDCl_3_): δ 150.6, 136.0, 134.2, 127.3, 123.5, 122.4, 119.4, 118.4, 115.4, 83.9, 80.9, 70.5, 67.1, 28.2, 27.4, 14.2. HRMS (ESI/Q-TOF): *m*/*z* [M-Boc-H_2_O + H]^+^ calcd for C_13_H_11_N, 181.0891; found, 182.0961. HRMS (ESI/Q-TOF): *m*/*z* [M + Na]^+^ calcd for C_18_H_21_NO_3_Na, 322.1419, found, 322.1409.

### (*Z*)-Dodec-9-en-1-yn-4-ol (**3q**):

yellow oil, 99% (0.2 mmol, 36 mg). The general procedure was applied using **1q** (0.2 mmol, 34 μL) and **2a** (solution 80% v/v in toluene, 0.6 mmol, 56 μL, 3 equiv). The title compound was isolated by flash column chromatography (100% DCM). ^1^H NMR (401 MHz, CDCl_3_): δ 5.50–5.19 (m, 2H), 3.80–3.71 (m, 1H), 2.43 (ddd, *J* = 16.7, 4.7, 2.6 Hz, 1H), 2.31 (ddd, *J* = 16.7, 6.7, 2.6 Hz, 1H), 2.11–1.98 (m, 5H), 1.97–1.85 (m, 1H), 1.57–1.51 (m, 2H), 1.44–1.35 (m, 3H), 1.25 (m, 1H), 0.95 (td, *J* = 7.5, 2.2 Hz, 3H). ^13^C{^1^H} NMR (101 MHz, CDCl_3_): δ 131.8, 128.8, 80.8, 70.7, 69.8, 36.1, 29.6, 27.3, 26.9, 25.2, 20.5, 14.3. ESI-MS: *m*/*z* 198.2 [M + NH_4_]^+^. HRMS (ESI/Q-TOF): *m*/*z* [M + NH_4_]^+^ calcd for C_12_H_24_NO, 198.1858; found, 198.1853.

### Pentadec-1-yn-4-ol (**3r**):

brown oil, 65% (0.13 mmol, 25 mg). The general procedure was applied using **1r** (0.2 mmol, 32 μL) and **2a** (solution 80% v/v in toluene, 0.6 mmol, 56 μL, 3 equiv). The title compound was isolated by flash column chromatography (100% DCM). Spectroscopic data were in agreement with those reported in literature.^37^

### 1-Phenylpent-4-yn-2-ol (**3s**):

brown oil, 87% (0.17 mmol, 28 mg). The general procedure was applied using **1s** (0.2 mmol, 23 μL) and **2a** (solution 80% v/v in toluene, 0.6 mmol, 56 μL, 3 equiv). The title compound was isolated by flash column chromatography (100% DCM). Spectroscopic data were in agreement with those reported in literature.^38^

### 1-Cyclohexylbut-3-yn-1-ol (**3t**):

brown oil, 85% (0.17 mmol, 26 mg). The general procedure was applied using **1t** (0.2 mmol, 22 μL) and **2a** (solution 80% v/v in toluene, 0.6 mmol, 56 μL, 3 equiv). The title compound was isolated by flash column chromatography (100% DCM). Spectroscopic data were in agreement with those reported in literature.^29^

### 1,1-Diphenylpent-4-yn-2-ol (**3u**):

brown oil, 76% (0.15 mmol, 36 mg). The general procedure was applied using **1u** (0.2 mmol, 39.2 mg) and **2a** (solution 80% v/v in toluene, 0.6 mmol, 56 μL, 3 equiv). The title compound was isolated by flash column chromatography (100% DCM). ^1^H NMR (401 MHz, CDCl_3_): δ 7.41 (dd, *J* = 8.2, 1.0 Hz, 2H), 7.37–7.18 (m, 8H), 4.54 (ddd, *J* = 8.7, 6.3, 4.5 Hz, 1H), 4.13 (d, *J* = 8.7 Hz, 1H), 2.46 (ddd, *J* = 16.9, 4.3, 2.7 Hz, 1H), 2.30 (ddd, *J* = 16.9, 6.4, 2.7 Hz, 1H), 2.17–2.08 (m, 1H), 2.04 (s, 1H). ^13^C{^1^H} NMR (101 MHz, CDCl_3_): δ 141.7, 140.6, 128.8 (3C), 128.7 (2C), 128.6 (2C), 128.2, 126.8, 126.9, 80.6, 71.7, 71.1, 56.9, 25.3. HRMS (ESI/Q-TOF) *m*/*z*: [M-H_2_O + H]^+^ calcd for C_17_H_15_N, 219.1174, found, 219.1164.

### 6-Phenylhept-1-yn-4-ol (**3v**):

brown oil, 55% (0.11 mmol, 20 mg) as a *syn*/*anti* mixture, dr of 1:1. The general procedure was applied using **1v** (0.2 mmol, 29.6 mg) and **2a** (solution 80% v/v in toluene, 0.6 mmol, 56 μL, 3 equiv). The title compound was isolated by flash column chromatography (100% DCM). In analogy with the reported data in literature for similar compounds,^39^ it was possible to distinguish the *syn* and the *anti* isomer. The diastereoisomeric ratio was calculated, considering the ^1^H NMR spectrum of the reaction crude, comparing the integral of the signal at 3.77 ppm for the *syn* product and at 3.49 for the *anti* product. **3v**_syn_^1^H NMR (401 MHz, CDCl_3_): δ 7.33–7.18 (m, 5H, overlapped with the residual peak of the solvent and peaks related to the other isomer aromatic protons), 3.77 (s, 1H), 3.09–2.97(m, 1H), 2.52–2.39 (m, 1H), 2.05 (t, *J* = 12.0, 2.6 Hz, 2H), 1.93–1.83 (m, 3H), 1.33–1.29 (m, 3H). ^13^C NMR (101 MHz, CDCl_3_): δ 146.9, 128.6 (2H), 127.1(2H), 126.2, 80.8, 70.9, 68.0, 44.6, 36.5, 27.9, 23.1. **3v**_anti_^1^H NMR (401 MHz, CDCl_3_): δ 7.33–7.18 (m, 5H, overlapped with the residual peak of the solvent and peaks related to the other isomer aromatic protons), 3.49 (s, 1H), 2.96–2.87(m, 1H), 2.37–2.20 (m, 1H), 2.02 (t, *J* = 12.0, 2.6 Hz, 2H), 1.82–1.74 (m, 3H), 1.28–1.24 (m, 3H). ^13^C{^1^H} NMR (101 MHz, CDCl_3_): δ 146.3, 128.5 (2H), 126.8 (2H), 126.1, 80.6, 70.8, 67.7, 44.5, 36.4, 27.4, 22.0. HRMS (ESI/Q-TOF) *m*/*z*: [M-H_2_O + H]^+^ calcd for C_13_H_15_, 171.1174; found, 171.1160.

### 1-(4-Chlorophenyl)-2-phenylbuta-2,3-dien-1-ol and 1-(4-Chlorophenyl)-4-phenylbut-3-yn-1-ol (**4a**–**4a**′):

brown oil, 76% (0.14 mmol, 36 mg) as a mixture regioisomers **4a**/**4a′** (91:9). The general procedure was applied using **1b** (0.2 mmol, 28 mg) and **2b** (0.6 mmol, 117 mg, 3 equiv). The ratio of regioisomer was calculated considering the ^1^H NMR spectrum of the reaction crude and comparing the integral of the signal at 5.71 ppm for **4a** and at 4.91 ppm for **4a′**. The title compound was isolated by flash column chromatography (100% DCM). Spectroscopic data were in agreement with those reported in literature.^40^

### 1-(4-Chlorophenyl)-2-methylbuta-2,3-dien-1-ol and 1-(4-chlorophenyl)pent-3-yn-1-ol (**4b**–**4b**′):

brown oil, 46% (0.1 mmol, 18 mg) as a mixture of regioisomers **4b**/**4b′** (66:34). The general procedure was applied using **1b** (0.2 mmol, 28 mg) and **2c** (0.6 mmol, 52 μL, 3 equiv). The regioisomeric ratio was calculated considering the ^1^H NMR spectrum of the reaction crude and comparing the integral of the signal at 5.10 ppm for **4b** and at 4.77 ppm for **4b′**. The title compound was isolated by flash column chromatography (100% DCM). Spectroscopic data were in agreement with those reported in literature.^41^

### 4-Methyl-1-phenylhexa-4,5-dien-3-ol and 1-phenylhept-5-yn-3-ol (**4c**–**4c**′):

brown oil, 90% (0.18 mmol, 34 mg) as a mixture of regioisomers **4c**/**4c′** (71:29). The general procedure was applied using **1v** (0.2 mmol, 26 μL) and **2c** (0.6 mmol, 52 μL, 3 equiv). The regioisomeric ratio was calculated considering the ^1^H NMR spectrum of the reaction crude and comparing the integral of the signal at 4.81 ppm related for **4c** and at 4.72 ppm for **4c′**. The title compound was isolated by flash column chromatography (100% DCM). Spectroscopic data were in agreement with those reported in literature.^42^

### 1-(4-Chlorophenyl)-2-methylbut-3-yn-1-ol and 1-(4-chlorophenyl)penta-2,3-dien-1-ol (**5a**–**5a**′):

brown oil, 80% (0.16 mmol, 31 mg) as a mixture of regioisomers and diastereoisomers **5a**/**5a′** of 97:3, **5a**_syn_/**5a**_anti_ dr of 1:1. The regioisomeric ratio was calculated considering the ^1^H NMR spectrum of the reaction crude and comparing the integrals of the signal at 4.71 ppm for **5a** and at 5.29 ppm for **5a′**. The diastereoisomeric ratio was calculated considering the ^1^H NMR spectrum of the reaction crude comparing the integral of the signal at 4.71 ppm for the product **5a**_syn_ and at 4.51 ppm for the product **5a**_anti_. The general procedure was applied using **1b** (0.2 mmol, 28 mg) and **2d** (0.6 mmol, 79 mg, 3 equiv). The title compound was isolated by flash column chromatography (100% DCM). Spectroscopic data were in agreement with those reported in literature.^43^

### 4-Methyl-1-phenylhex-5-yn-3-ol and 1-phenylhepta-4,5-dien-3-ol (**5b**–**5b**′):

brown oil, 94% (0.19 mmol, 35 mg) as a mixture of regioisomers and diastereoisomers of **5b**/**5b′** of 92:8, **5b**_syn_/**5b**_anti_ dr of 18:82. The regioisomeric ratio was calculated, considering the ^1^H NMR spectrum of the reaction crude, comparing the integral of the signal at 3.49 ppm for **5b** and at 4.80 ppm for **5b′**. The diastereoisomeric ratio was calculated, considering the ^1^H NMR spectrum of the reaction crude, comparing the integral of the signal at 3.54 ppm related to the product **5b**_syn_ and the multiplet (1H) at 3.49 ppm related to the product **5b**_anti_. The general procedure was applied using **1a** (0.2 mmol, 26 μL) and **2d** (0.6 mmol, 79 mg, 3 equiv). The title compound was isolated by flash column chromatography (100% DCM). Spectroscopic data were in agreement with those reported in literature.^44^

### Photophysical and Mechanistic Studies

All photophysical analyses were carried out in air-equilibrated tetrahydrofuran at 298 K unless otherwise specified. UV–vis absorption spectra were recorded with a PerkinElmer λ40 spectrophotometer using quartz cells with an optical path length of 1.0 cm. Degassed solutions are obtained by means of repeated pump–freeze–thaw cycles (ca. 4 × 10^–6^ mbar) in sealed quartz cuvettes. Luminescence spectra were performed with a PerkinElmer LS-50, a Varian Cary Eclipse, or an Edinburgh FLS920 spectrofluorimeter equipped with a Hamamatsu R928 phototube. Lifetimes shorter than 10 μs were measured by the above-mentioned Edinburgh FLS920 spectrofluorimeter equipped with a TCC900 card for data acquisition in time-correlated single-photon counting experiments (0.5 ns time resolution). The estimated experimental errors are 2 nm on the band maximum, 5% on the molar absorption coefficient, and luminescence lifetime.

## Supporting Information Available

Photos of reaction setup, copies of NMR spectra, and photophysical study results (PDF)

## References

[ref1] Nicewicz D. A.; MacMillan D. W. C. Merging Photoredox Catalysis with Organocatalysis: The Direct Asymmetric Alkylation of Aldehydes. Science 2008, 322 (5898), 77–80. 10.1126/science.1161976. 18772399 PMC2723798

[ref2] aStrieth-Kalthoff F.; James M. J.; Teders M.; Pitzer L.; Glorius F. Energy transfer catalysis mediated by visible light: principles, applications, directions. Chem. Soc. Rev. 2018, 47, 7190–7202. 10.1039/C8CS00054A. 30088504

[ref3] Twilton J.; Le C. C.; Zhang P.; Shaw M. H.; Evans R. W.; MacMillan D. W. C. The Merger of Transition Metal and Photocatalysis. Nat. Rev. Chem. 2017, 1, 0052 10.1038/s41570-017-0052.

[ref4] Zhang H. H.; Chen H.; Zhu C.; Yu S. A Review of Enantioselective Dual Transition Metal/Photoredox Catalysis. Sci. China: Chem. 2020, 63 (5), 637–647. 10.1007/s11426-019-9701-5.

[ref5] Pitzer L.; Schwarz J. L.; Glorius F. Reductive Radical-Polar Crossover: Traditional Electrophiles in Modern Radical Reactions. Chem. Sci. 2019, 10 (36), 8285–8291. 10.1039/C9SC03359A. 32055300 PMC7003961

[ref6] Wiles R. J.; Molander G. A. Photoredox-Mediated Net-Neutral Radical/Polar Crossover Reactions. Isr. J. Chem. 2020, 60 (3–4), 281–293. 10.1002/ijch.201900166. 33986554 PMC8115720

[ref7] aSchwarz J. L.; Schäfers F.; Tlahuext-Aca A.; Lückemeier L.; Glorius F. Diastereoselective Allylation of Aldehydes by Dual Photoredox and Chromium Catalysis. J. Am. Chem. Soc. 2018, 140 (40), 12705–12709. 10.1021/jacs.8b08052. 30216059

[ref8] aGualandi A.; Rodeghiero G.; Faraone A.; Patuzzo F.; Marchini M.; Calogero F.; Perciaccante R.; Jansen T. P.; Ceroni P.; Cozzi P. G. Allylation of Aldehydes by Dual Photoredox and Nickel Catalysis. Chem. Commun. 2019, 55 (48), 6838–6841. 10.1039/C9CC03344K. 31093623

[ref9] aGualandi A.; Calogero F.; Mazzarini M.; Guazzi S.; Fermi A.; Bergamini G.; Cozzi P. G. Cp_2_TiCl_2_-Catalyzed Photoredox Allylation of Aldehydes with Visible Light. ACS Catal. 2020, 10 (6), 3857–3863. 10.1021/acscatal.0c00348.

[ref10] Castro Rodriguez M.; Rodriguez Garcia I.; Rodriguez Maecker R. N.; Pozo Morales L.; Oltra J. E.; Rosales Martinez A. Cp_2_TiCl: An Ideal Reagent for Green Chemistry?. Org. Process Res. Dev. 2017, 21 (7), 911–923. 10.1021/acs.oprd.7b00098.

[ref11] Zhang Z.; Richrath R. B.; Gansäuer A. Merging Catalysis in Single Electron Steps with Photoredox Catalysis—Efficient and Sustainable Radical Chemistry. ACS Catal. 2019, 9, 3208–3212. 10.1021/acscatal.9b00787.

[ref12] Zhang Z.; Hilche T.; Slak D.; Rietdijk N. R.; Oloyede U. N.; Flowers R. A.; Gansäuer A. Titanocenes as Photoredox Catalysts Using Green-Light Irradiation. Angew. Chem., Int. Ed. 2020, 59 (24), 9355–9359. 10.1002/anie.202001508. PMC7317808 32216162

[ref13] Speckmeier E.; Fischer T. G.; Zeitler K. A Toolbox Approach to Construct Broadly Applicable Metal-Free Catalysts for Photoredox Chemistry: Deliberate Tuning of Redox Potentials and Importance of Halogens in Donor-Acceptor Cyanoarenes. J. Am. Chem. Soc. 2018, 140 (45), 15353–15365. 10.1021/jacs.8b08933. 30277767

[ref14] Ding C. H.; Hou X. L. Catalytic Asymmetric Propargylation. Chem. Rev. 2011, 111 (3), 1914–1937. 10.1021/cr100284m. 21344874

[ref15] Huang H. M.; Bellotti P.; Daniliuc C. G.; Glorius F. Radical Carbonyl Propargylation by Dual Catalysis. Angew. Chem., Int. Ed. 2021, 60 (5), 2464–2471. 10.1002/anie.202011996. 33022838

[ref16] Li F. S.; Chen Y. Q.; Lin S. J.; Shi C. Z.; Li X. Y.; Sun Y. C.; Guo Z. W.; Shi L. Visible-Light-Mediated Barbier Allylation of Aldehydes and Ketones: Via Dual Titanium and Photoredox Catalysis. Org. Chem. Front. 2020, 7 (21), 3434–3438. 10.1039/D0QO00171F.

[ref17] Luo J.; Zhang J. Donor-Acceptor Fluorophores for Visible-Light-Promoted Organic Synthesis: Photoredox/Ni Dual Catalytic C(sp^3^)-C(sp^2^) Cross-Coupling. ACS Catal. 2016, 6 (2), 873–877. 10.1021/acscatal.5b02204.

[ref18] Fermi A.; Gualandi A.; Bergamini G.; Cozzi P. G. Shining Light on Ti^IV^ Complexes: Exceptional Tools for Metallaphotoredox Catalysis. Eur. J. Org. Chem. 2020, 2020 (45), 6955–6965. 10.1002/ejoc.202000966.

[ref19] Ruiz-Muelle A. B.; Oña-Burgos P.; Ortuño M. A.; Oltra E. J.; Rodríguez-García I.; Fernàndez I. Unprecedented Spectroscopic and Computational Evidence for Allenyl and Propargyl Titanocene(IV) Complexes: Electrophilic Quenching of Their Metallotropic Equilibrium. Chem. - Eur. J. 2016, 22, 2427–2439. 10.1002/chem.201504281. 26786999

[ref20] Wisniewska H. M.; Jarvo E. R. Enantioselective Propargylation and Allenylation Reactions of Ketones and Imines. J. Org. Chem. 2013, 78, 11629–11636. 10.1021/jo4019107. 24266761

[ref21] Wang P. Z.; Chen J. R.; Xiao W. J. Hantzsch Esters: An Emerging Versatile Class of Reagents in Photoredox Catalyzed Organic Synthesis. Org. Biomol. Chem. 2019, 17 (29), 6936–6951. 10.1039/C9OB01289C. 31268084

[ref22] aMcCallum T.; Wu X.; Lin S. Recent Advances in Titanium Radical Redox Catalysis. J. Org. Chem. 2019, 84 (22), 14369–14380. 10.1021/acs.joc.9b02465. 31647872

[ref23] aGansäuer A.; Pierobon M.; Bluhm H. Catalytic, Highly Regio- and Chemoselective Generation of Radicals from Epoxides: Titanocene Dichloride as an Electron Transfer Catalyst in Transition Metal Catalyzed Radical Reactions. Angew. Chem., Int. Ed. 1998, 37, 101–103. 10.1002/(SICI)1521-3773(19980202)37:1/2<101::AID-ANIE101>3.0.CO;2-W.

[ref24] aGualandi A.; Rodeghiero G.; Perciaccante R.; Jansen T. P.; Moreno-Cabrerizo C.; Foucher C.; Marchini M.; Ceroni P.; Cozzi P. G. Catalytic Photoredox Allylation of Aldehydes Promoted by a Cobalt Complex. Adv. Synth. Catal. 2021, 363 (4), 1105–1111. 10.1002/adsc.202001250.

[ref25] https://www.kessil.com/science/PR160L.php.

[ref26] https://www.kessil.com/science/PR160Rig.php.

[ref27] Schneider L. M.; Schmiedel V. M.; Pecchioli T.; Lentz D.; Merten C.; Christmann M. Asymmetric Synthesis of Carbocyclic Propellanes. Org. Lett. 2017, 19, 2310–2313. 10.1021/acs.orglett.7b00836. 28445060

[ref28] Liang T.; Woo S. K.; Krische M. J. C-Propargylation Overrides O-Propargylation in Reactions of Propargyl Chloride with Primary Alcohols: Rhodium-Catalyzed Transfer Hydrogenation. Angew. Chem., Int. Ed. 2016, 55 (32), 9207–9211. 10.1002/anie.201603575. PMC4965293 27321353

[ref29] Jain P.; Wang H.; Houk K. N.; Antilla J. C. Brønsted Acid Catalyzed Asymmetric Propargylation of Aldehydes. Angew. Chem., Int. Ed. 2012, 51 (6), 1391–1394. 10.1002/anie.201107407. PMC3334334 22223476

[ref30] Reddy L. R. Chiral Brønsted Acid Catalyzed Enantioselective Propargylation of Aldehydes with Allenylboronate. Org. Lett. 2012, 14 (4), 1142–1145. 10.1021/ol300075n. 22273041

[ref31] Chen J.; Captain B.; Takenaka N. Helical Chiral 2,2’-Bipyridine N-Monoxides as Catalysts in the Enantioselective Propargylation of Aldehydes with Allenyltrichlorosilane. Org. Lett. 2011, 13 (7), 1654–1657. 10.1021/ol200102c. 21366250

[ref32] Trost B. M.; Ngai M. Y.; Dong G. Ligand-Accelerated Enantioselective Propargylation of Aldehydes via Allenylzinc Reagents. Org. Lett. 2011, 13 (8), 1900–1903. 10.1021/ol200043n. 21391717

[ref33] Li Y.; Brand J. P.; Waser J. Gold-Catalyzed Regioselective Synthesis of 2- and 3-Alkynyl Furans. Angew. Chem., Int. Ed. 2013, 52 (26), 6743–6747. 10.1002/anie.201302210. 23666679

[ref34] Borowiecki P.; Dranka M. A Facile Lipase-Catalyzed KR Approach toward Enantiomerically Enriched Homopropargyl Alcohols. Bioorg. Chem. 2019, 93, 102754–102769. 10.1016/j.bioorg.2019.01.050. 30765117

[ref35] Vaganov V. Y.; Fukazawa Y.; Kondratyev N. S.; Shipilovskikh S. A.; Wheeler S. E.; Rubtsov A. E.; Malkov A. V. Optimization of Catalyst Structure for Asymmetric Propargylation of Aldehydes with Allenyltrichlorosilane. Adv. Synth. Catal. 2020, 362 (23), 5467–5474. 10.1002/adsc.202000936.

[ref36] Xu M.; Ren T. T.; Wang K. B.; Li C. Y. Synthesis of Cyclobutanones via Gold-Catalyzed Oxidative Rearrangement of Homopropargylic Ethers. Adv. Synth. Catal. 2013, 355 (13), 2488–2494. 10.1002/adsc.201300227.

[ref37] Motodate S.; Kobayashi T.; Fujii M.; Mochida T.; Kusakabe T.; Katoh S.; Akita H.; Kato K. Synthesis of β-Methoxyacrylate Natural Products Based on Box-Pd^II^-catalyzed Intermolecular Methoxycarbonylation of Alkynoles. Chem. - Asian J. 2010, 5 (10), 2221–2230. 10.1002/asia.201000292. 20669219

[ref38] Kim J.; Jeong W.; Rhee Y. H. Flexible Tetrahydropyran Synthesis from Homopropargylic Alcohols Using Sequential Pd-Au Catalysis. Org. Lett. 2017, 19 (1), 242–245. 10.1021/acs.orglett.6b03532. 28004942

[ref39] Arai N.; Satoh H.; Komatsu R.; Ohkuma T. Double Asymmetric Hydrogenation of Linear β,β-Disubstituted α,β-Unsaturated Ketones into γ-Substituted Secondary Alcohols Using a Dual Catalytic System. Chem. - Eur. J. 2017, 23 (37), 8806–8809. 10.1002/chem.201701527. 28407316

[ref40] Banerjee M.; Roy S. Rhodium(l)-Catalyzed Carbonyl Allenylation versus Propargylation via Redox Transmetalation across Tetragonal Tin(II) Oxide. Org. Lett. 2004, 6 (13), 2137–2140. 10.1021/ol0493352. 15200304

[ref41] Wang M.; Khan S.; Miliordos E.; Chen M. Enantioselective Allenylation of Aldehydes via Brønsted Acid Catalysis. Adv. Synth. Catal. 2018, 360 (23), 4634–4639. 10.1002/adsc.201801080.

[ref42] Li W.; Lin Z.; Chen L.; Tian X.; Wang Y.; Huang S. H.; Hong R. Highly Stereoselective Kinetic Resolution of α-Allenic Alcohols: An Enzymatic Approach. Tetrahedron Lett. 2016, 57 (5), 603–606. 10.1016/j.tetlet.2015.12.098.

[ref43] Miao W.; Chung L. W.; Wu Y. D.; Chan T. H. Experimental and Theoretical Studies of the Propargyl-Allenylindium System. J. Am. Chem. Soc. 2004, 126 (41), 13326–13334. 10.1021/ja049241n. 15479088

[ref44] Danheiser R. L.; Carini D. J.; Kwasigroch C. A. Scope and Stereochemical Course of the Addition of (Trimethylsilyl)Allenes to Ketones and Aldehydes. A Regiocontrolled Synthesis of Homopropargylic Alcohols. J. Org. Chem. 1986, 51 (20), 3870–3878. 10.1021/jo00370a023.

