# Supplementary material for: Photoredox Propargylation of Aldehydes Catalytic in Titanium
Source: J Org Chem. 2021 Apr 22;86(9):7002–9. doi: 10.1021/acs.joc.1c00521 (PMC8279488; doi:10.1021/acs.joc.1c00521)

Supporting Information

PHOTOREDOX PROPARGYLATION OF ALDEHYDES CATALYTIC IN TITANIUM.

Francesco Calogero,[a] Andrea Gualandi,*[a] Marco Di Matteo,[a] Simone Potenti,[a,b] Andrea Fermi,[a] Giacomo Bergamini,[a] and Pier Giorgio Cozzi*[a]

[a] Dipartimento di Chimica "Giacomo Ciamician", Alma Mater Studiorum – Università di Bologna, Via Selmi 2, 40126, Bologna, Italy

[b] Scuola Normale Superiore, Piazza dei Cavalieri 7, 56126 Pisa, Italy

E-mail: andrea.gualandi10@unibo.it; piergiorgio.cozzi@unibo.it

Table of Contents

Reaction set-up..3

Photophysical and mechanistic studies..5

Copies of NMR spectra...7

Reaction set-up

Figure S1. Reaction setup

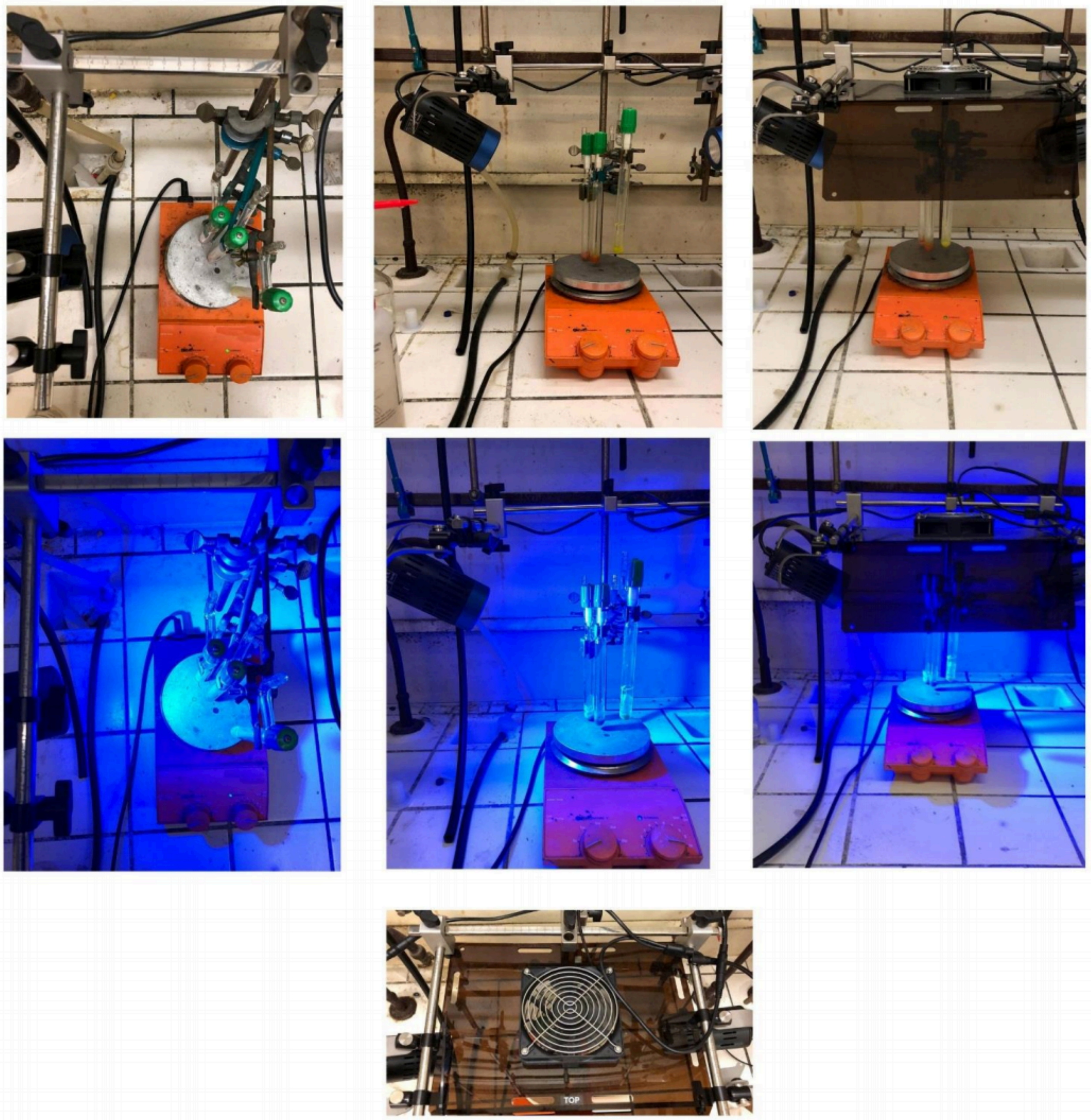

Figure S2. Reaction set-up with Kessil® PR160L@456 nm lamp for 1 mmol scale.

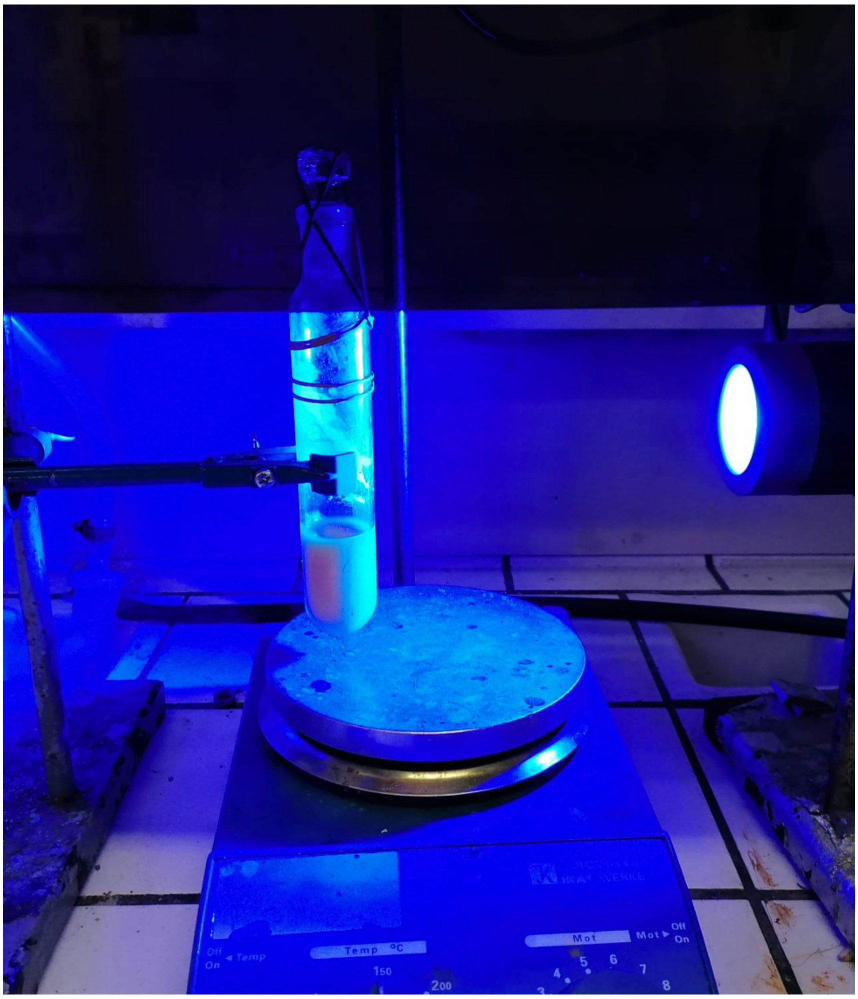

The reaction flasks were positioned approximatively at 10 cm from the light source and Kessil® PR160 Rig with Fan Kit[1] was used to control the temperature. The reaction temperature was close to room temperature during the irradiation as measured with a thermometer at 1 cm from reaction flask.

Photophysical and mechanistic studies

All the photophysical analyses were carried out in air-equilibrated tetrahydrofuran at 298 K unless otherwise specified. UV–vis absorption spectra were recorded with a PerkinElmer λ40 spectrophotometer using quartz cells with optical path length of 1.0 cm. Degassed solutions are obtained by means of repeated pump-freeze-thaw cycles (ca. $4 \cdot 10^{-6}$ mbar) in sealed quartz cuvettes. Luminescence spectra were performed with a PerkinElmer LS-50, a Varian Cary Eclipse or an Edinburgh FLS920 spectrofluorimeter equipped with a Hamamatsu R928 phototube. Lifetimes shorter than 10 µs were measured by the above-mentioned Edinburgh FLS920 spectrofluorimeter equipped with a TCC900 card for data acquisition in time-correlated single-photon counting experiments (0.5 ns time resolution). The estimated experimental errors are 2 nm on the band maximum, 5% on the molar absorption coefficient and luminescence lifetime.

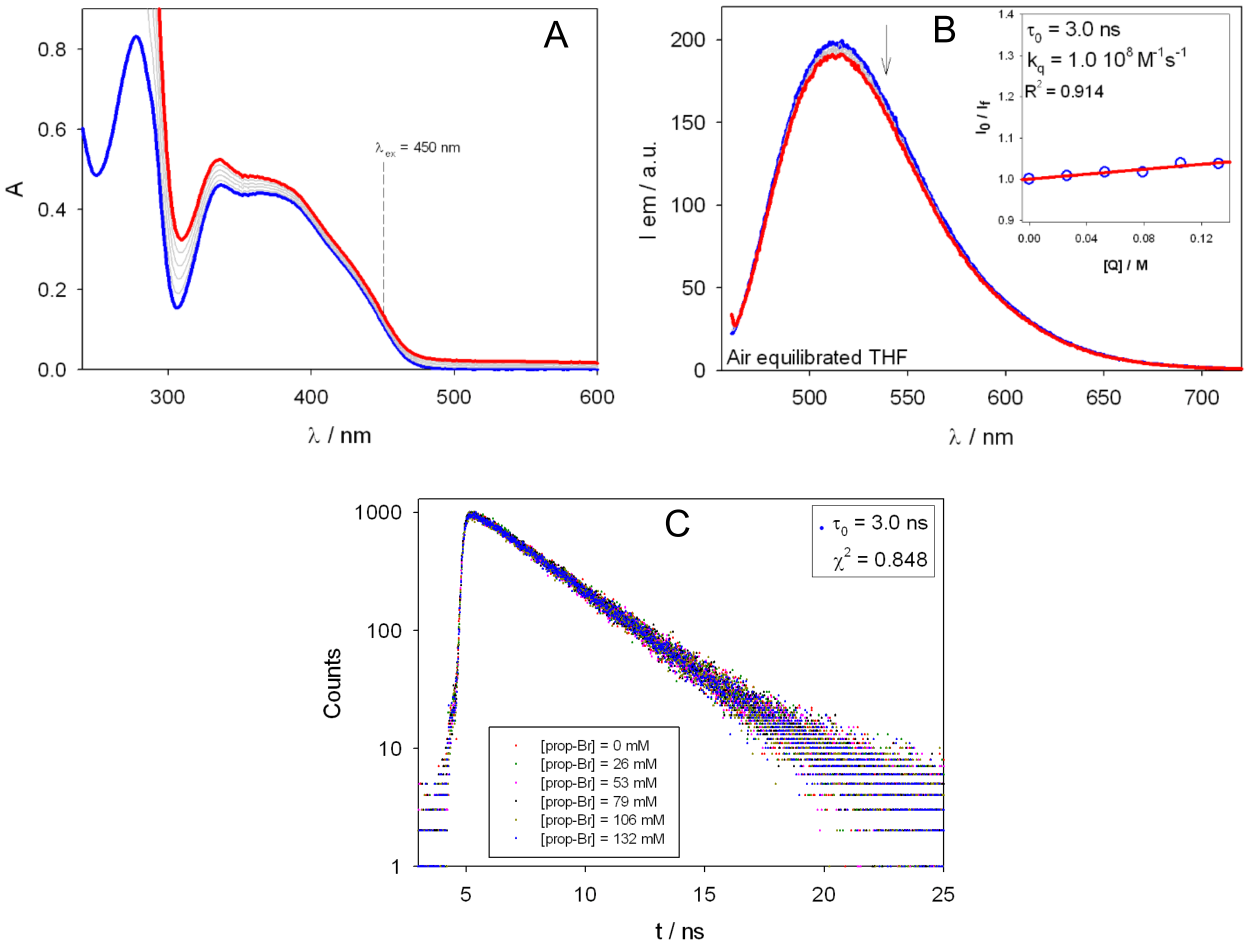

Figure S3. A: absorption spectra of solutions of **3-DPAFIPN** in air-equilibrated THF (blue line) obtained upon addition of increasing amounts of propargyl bromide (**prop-Br**, up to ca. 0.13 M, red line). **B**: fluorescence spectra of **3-DPAFIPN** obtained from the same solutions at λ_{ex}= 450 nm. Inset: Stern-Volmer diagram relative to the fluorescence spectra shown in B. **C**: fluorescence decays of **3-DPAFIPN** obtained from the same solutions as B, at λ_{em}=510 nm (λ_{ex}= 405 nm). Since the fluorescence lifetime of **3-DPAFIPN** is not significantly changing upon increasing additions of propargyl bromide, no Stern-Volmer constant has been calculated.

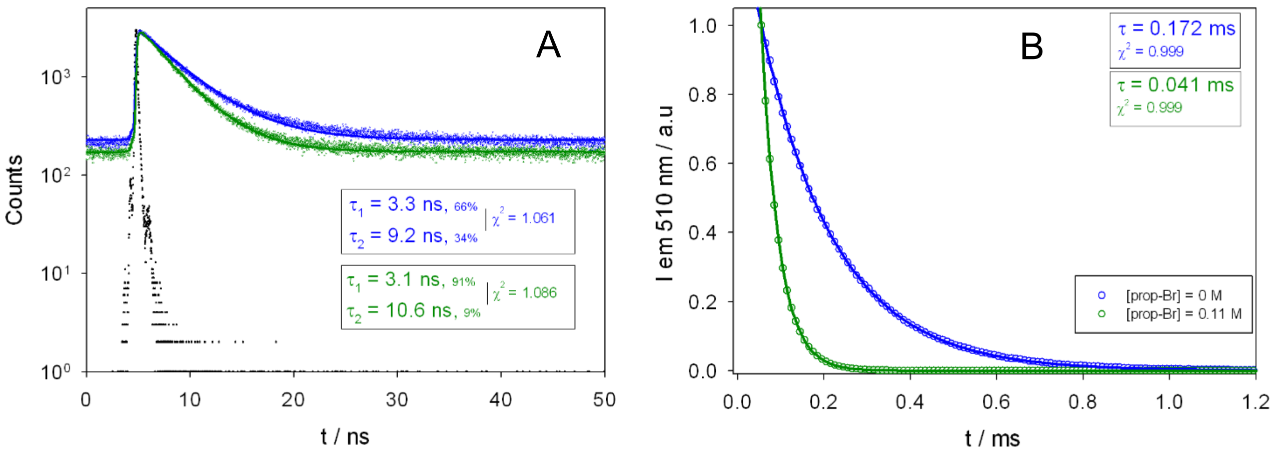

Figure S4. A: prompt fluorescence decays of **3-DPAFIPN** obtained from degassed THF solutions at λ_{em}=510 nm (pump-freeze-thaw, 3 cycles; λ_{ex}= 405 nm) in the absence and in the presence of **prop-Br** 0.11 M (blue and green dots, respectively). The corresponding biexponential fitting curves are shown as the blue and green solid lines. The instrument response function is also shown (black dots). **B**: normalised emission decays of **3-DPAFIPN** in degassed THF solutions (pump-freeze-thaw, 3 cycles) in the absence (blue dots) and in the presence of **prop-Br** 0.11 M (green dots). The decays are fitted with monoexponential functions (blue and green solid lines, respectively). λ_{ex}=350 nm, λ_{em}=510 nm; delay=0.05 ms; gate=0.01 ms. Based on these emission lifetimes, the quenching constant k_q is estimated as ca. $1.8{\bullet}10^5$ $M^{-1}s^{-1}$.

Copies of NMR spectra

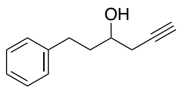

3a ^{1}H NMR (401 MHz, $CDCl_3$)

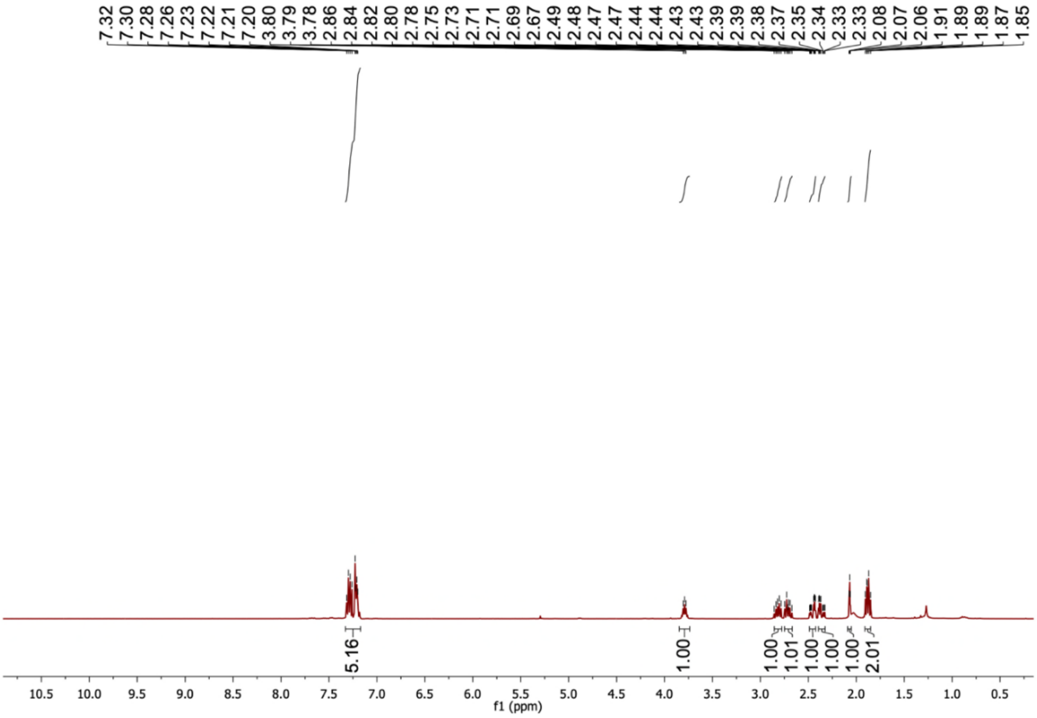

$^{13}C\{^{1}H\}$ NMR (101 MHz, $CDCl_3$)

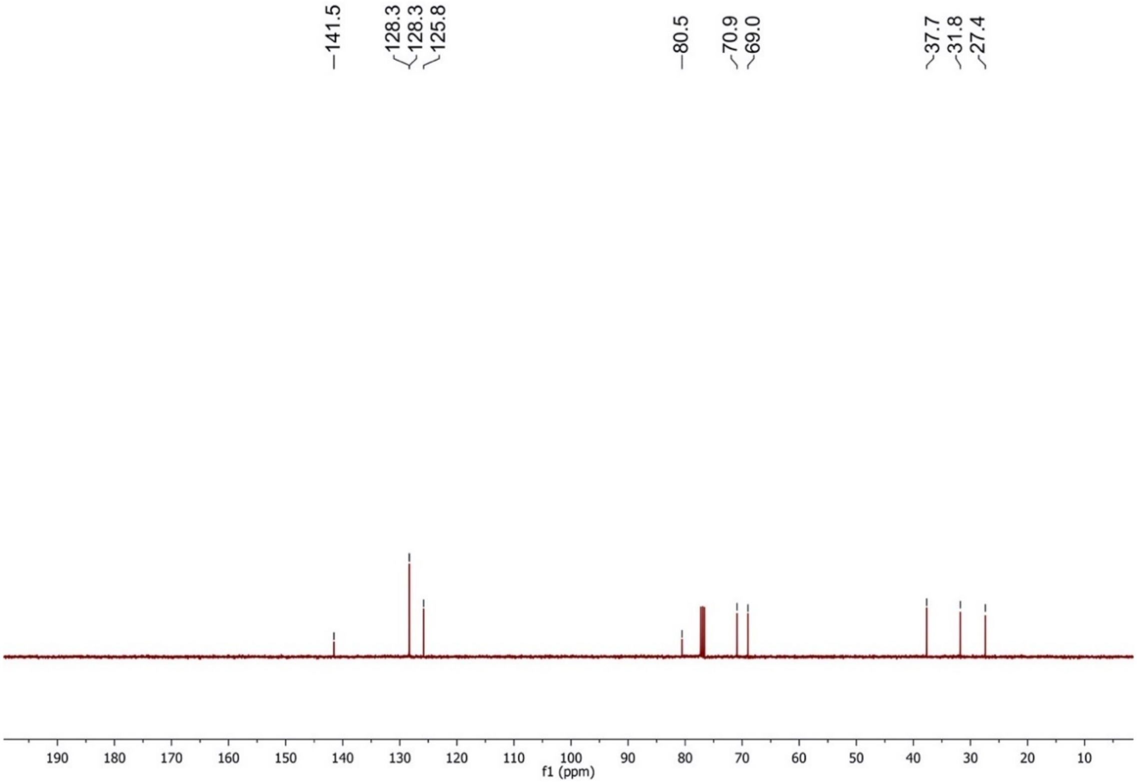

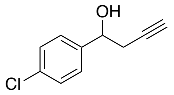

3b ^{1}H NMR (401 MHz, $CDCl_3$)

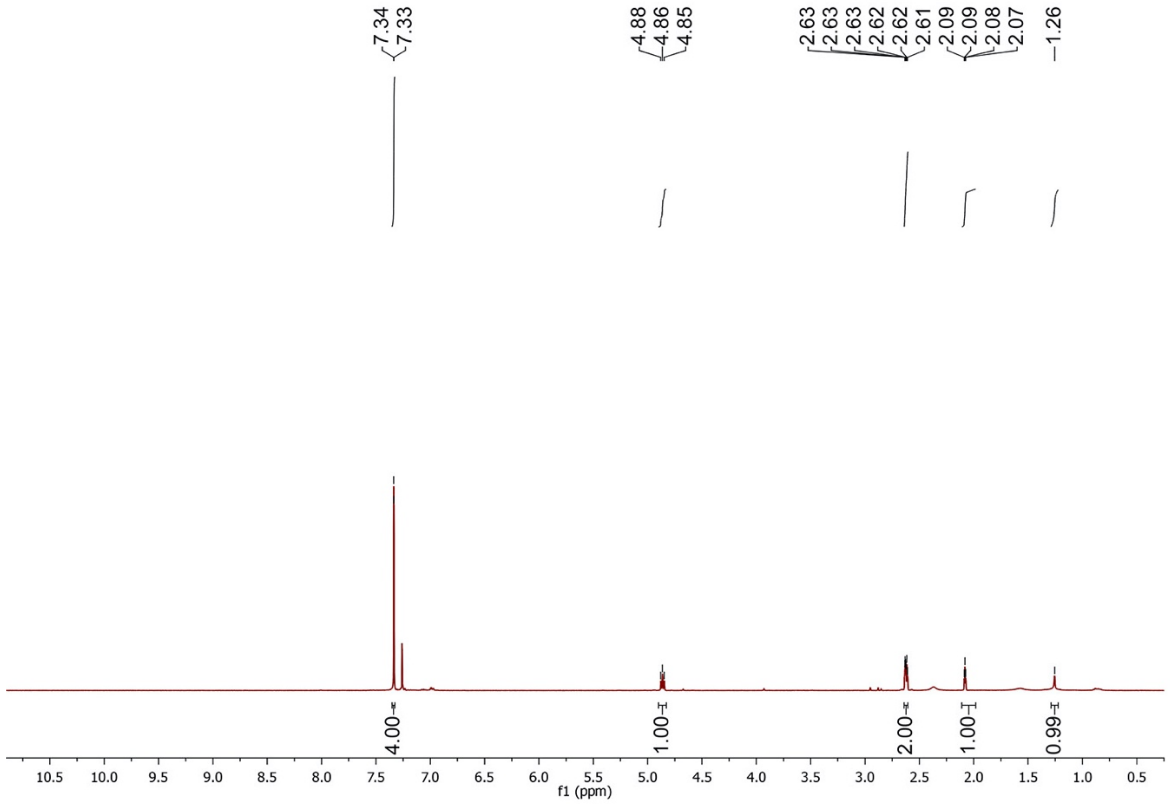

^{13}C{^{1}H} NMR (101 MHz, $CDCl_3$)

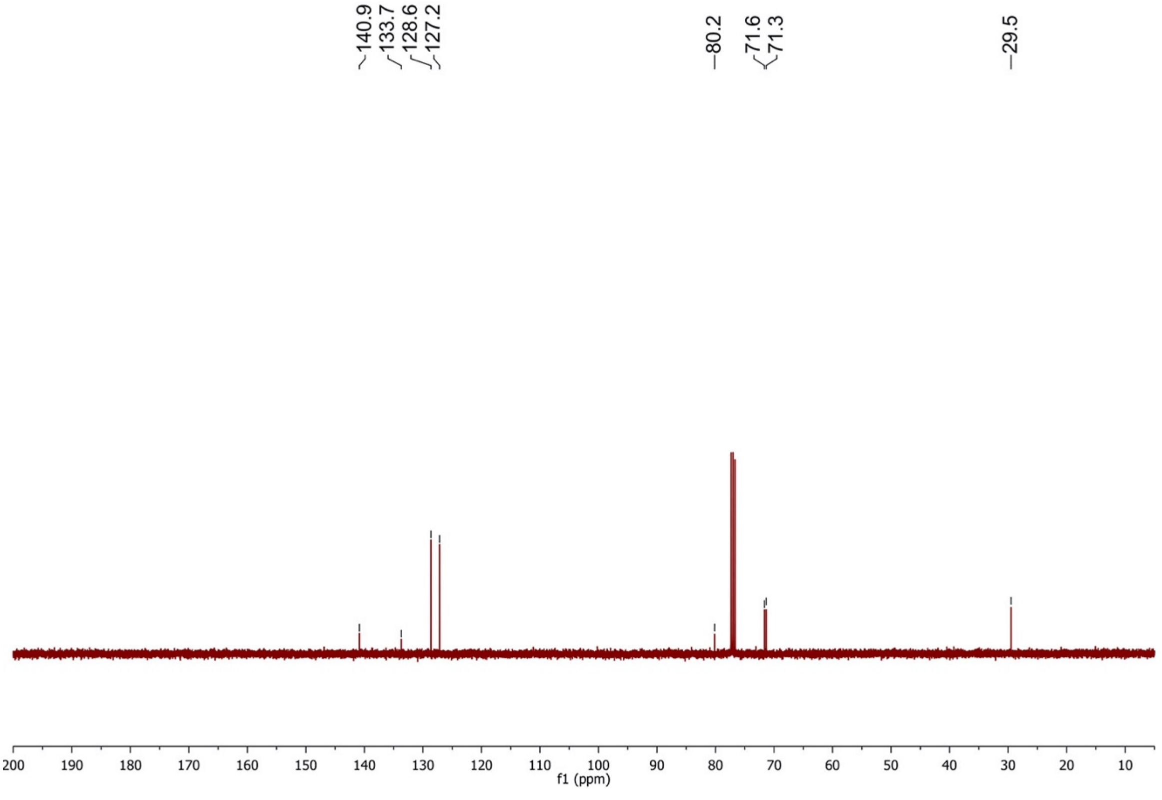

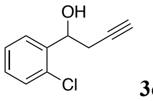

3c ^{1}H NMR (401 MHz, $CDCl_3$)

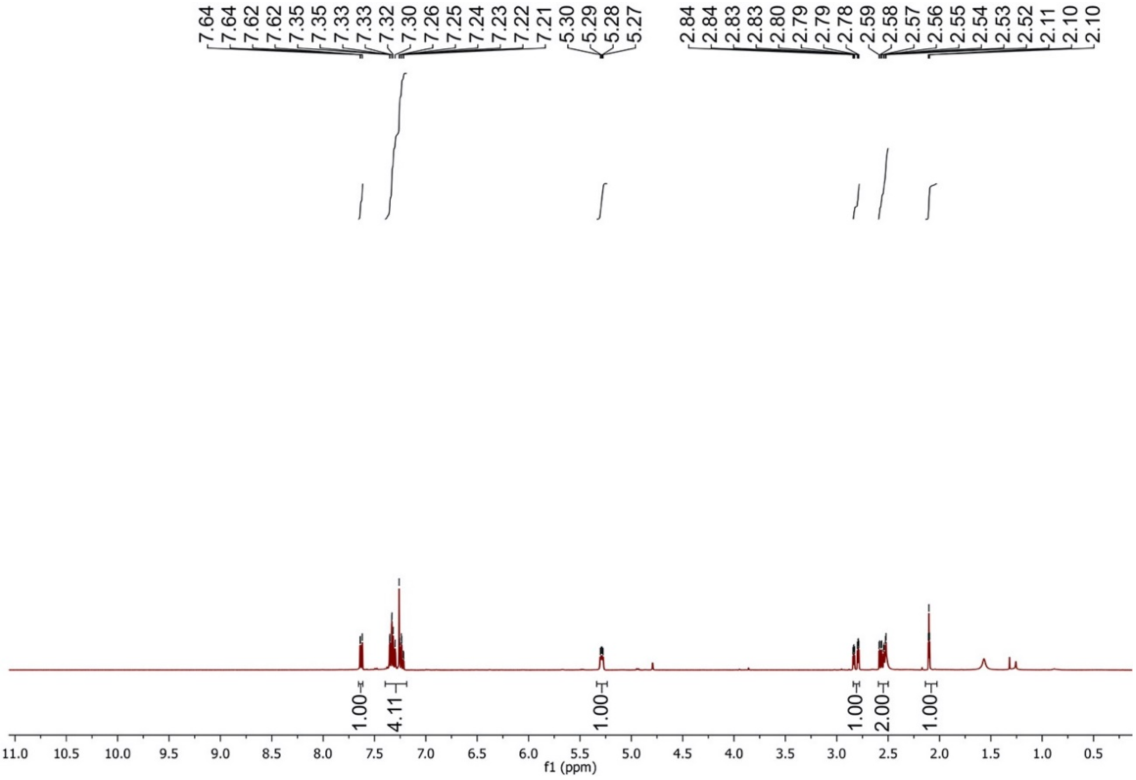

^{13}C{^{1}H} NMR (101 MHz, $CDCl_3$)

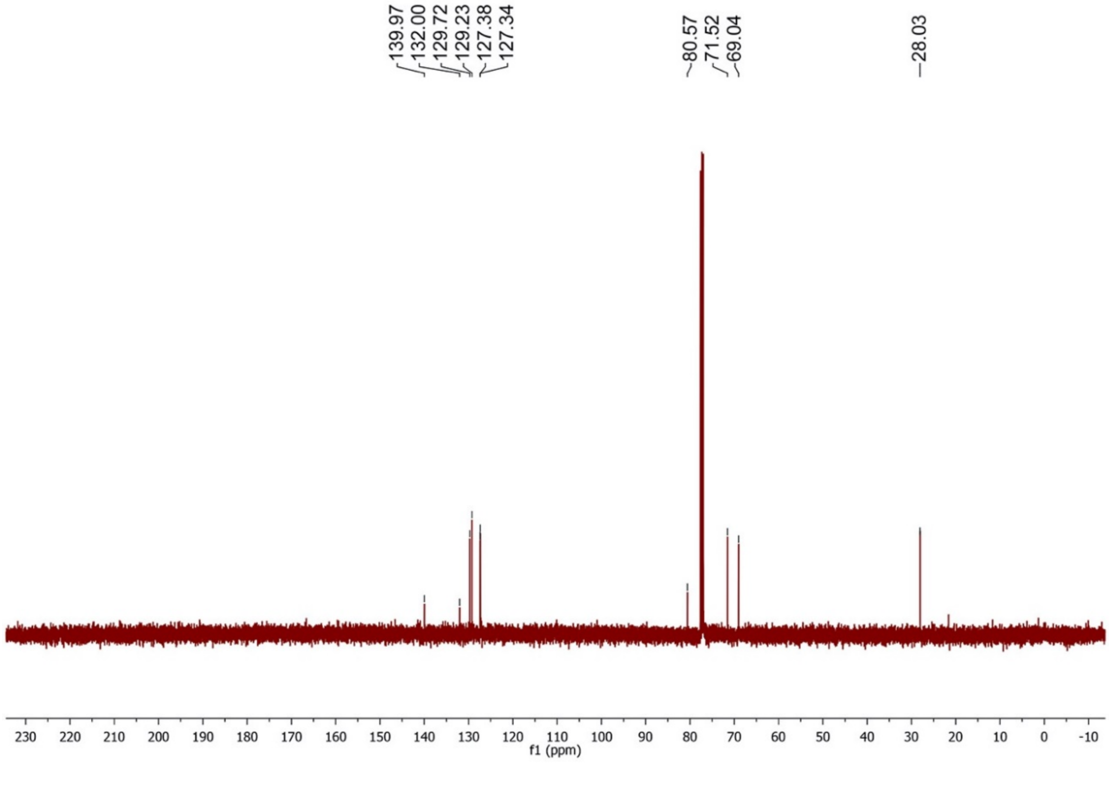

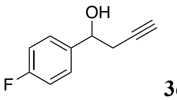

3d ^{1}H NMR (401 MHz, $CDCl_3$)

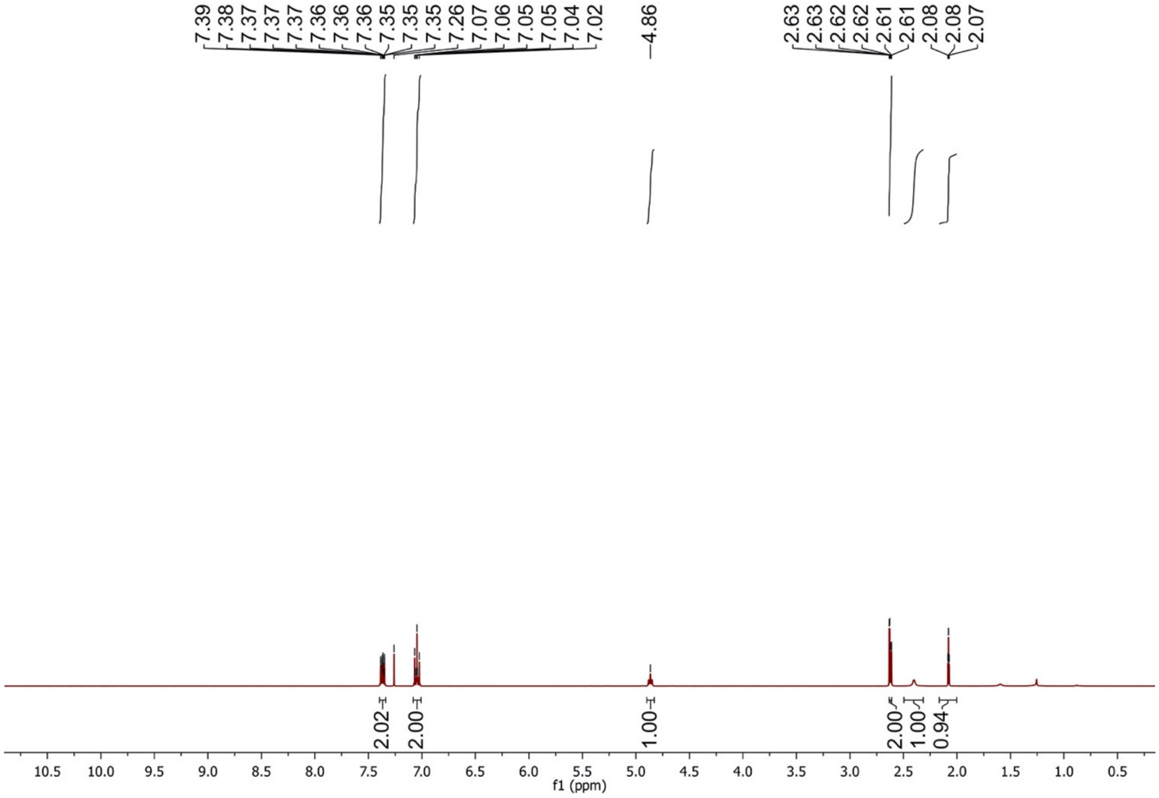

$^{13}C\{^{1}H\}$ NMR (101 MHz, $CDCl_3$)

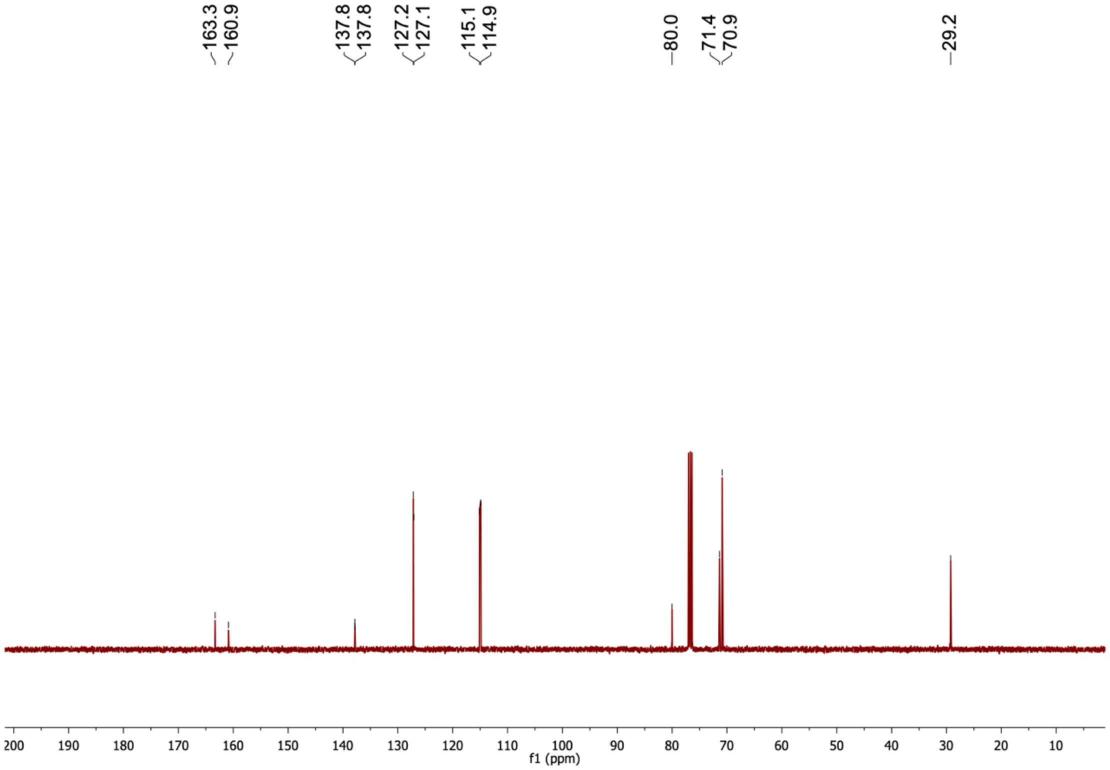

^{19}F NMR (376.5 Hz, $CDCl_3$)

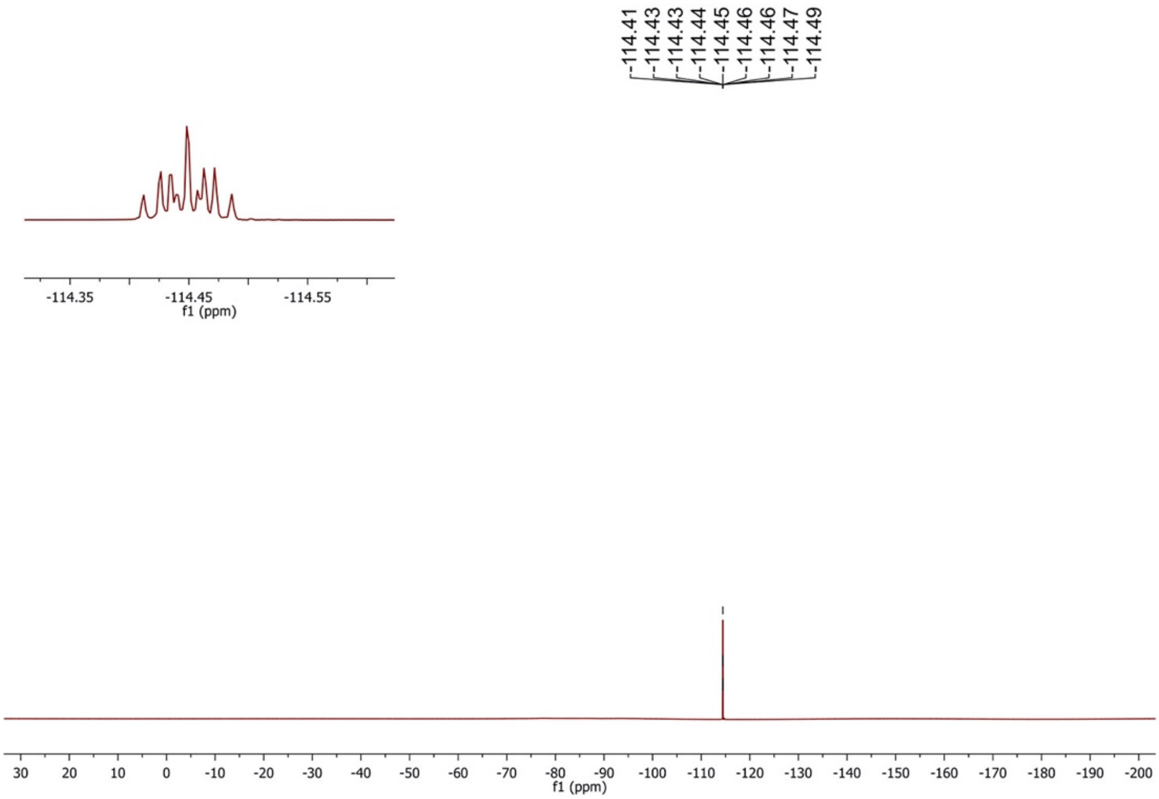

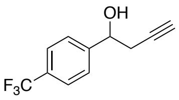

3e ^{1}H NMR (401 MHz, $CDCl_3$)

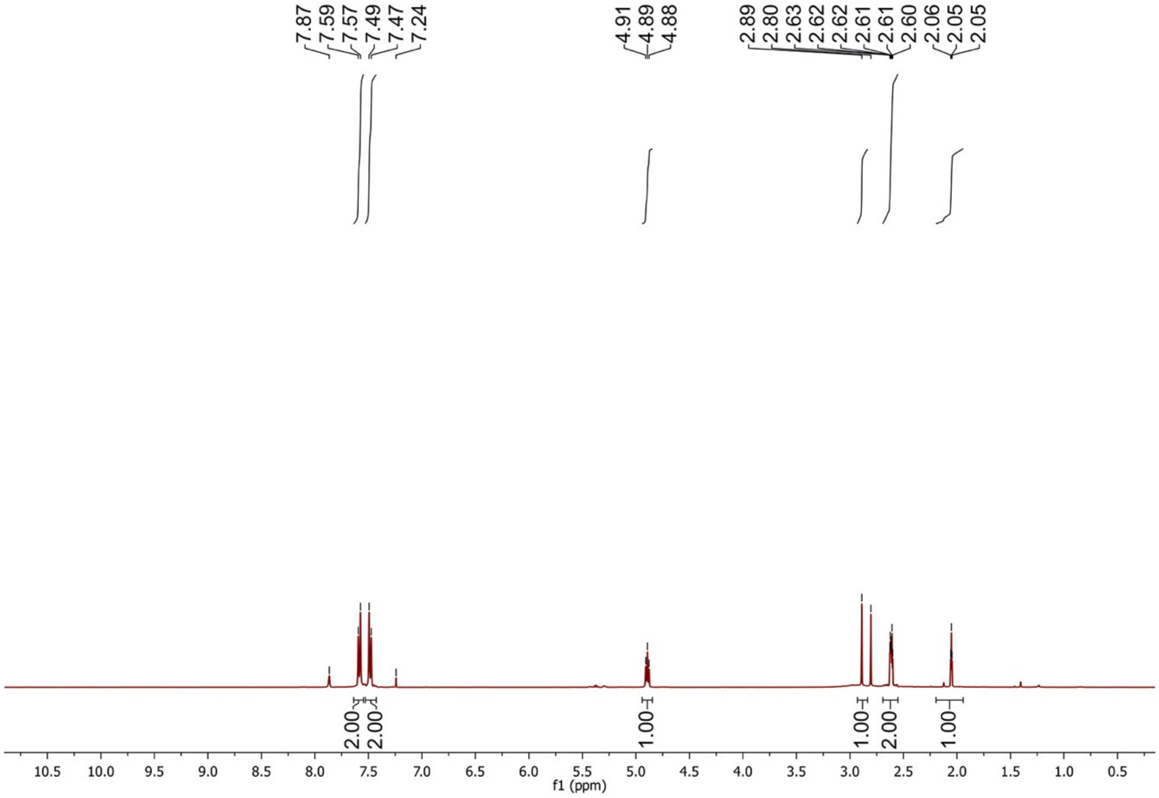

^{13}C{^{1}H} NMR (101 MHz, $CDCl_3$)

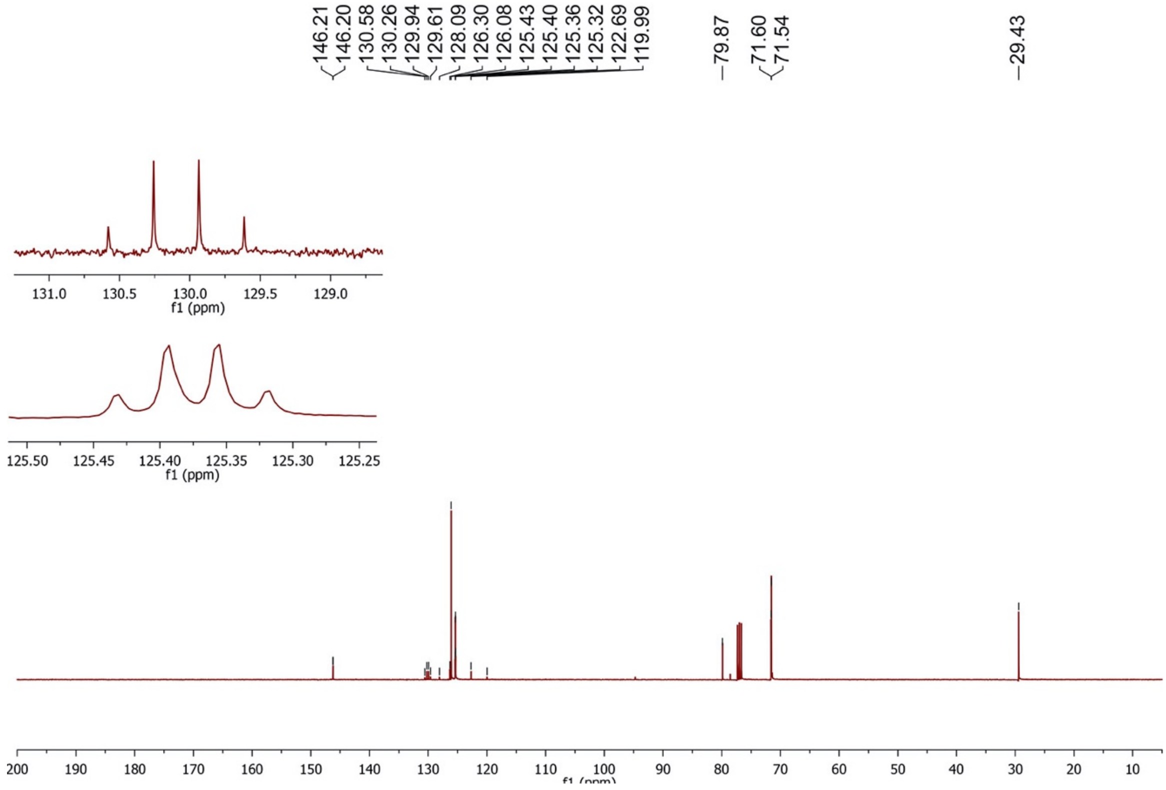

^{19}F NMR (376.5 Hz, $CDCl_3$)

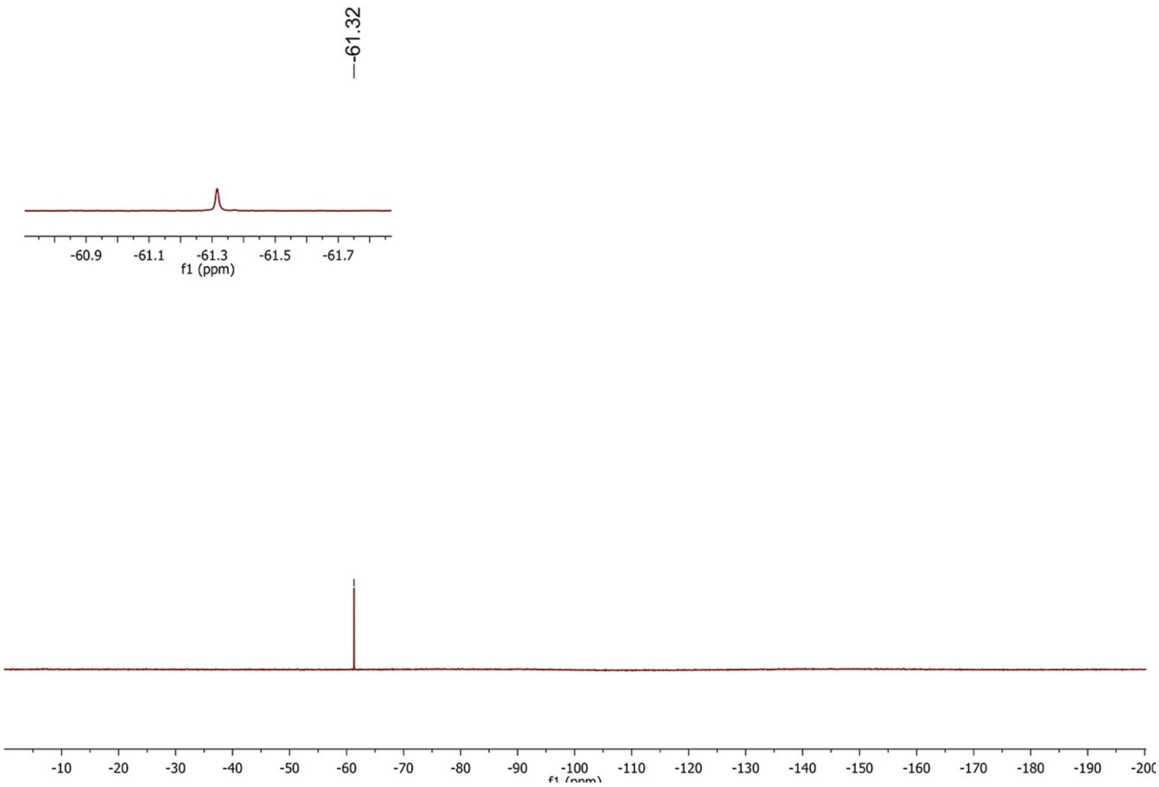

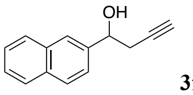

3f ^{1}H NMR (401 MHz, $CDCl_3$)

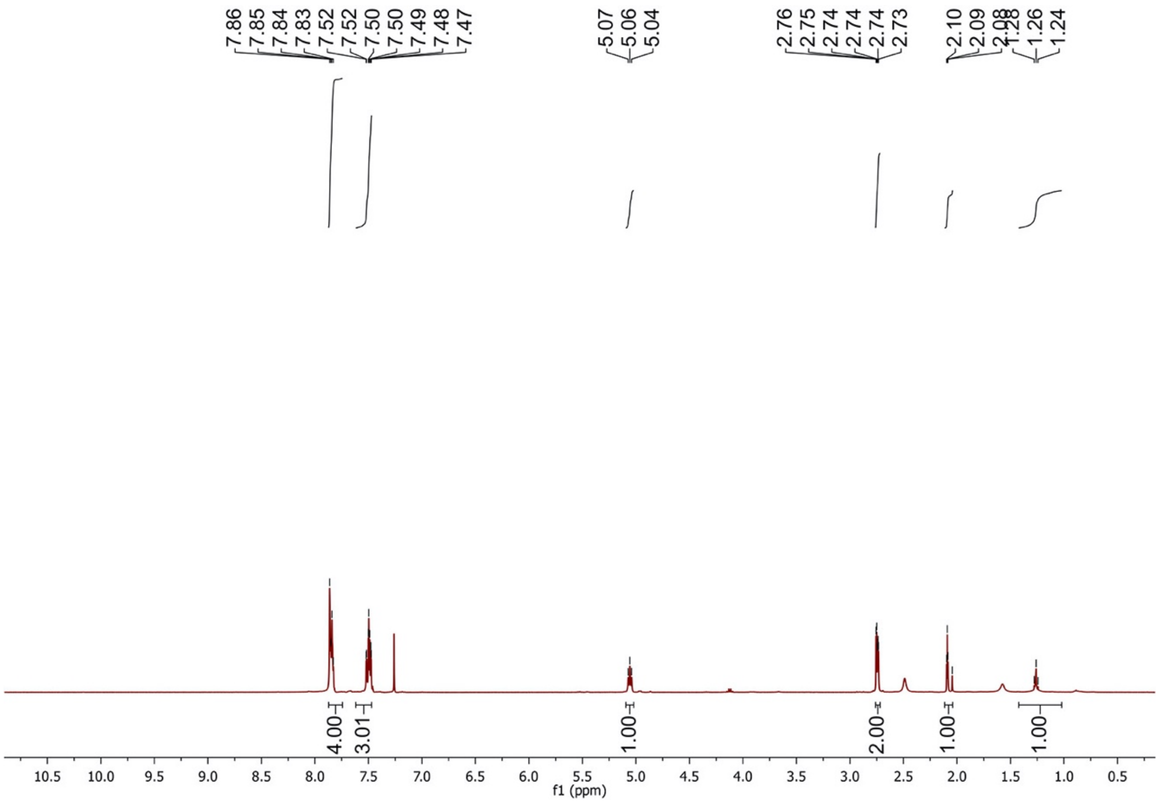

$^{13}C\{^{1}H\}$ NMR (101 MHz, $CDCl_3$)

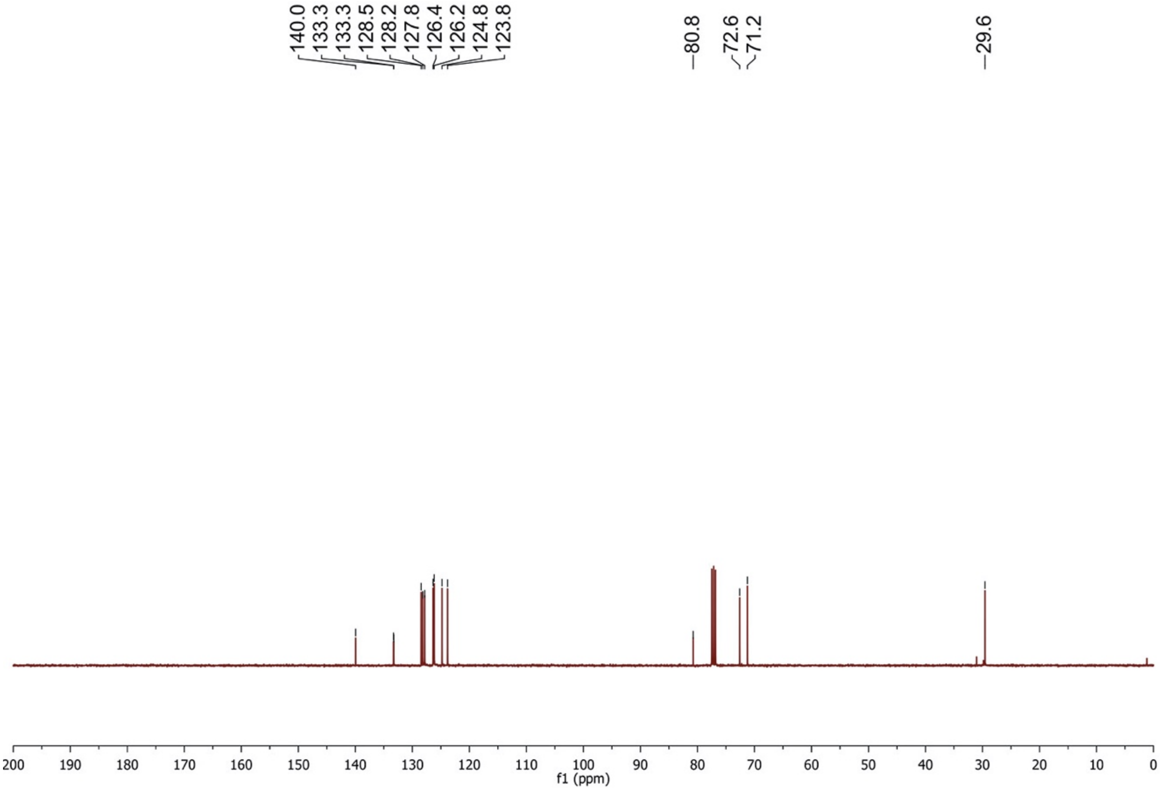

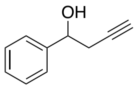

3g ^{1}H NMR (401 MHz, $CDCl_3$)

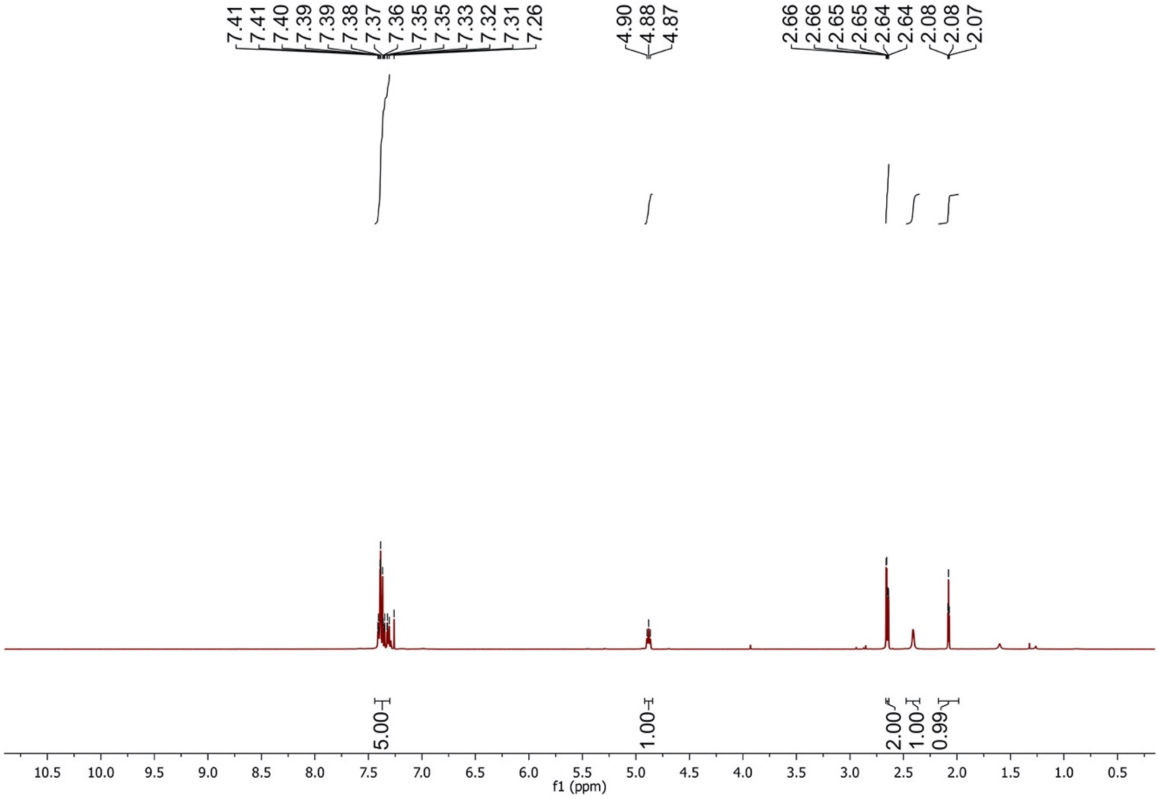

$^{13}C\{^{1}H\}$ NMR (101 MHz, $CDCl_3$)

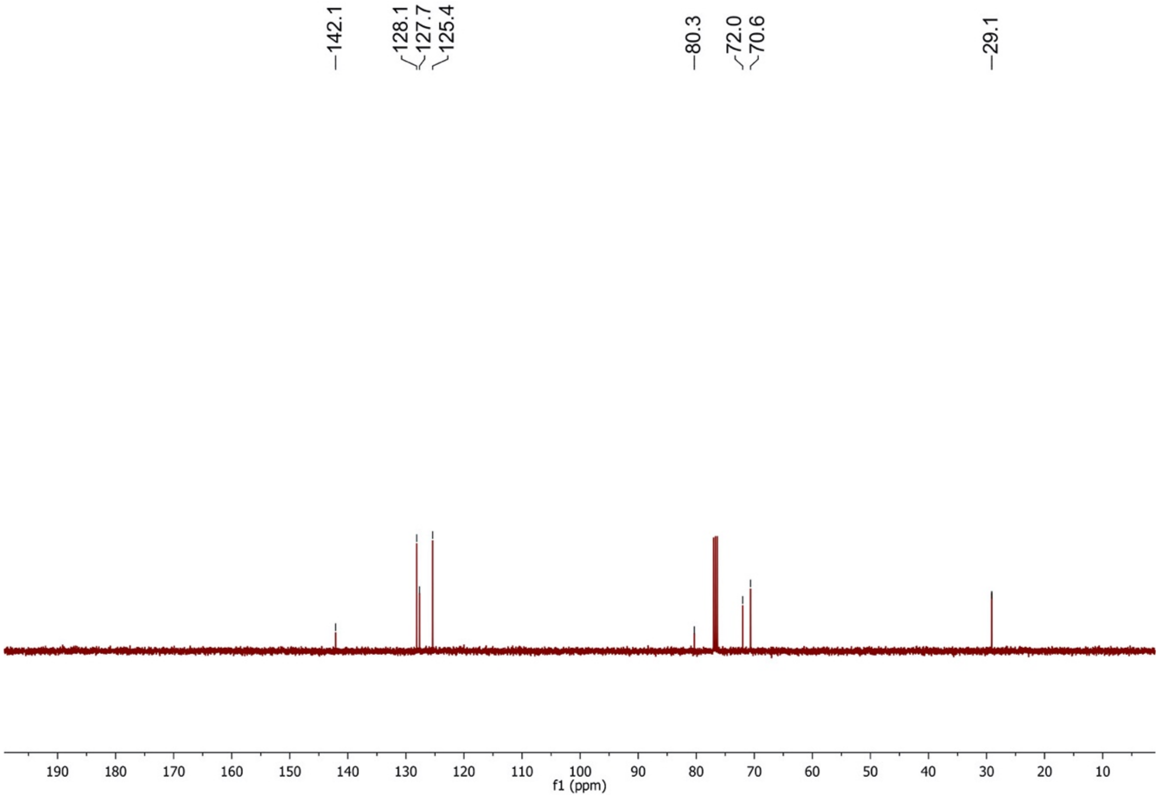

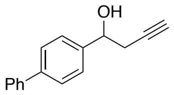

3h ^{1}H NMR (401 MHz, $CDCl_3$)

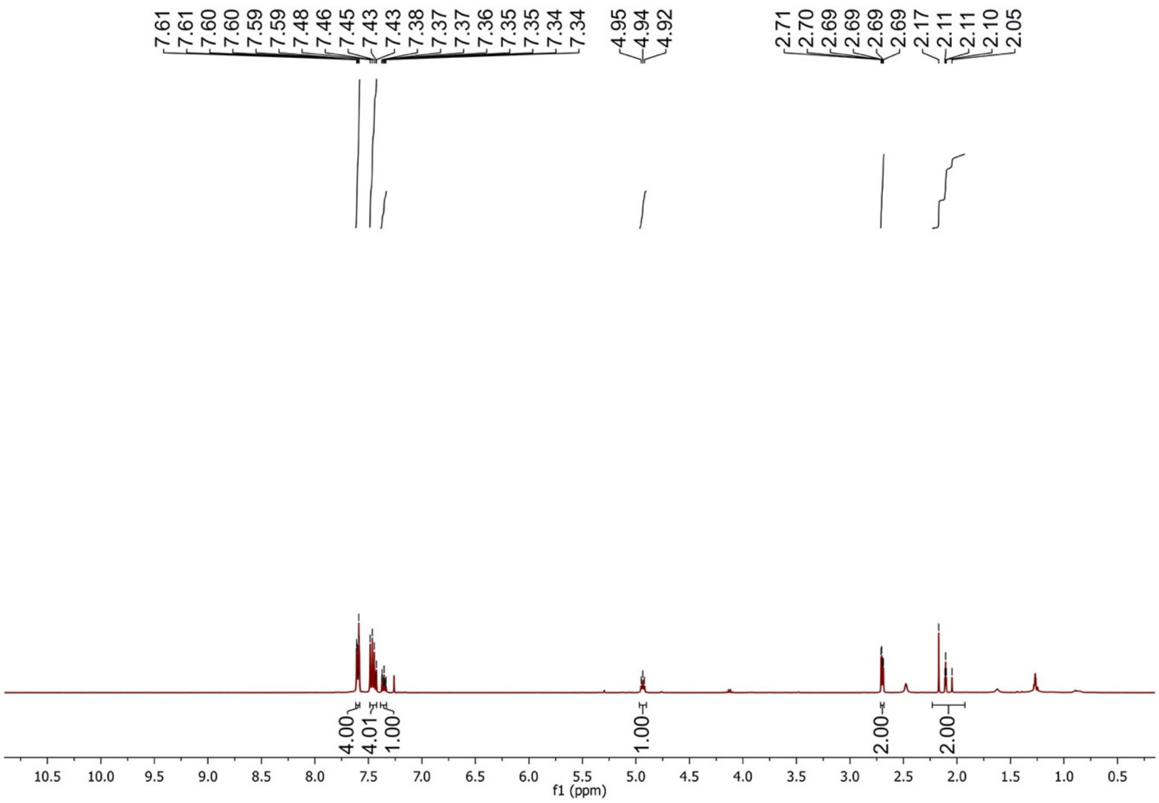

$^{13}C\{^{1}H\}$ NMR (101 MHz, $CDCl_3$)

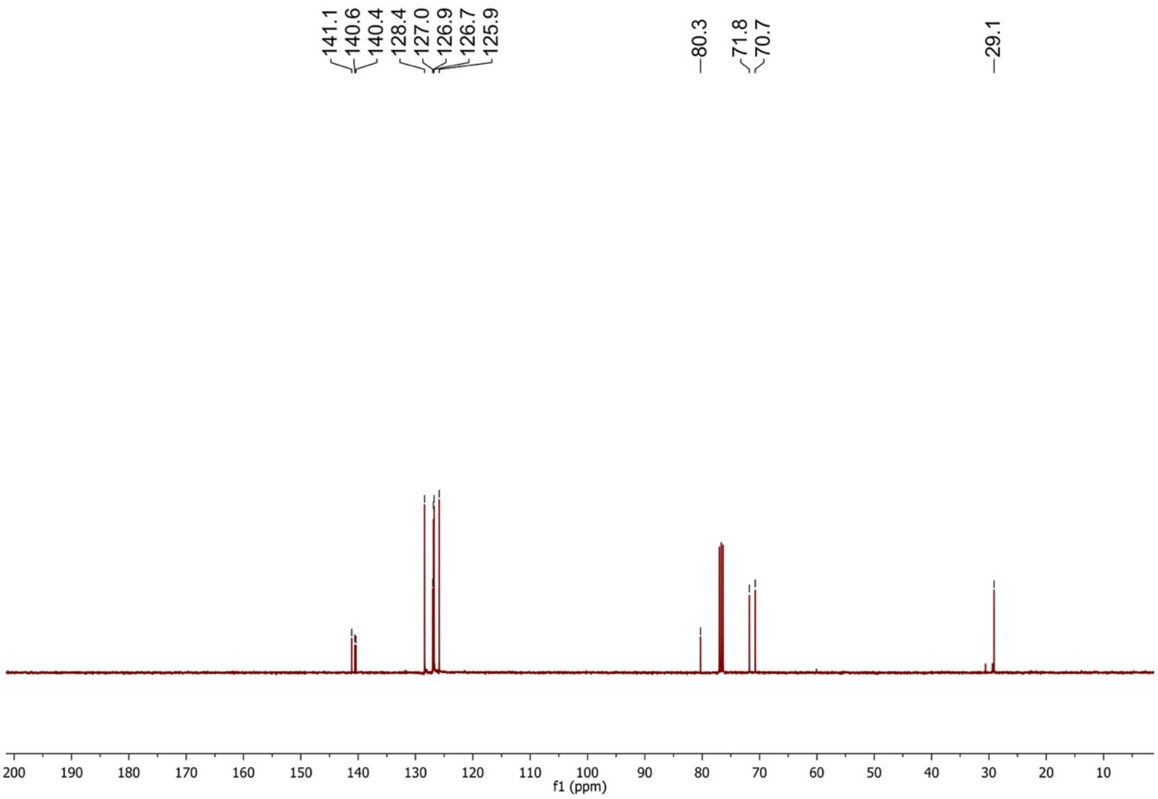

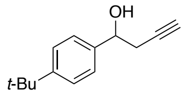

3i ^{1}H NMR (401 MHz, $CDCl_3$)

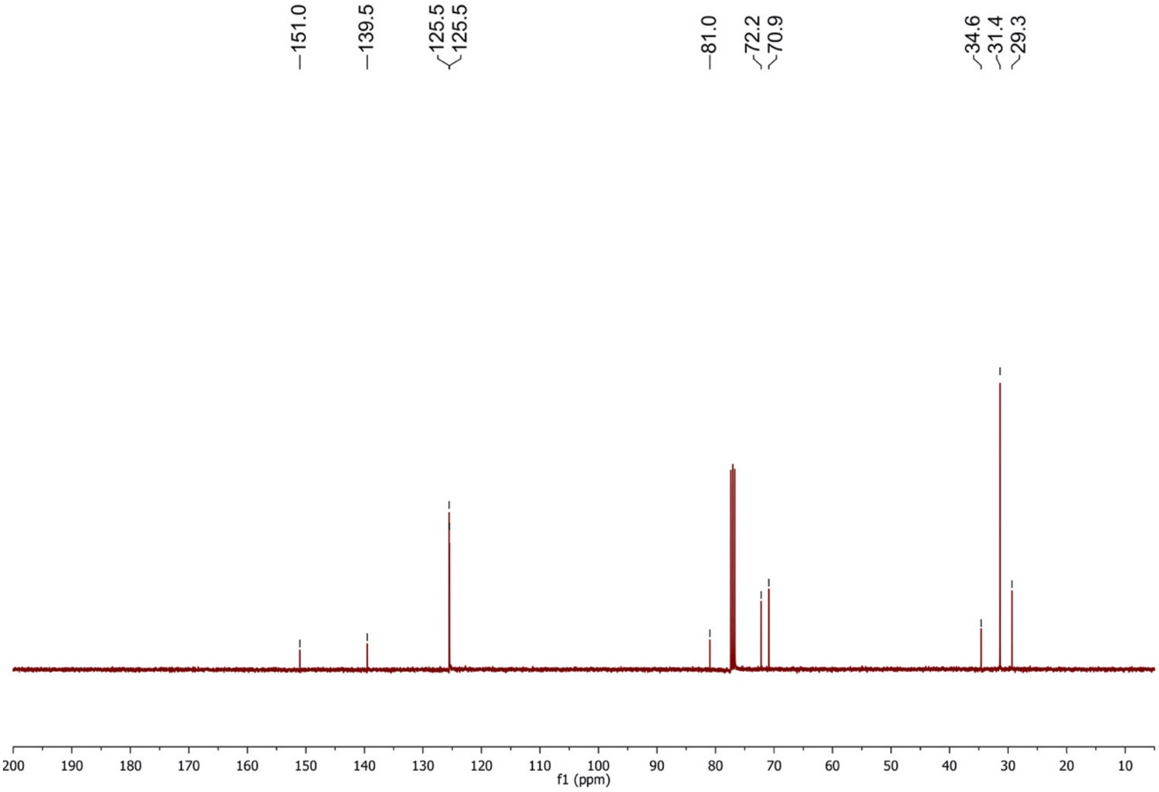

$^{13}C\{^{1}H\}$ NMR (101 MHz, $CDCl_3$)

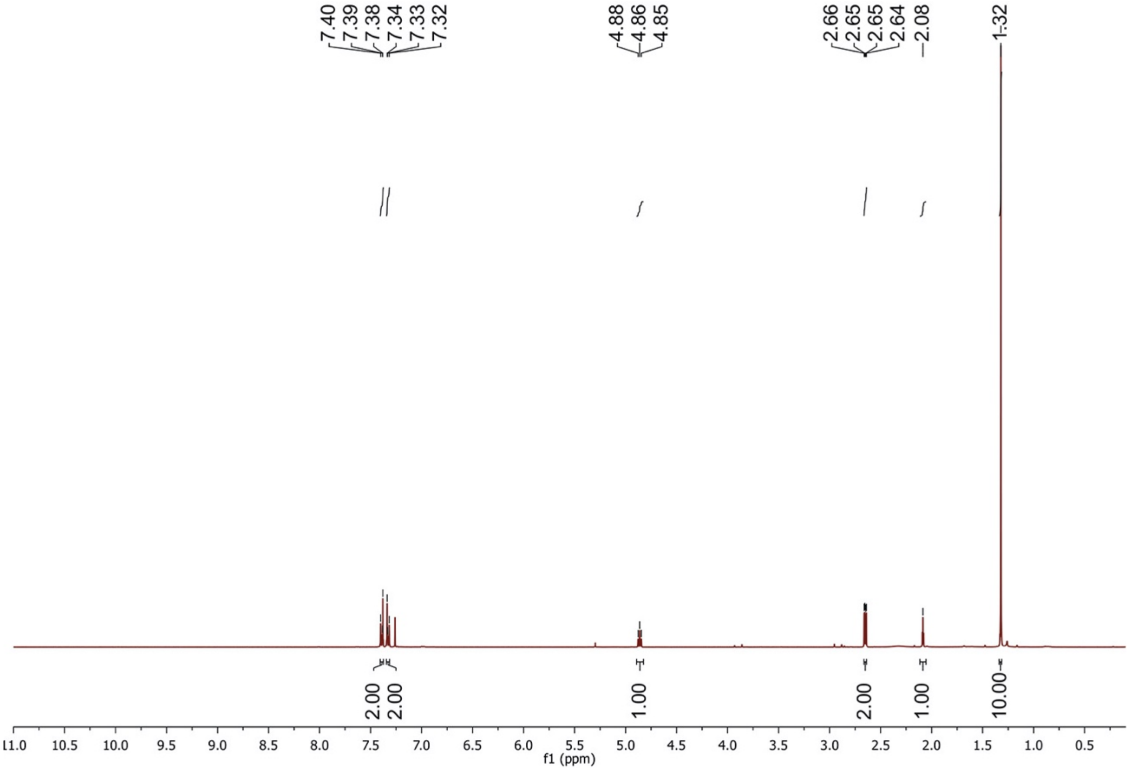

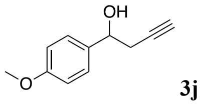

3j ^{1}H NMR (401 MHz, $CDCl_3$)

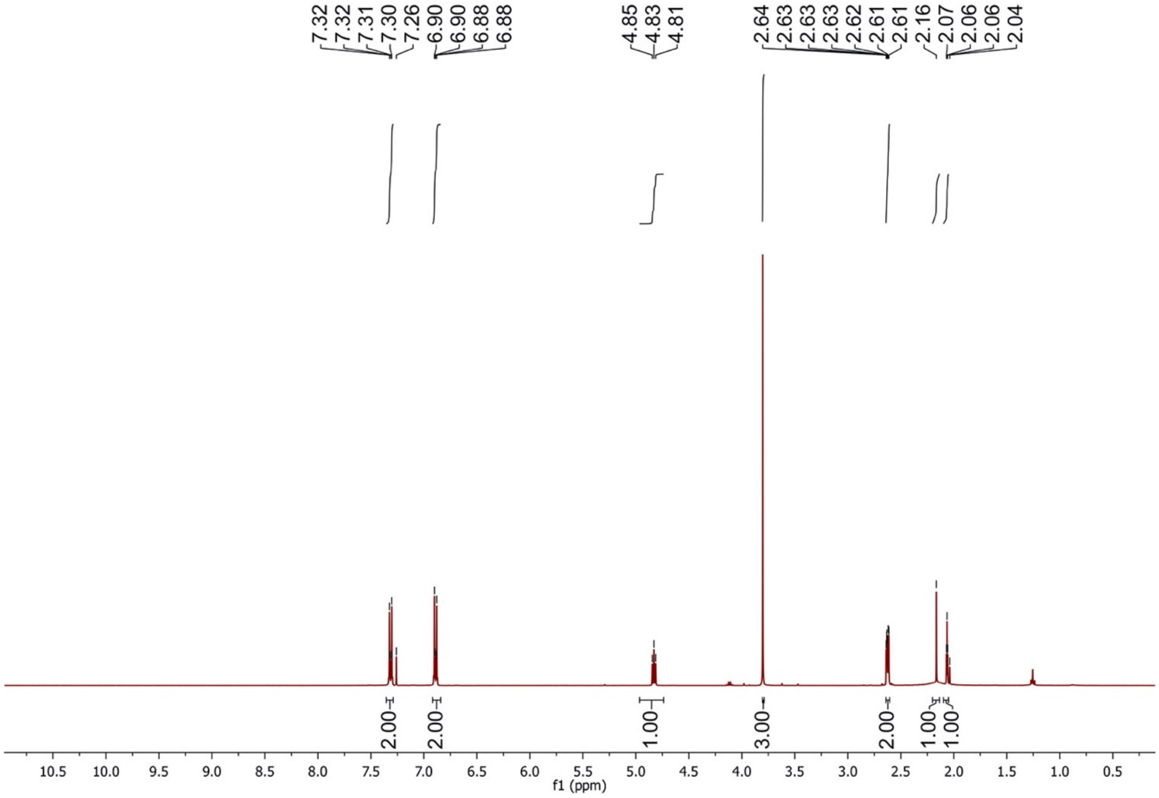

$^{13}C\{^{1}H\}$ NMR (101 MHz, $CDCl_3$)

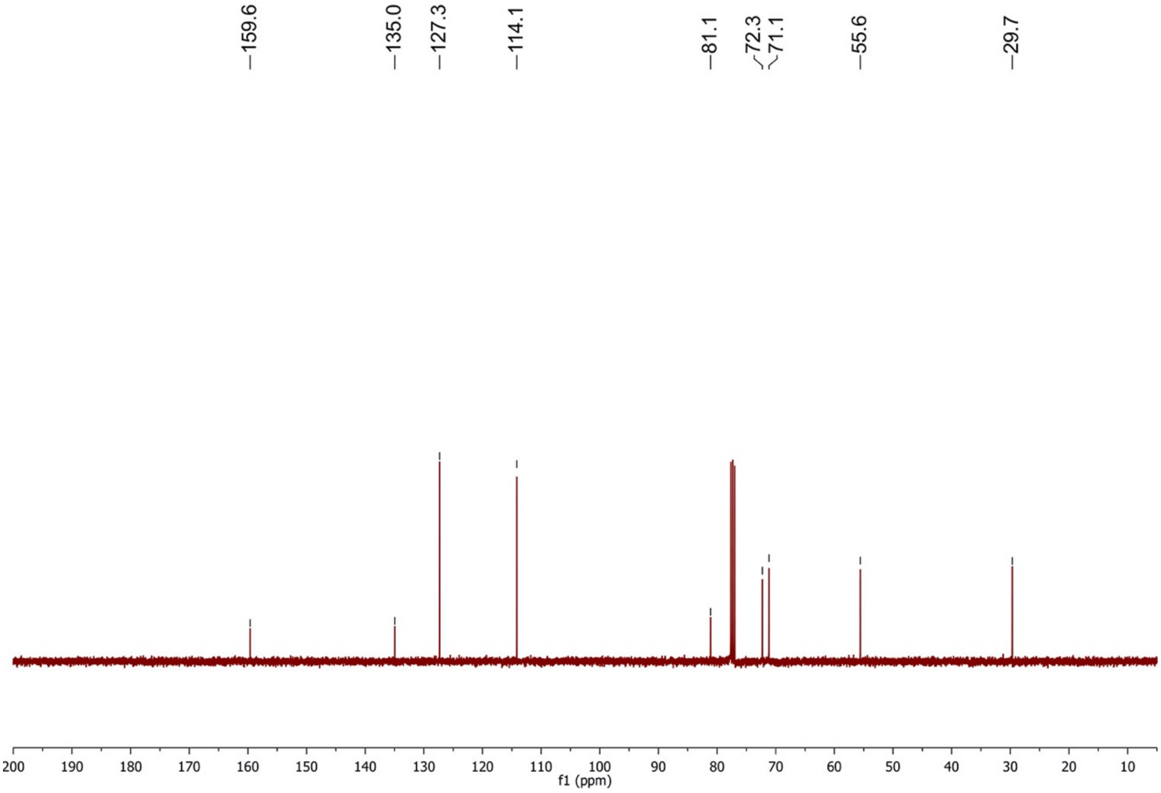

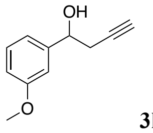

3k ^{1}H NMR (401 MHz, $CDCl_3$)

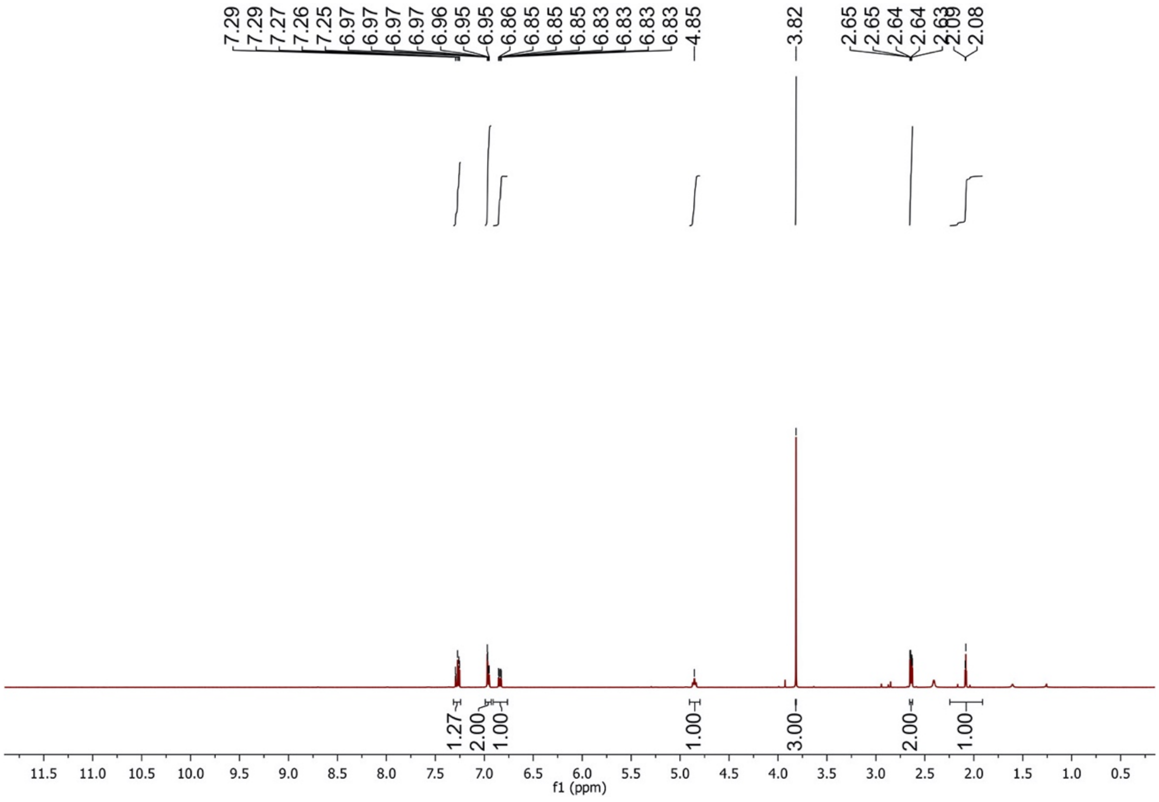

$^{13}C\{^{1}H\}$ NMR (101 MHz, $CDCl_3$)

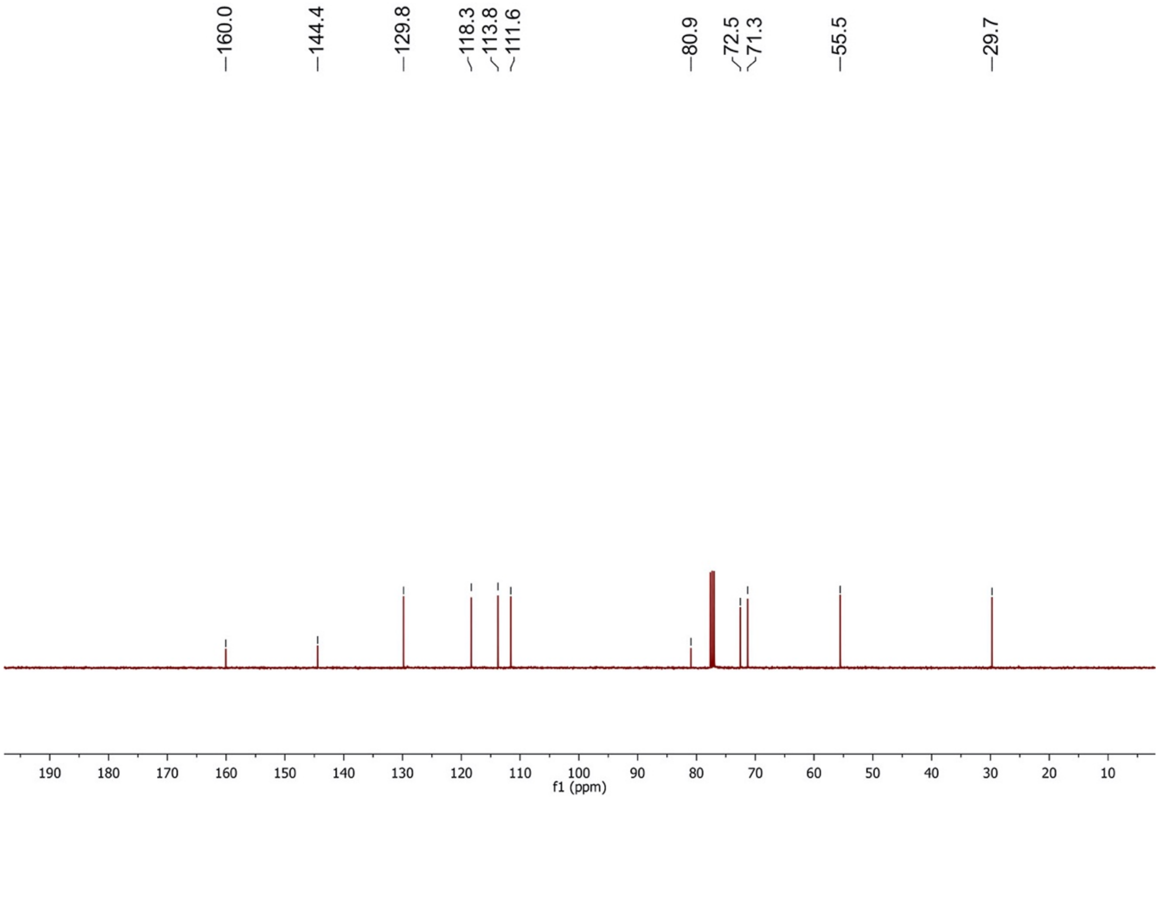

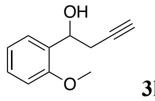

3l ^{1}H NMR (401 MHz, $CDCl_3$)

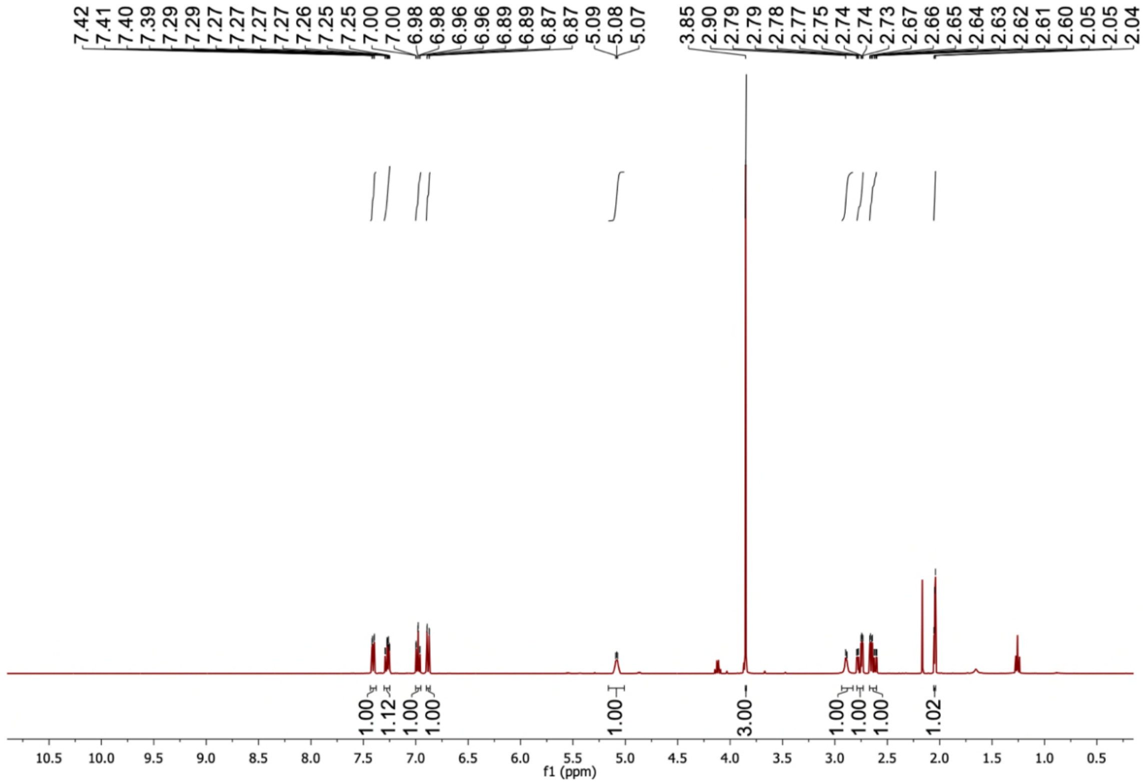

$^{13}C\{^{1}H\}$ NMR (101 MHz, $CDCl_3$)

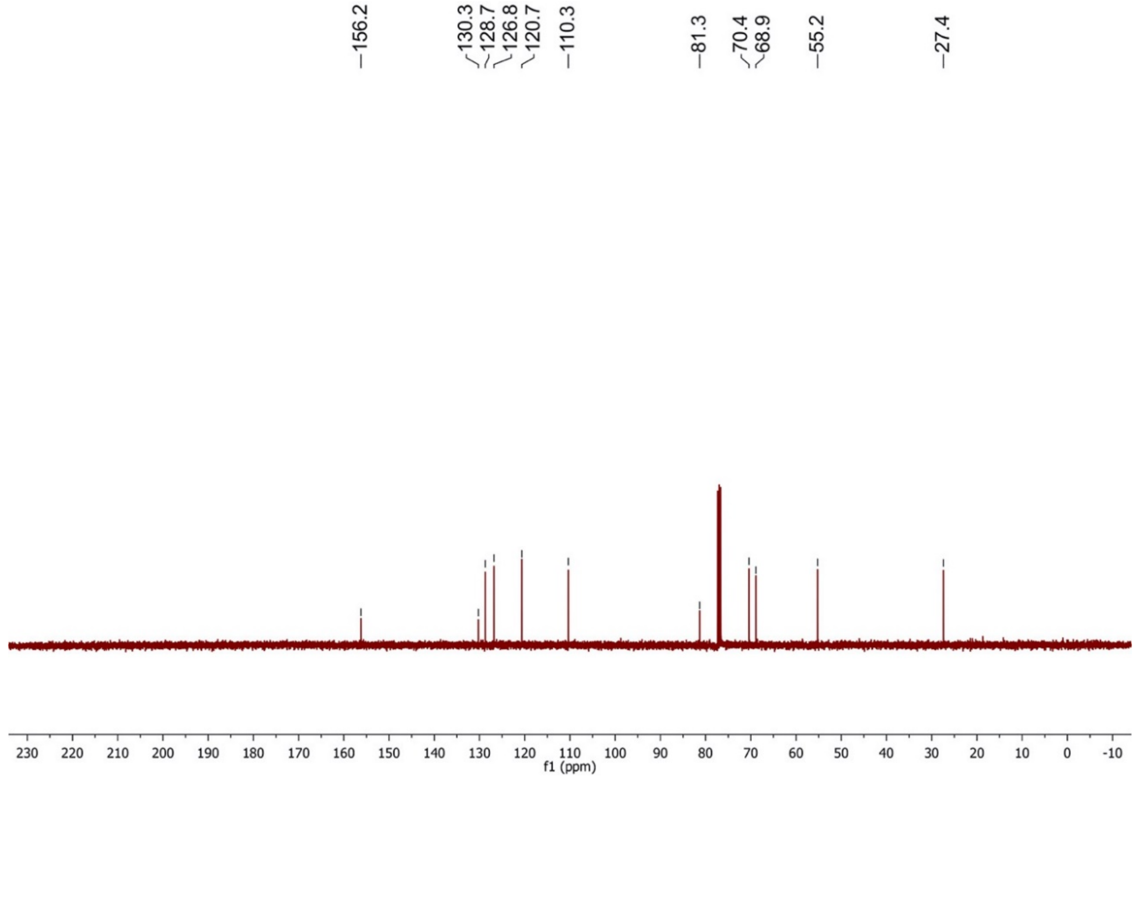

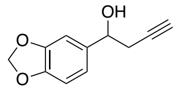

3m ^{1}H NMR (401 MHz, $CDCl_3$)

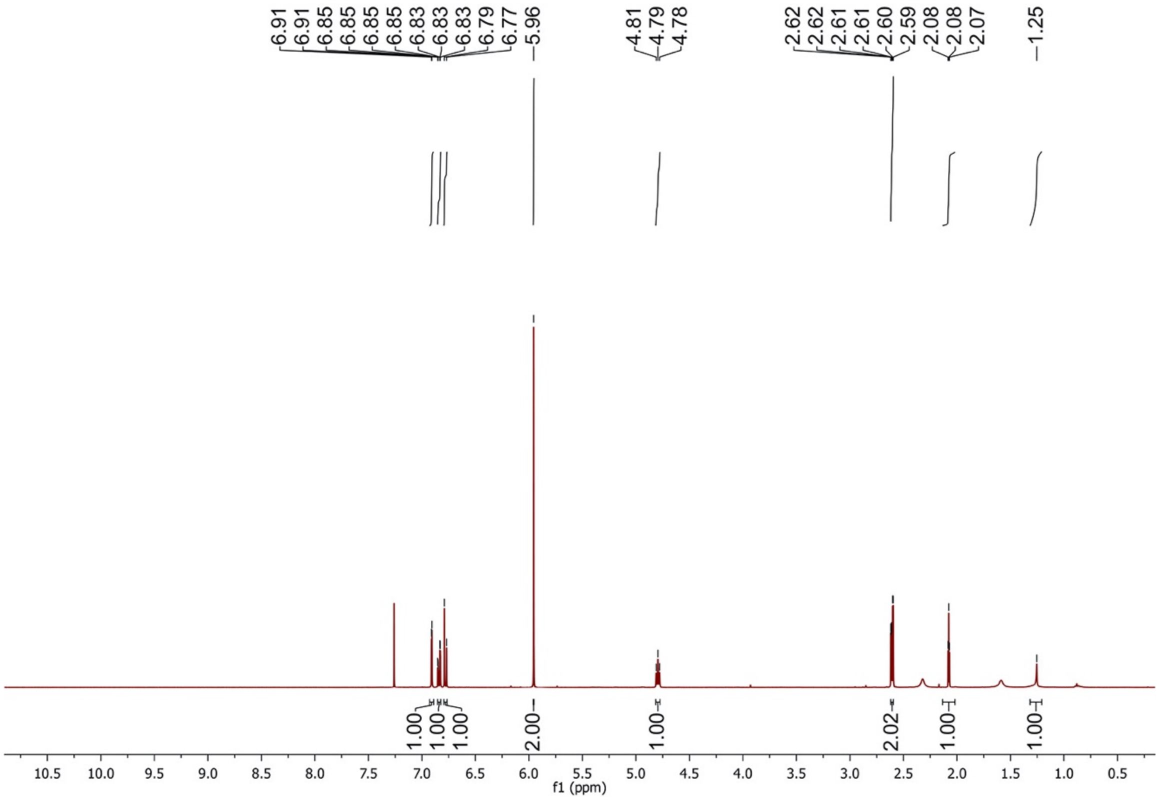

$^{13}C\{^{1}H\}$ NMR (101 MHz, $CDCl_3$)

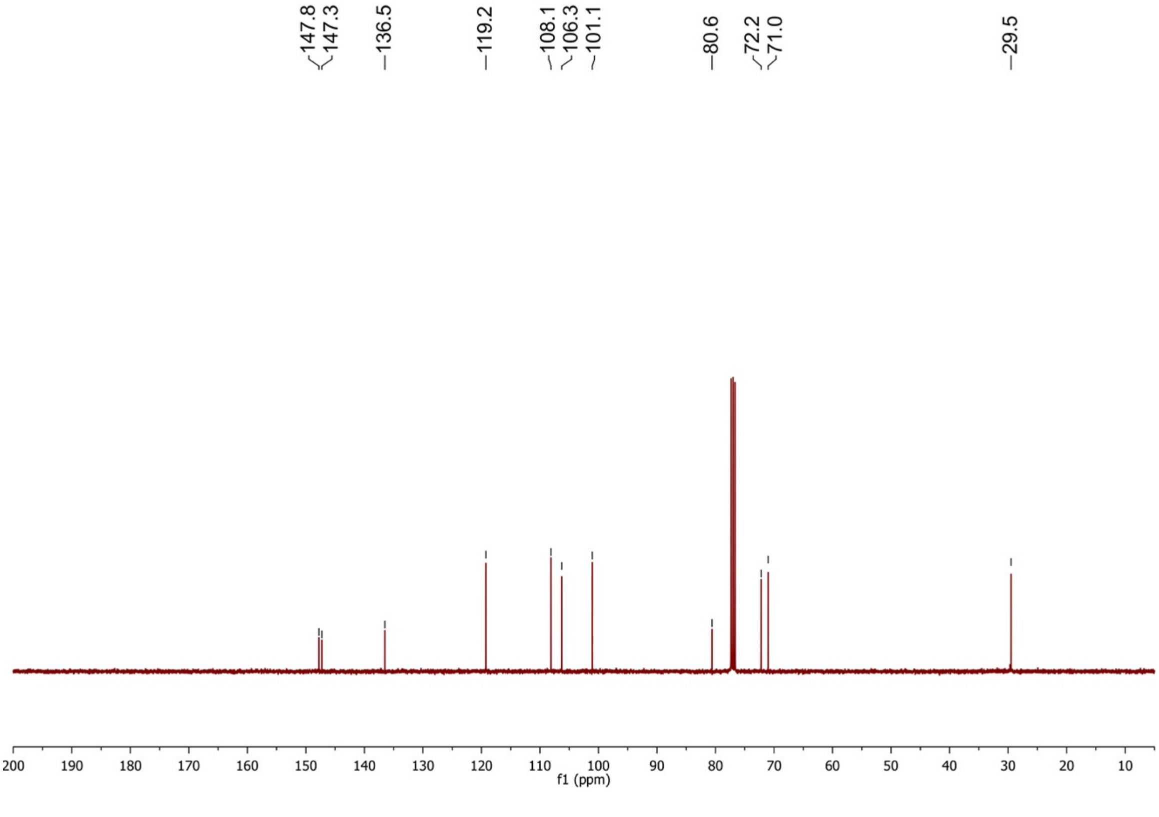

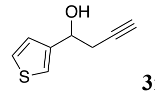

3n ^{1}H NMR (401 MHz, $CDCl_3$)

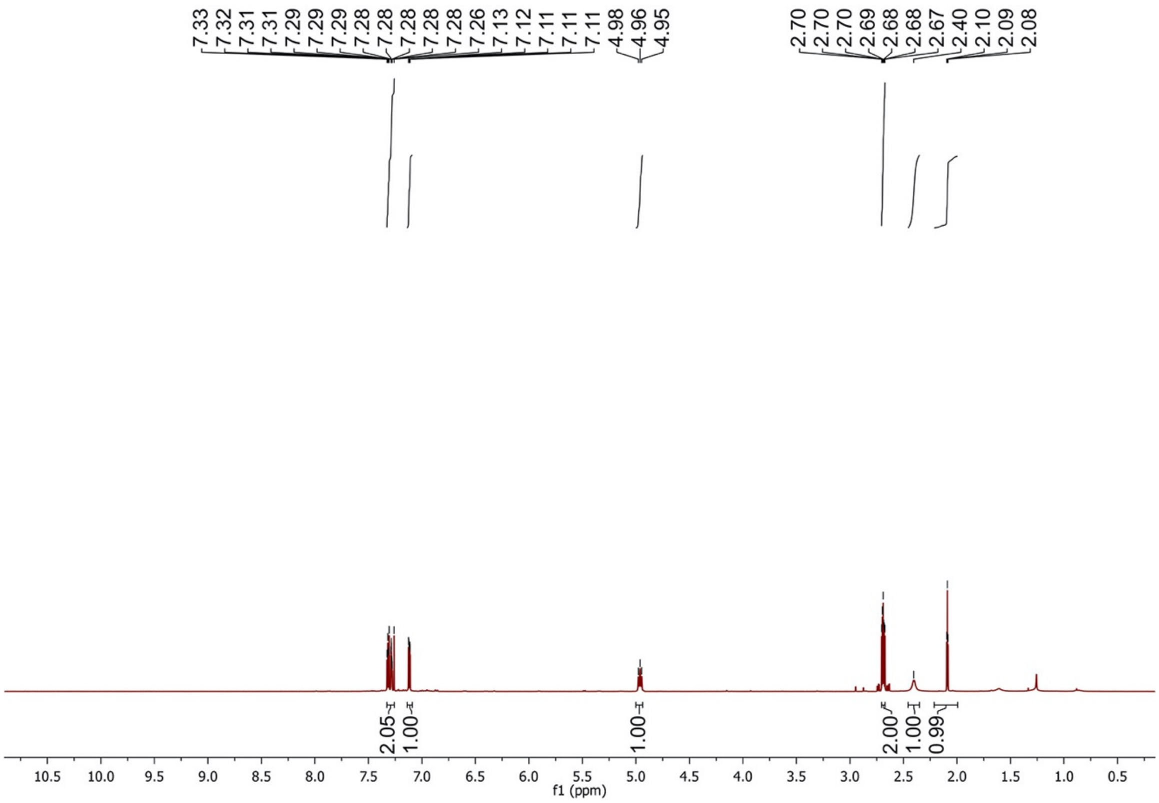

$^{13}C\{^{1}H\}$ NMR (101 MHz, $CDCl_3$)

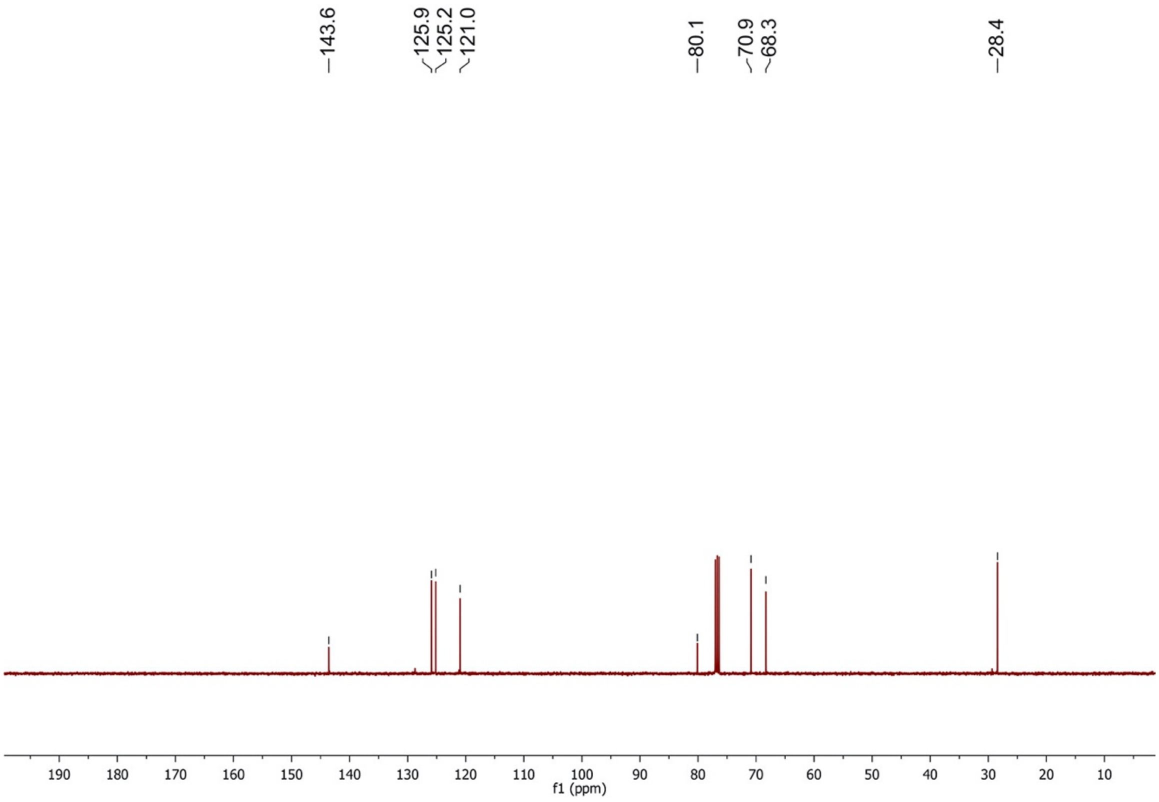

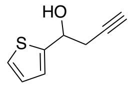

3o ^{1}H NMR (401 MHz, $CDCl_3$)

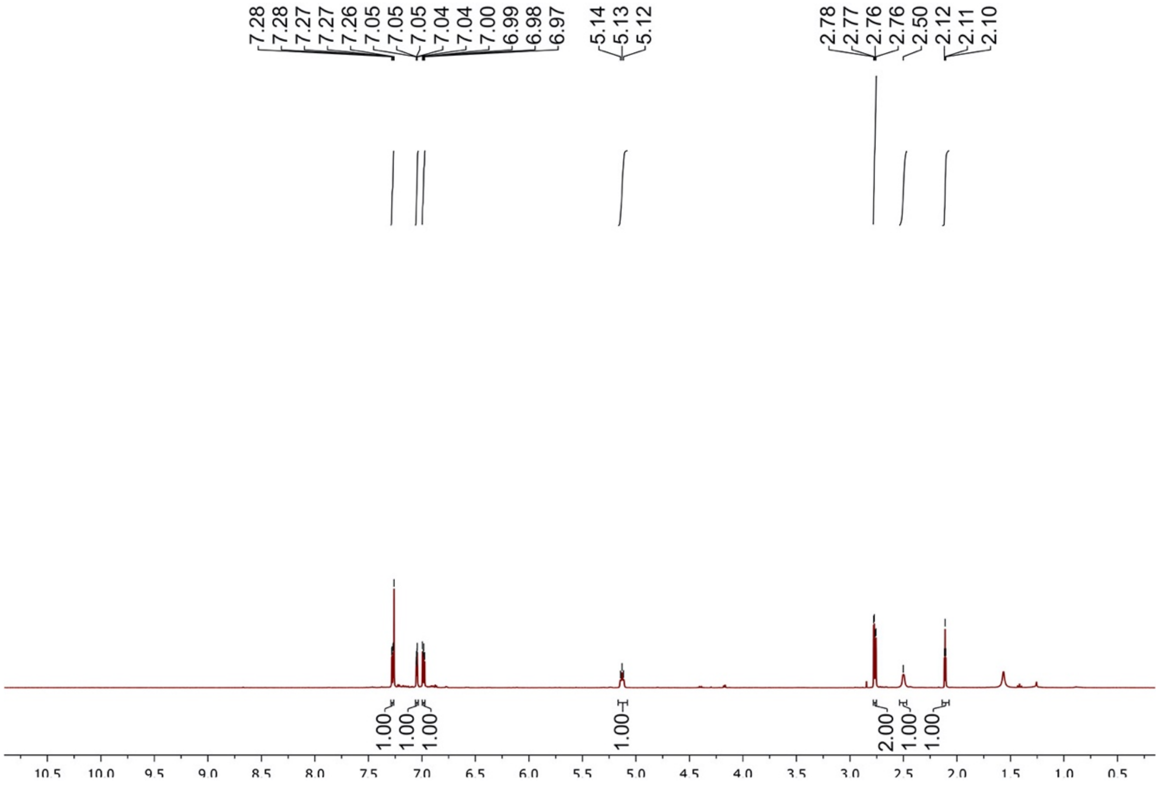

$^{13}C\{^{1}H\}$ NMR (101 MHz, $CDCl_3$)

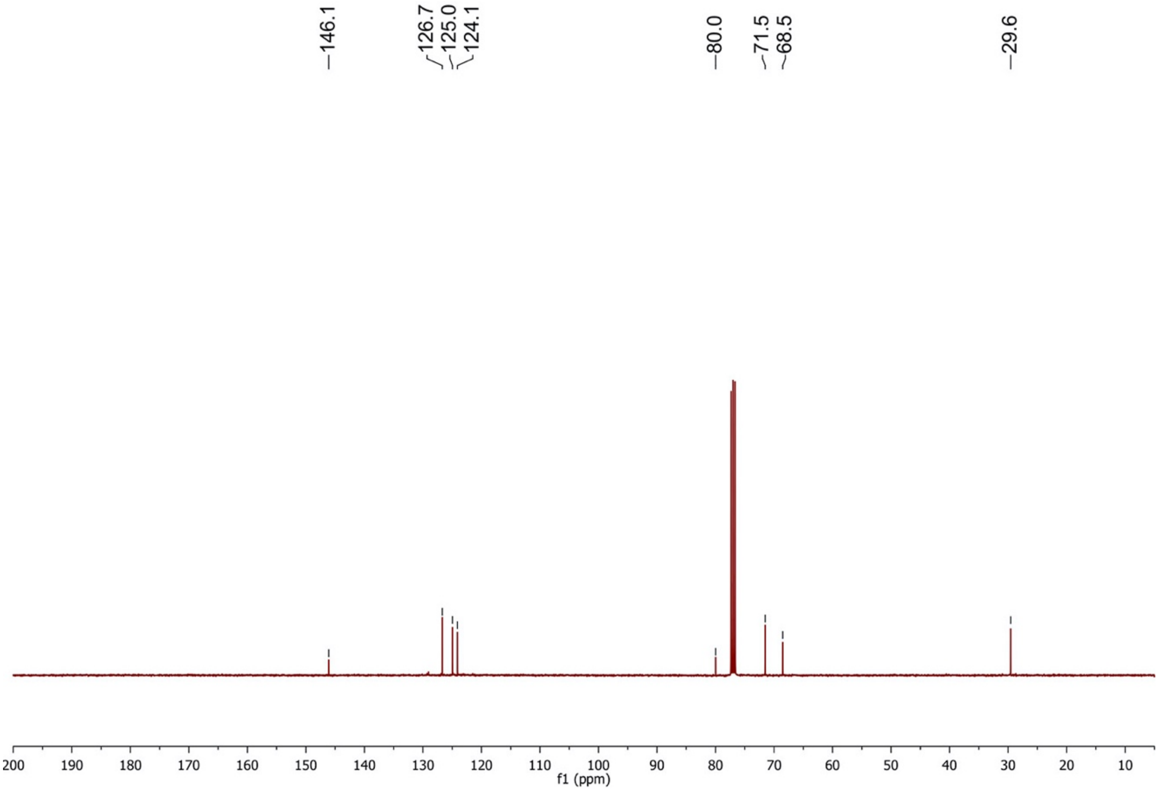

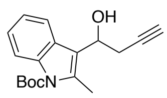

3p ^{1}H NMR (401 MHz, $CDCl_3$)

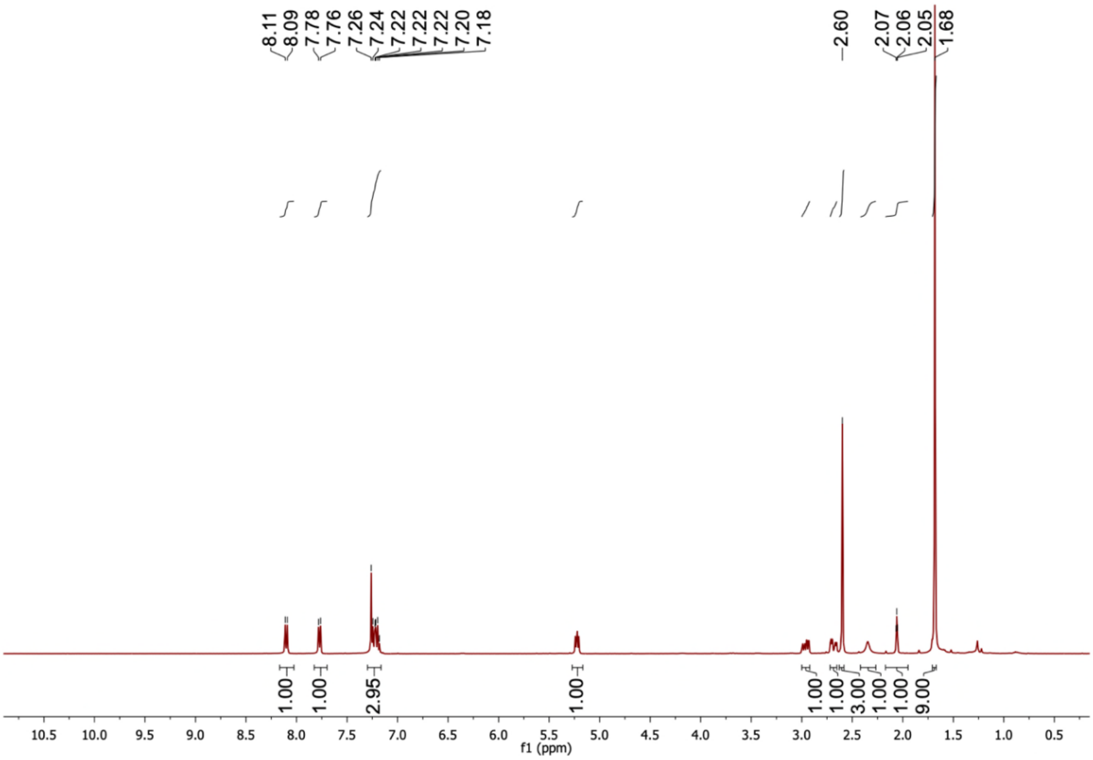

^{13}C{^{1}H} NMR (101 MHz, $CDCl_3$)

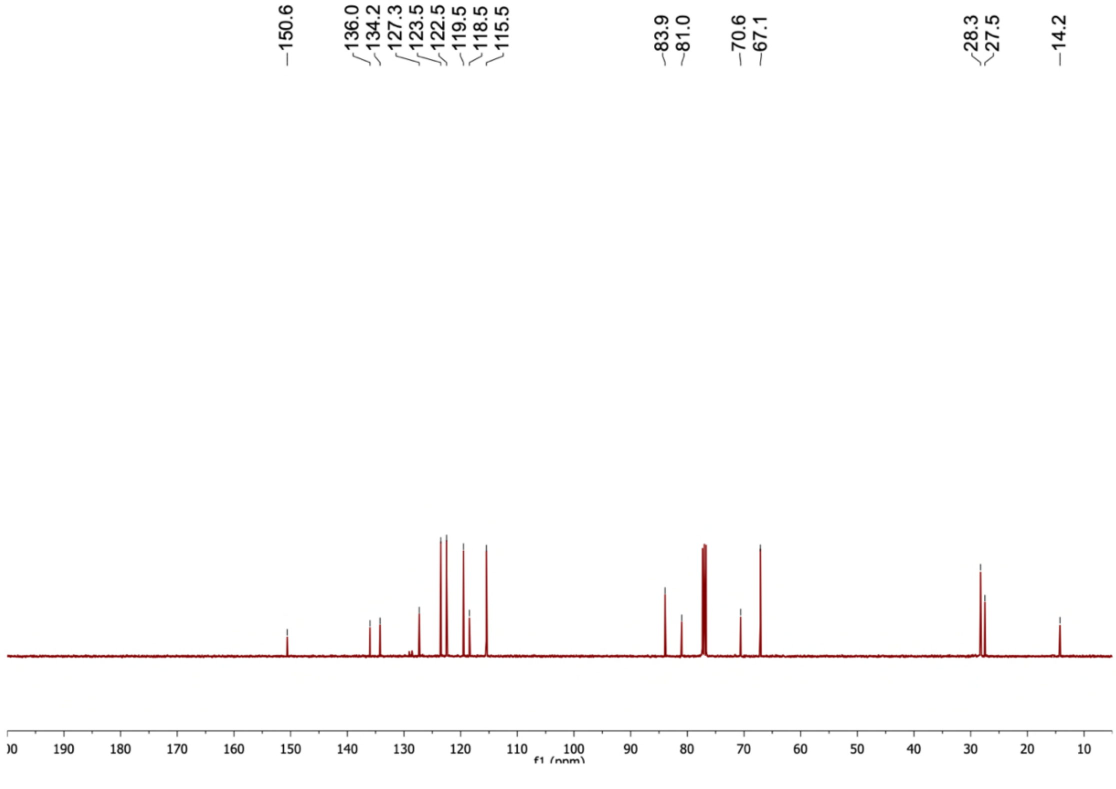

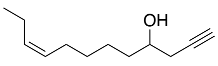

3q ^{1}H NMR (401 MHz, $CDCl_3$)

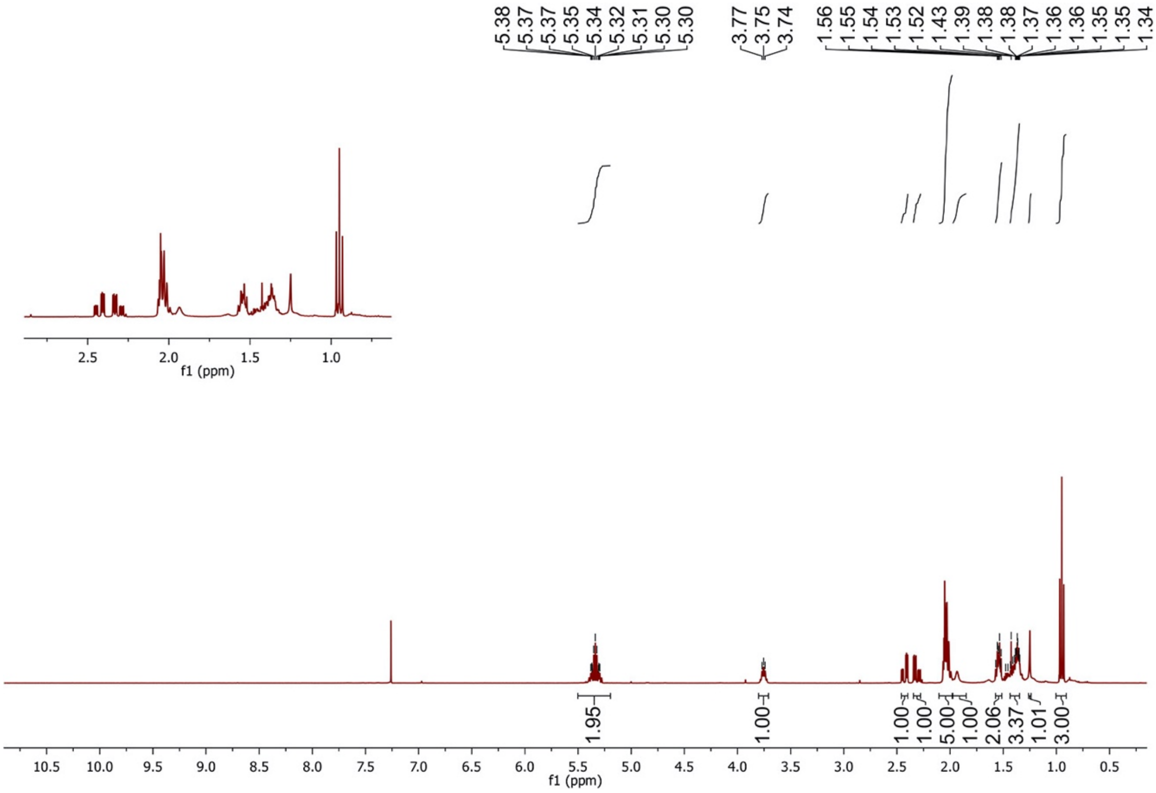

$^{13}C\{^{1}H\}$ NMR (101 MHz, $CDCl_3$)

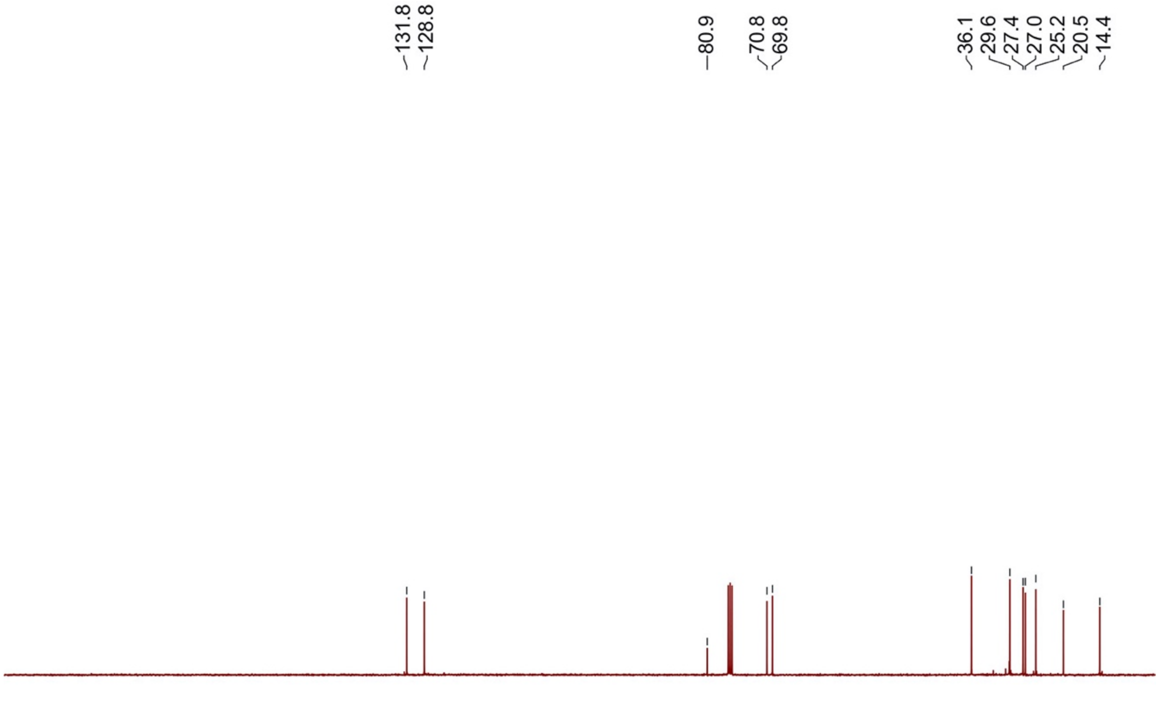

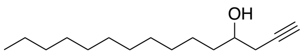

3r ^{1}H NMR (401 MHz, $CDCl_3$)

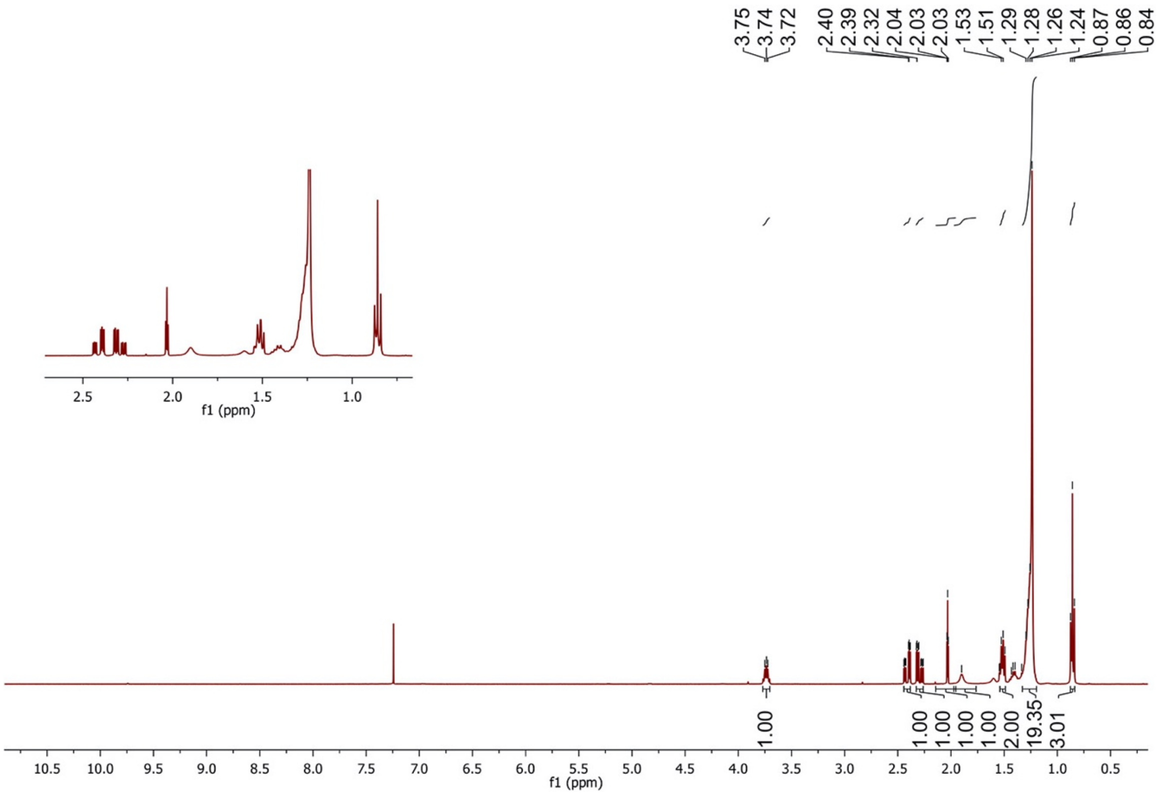

^{13}C{^{1}H} NMR (101 MHz, $CDCl_3$)

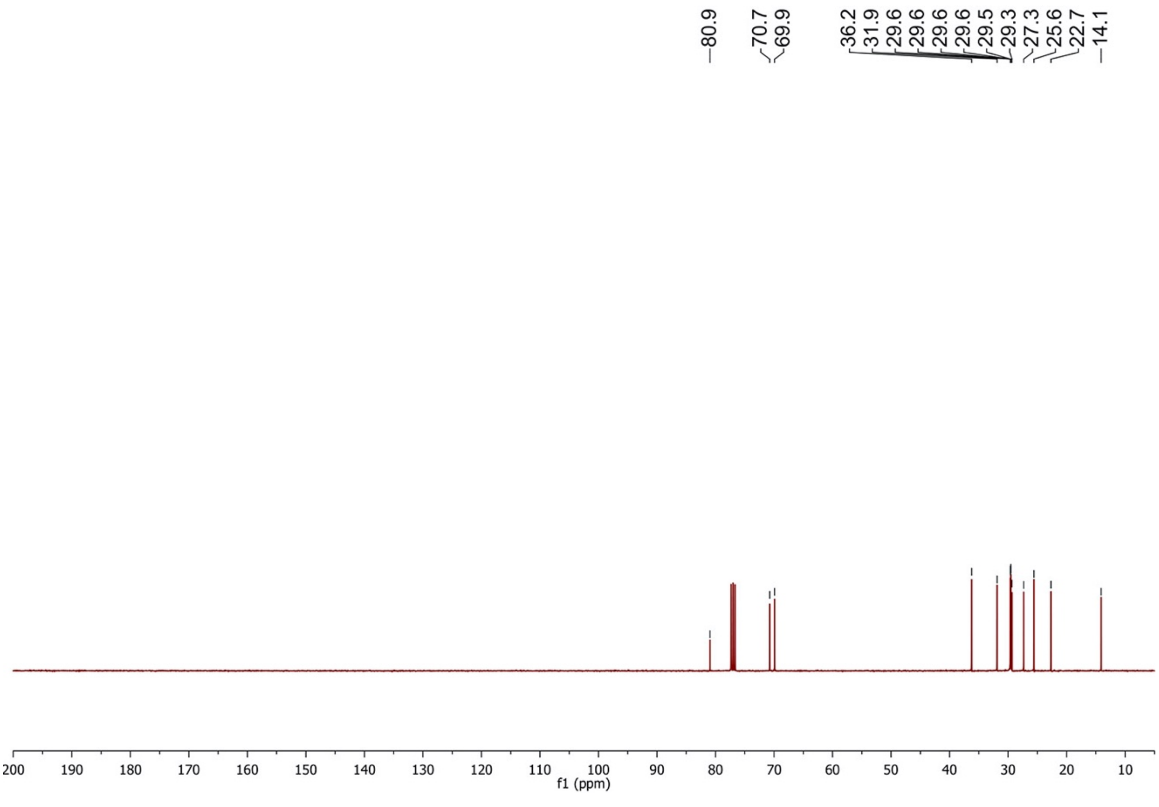

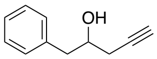

3s ^{1}H NMR (401 MHz, $CDCl_3$)

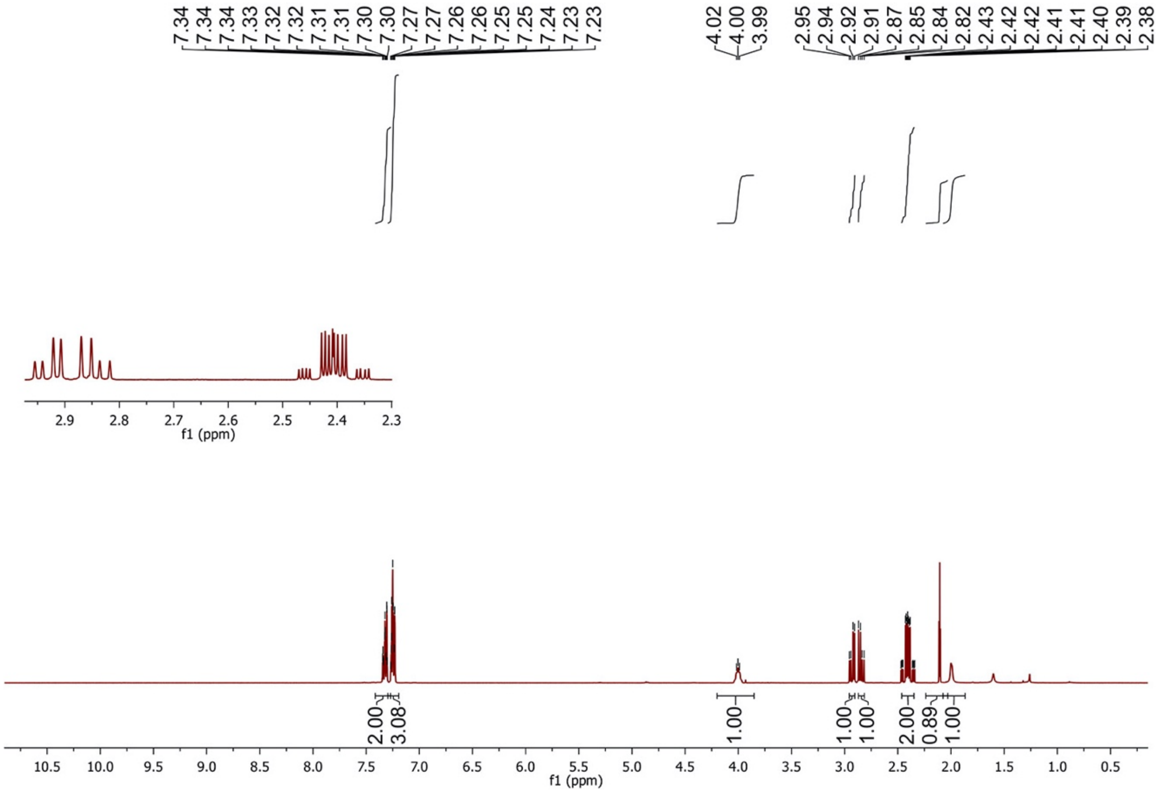

$^{13}C\{^1H\}$ NMR (101 MHz, $CDCl_3$)

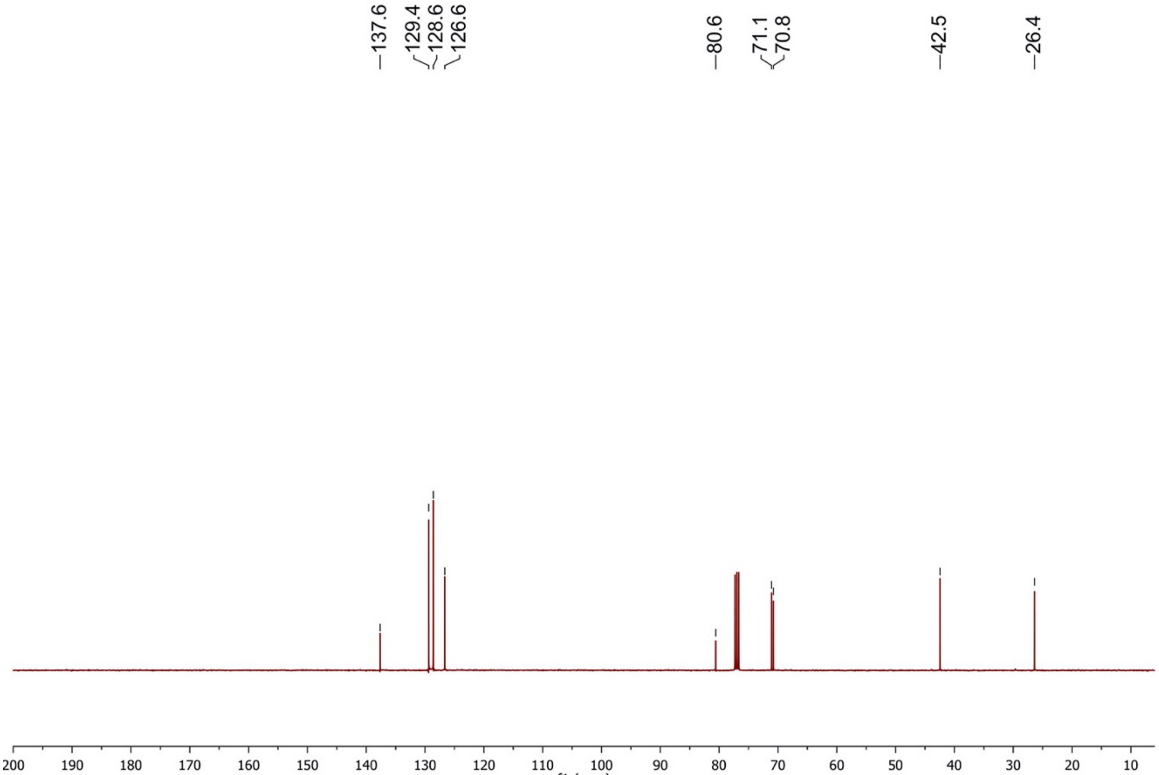

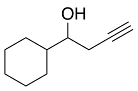

3t ^{1}H NMR (401 MHz, $CDCl_3$)

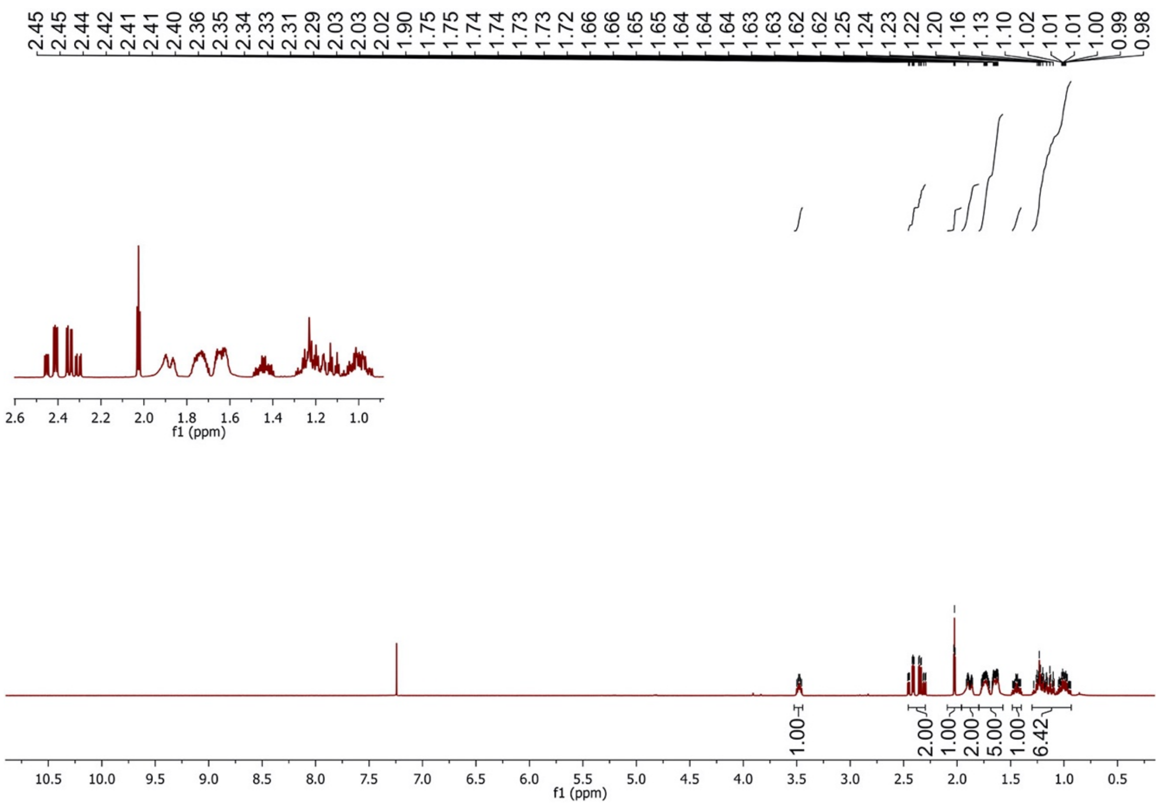

^{13}C{^{1}H} NMR (101 MHz, $CDCl_3$)

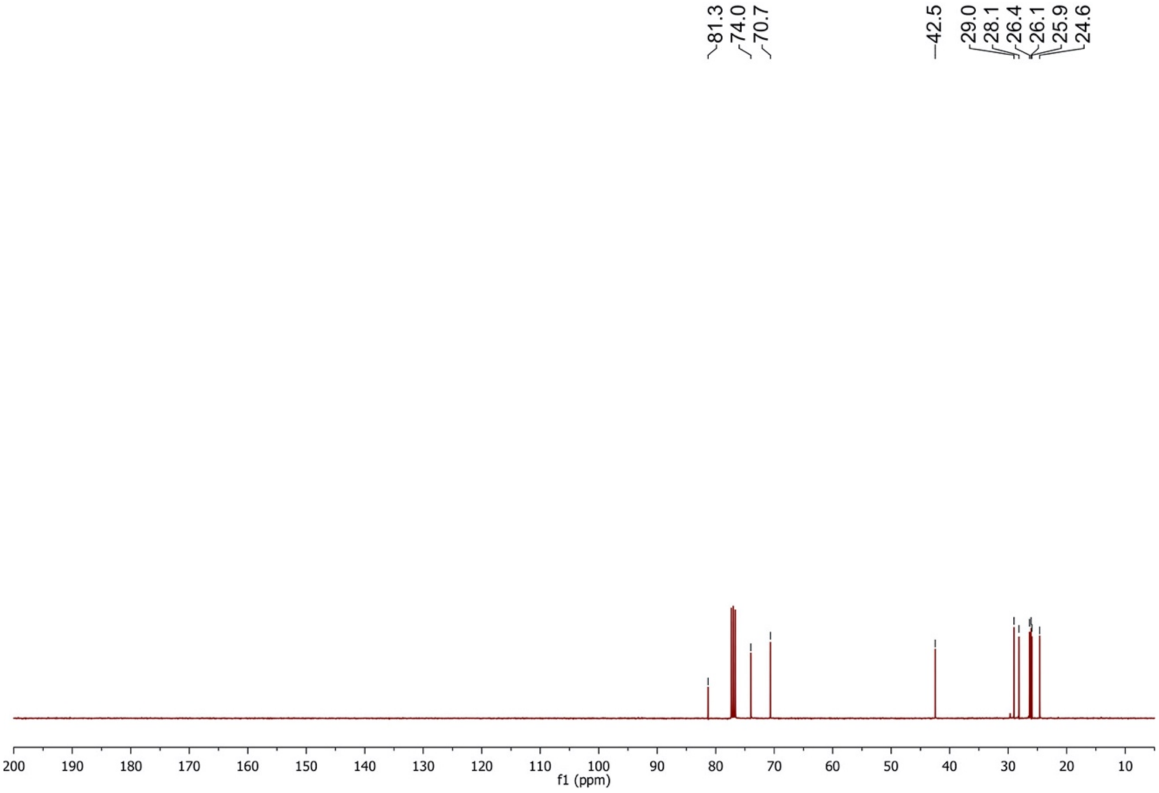

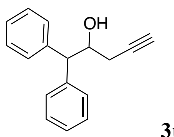

3u ^{1}H NMR (401 MHz, $CDCl_3$)

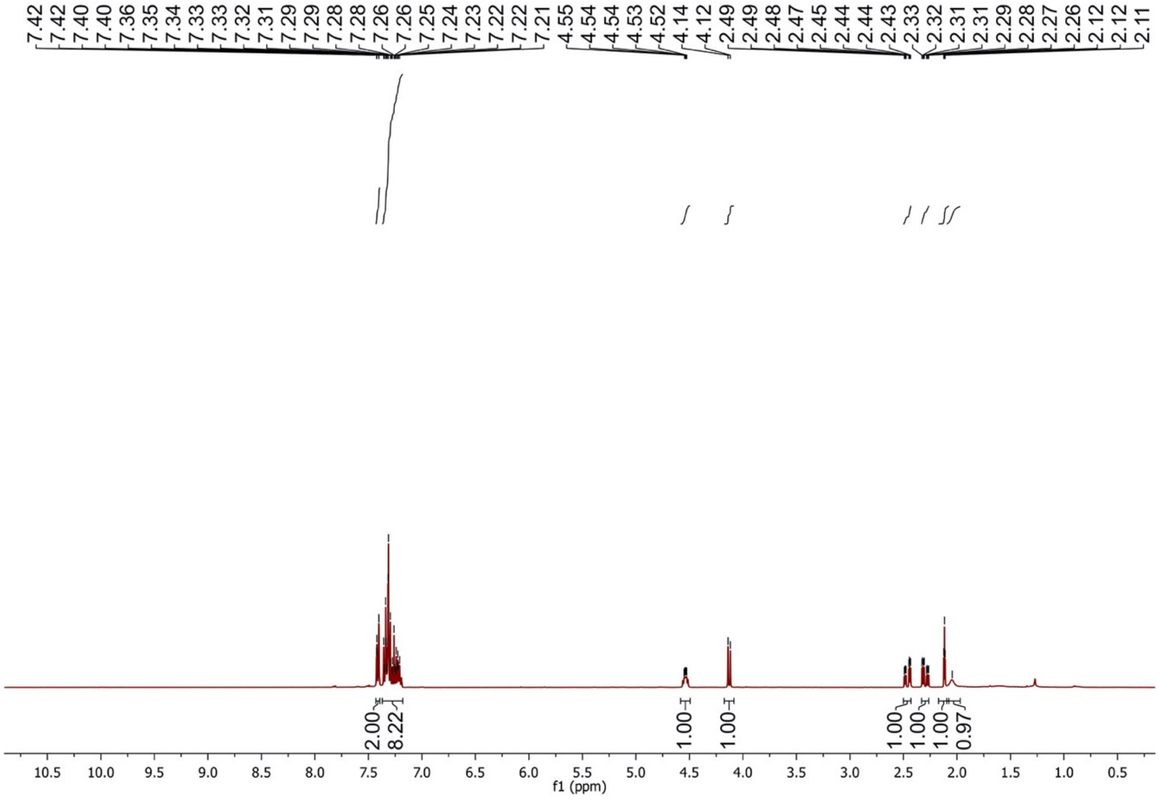

^{13}C{^{1}H} NMR (101 MHz, $CDCl_3$)

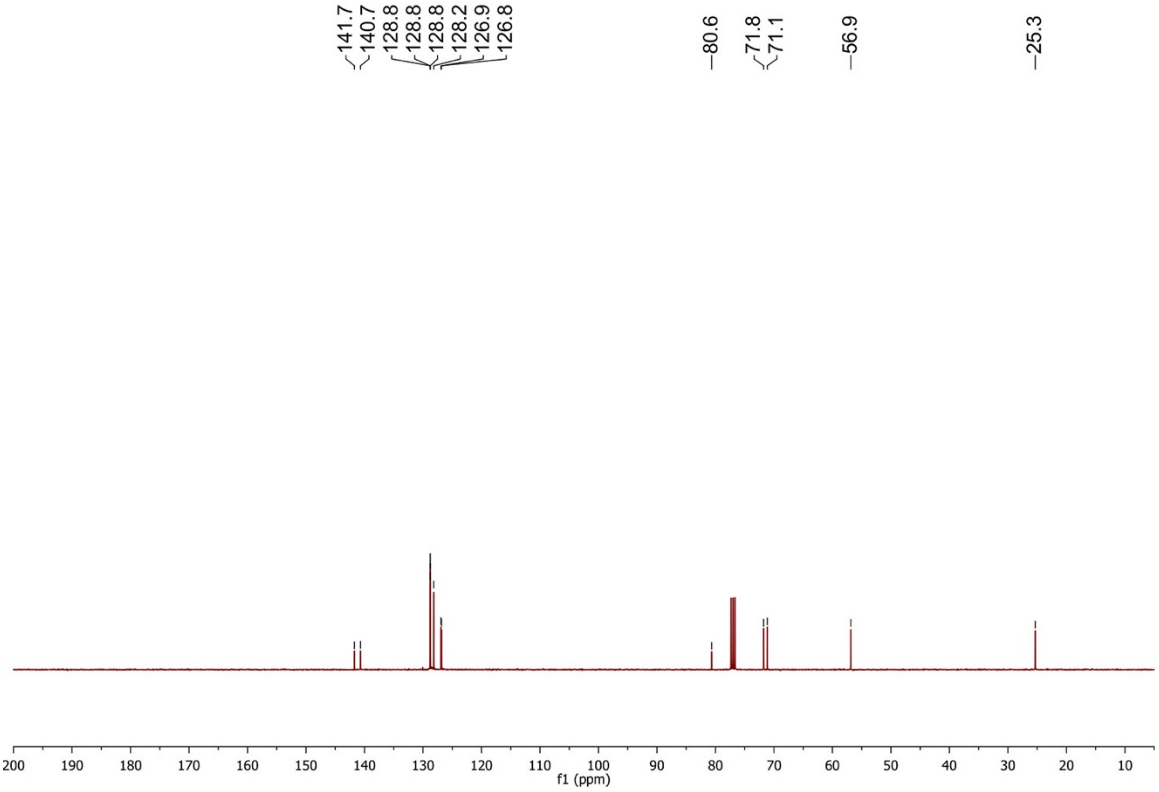

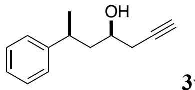

3v ^{1}H NMR (401 MHz, $CDCl_3$)

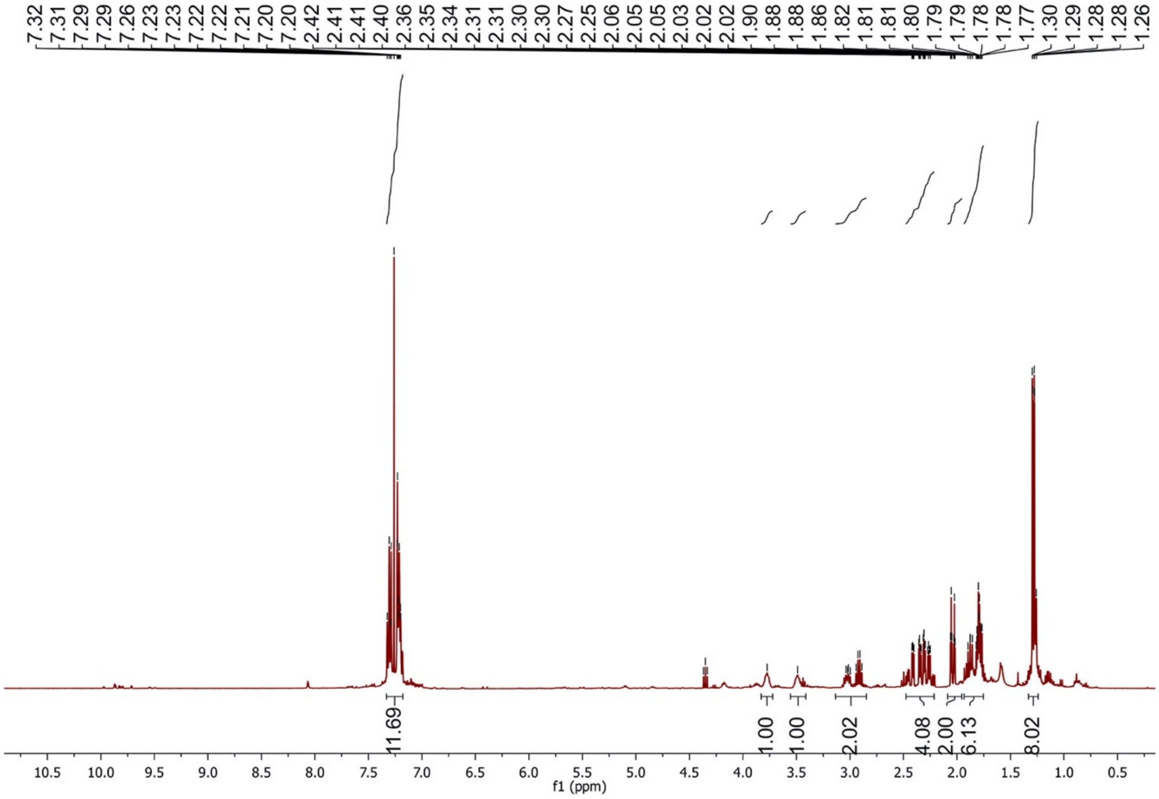

^{13}C{^{1}H} NMR (101 MHz, $CDCl_3$)

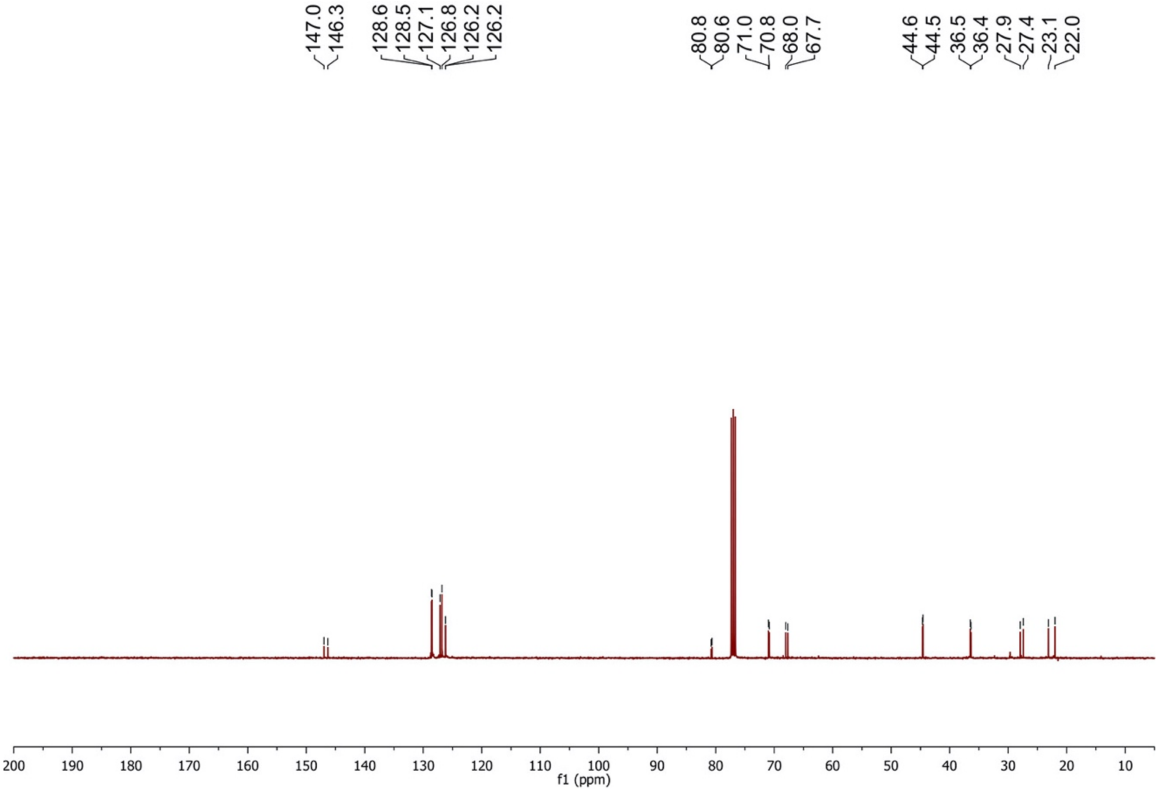

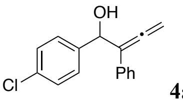

4a ^{1}H NMR (401 MHz, $CDCl_3$)

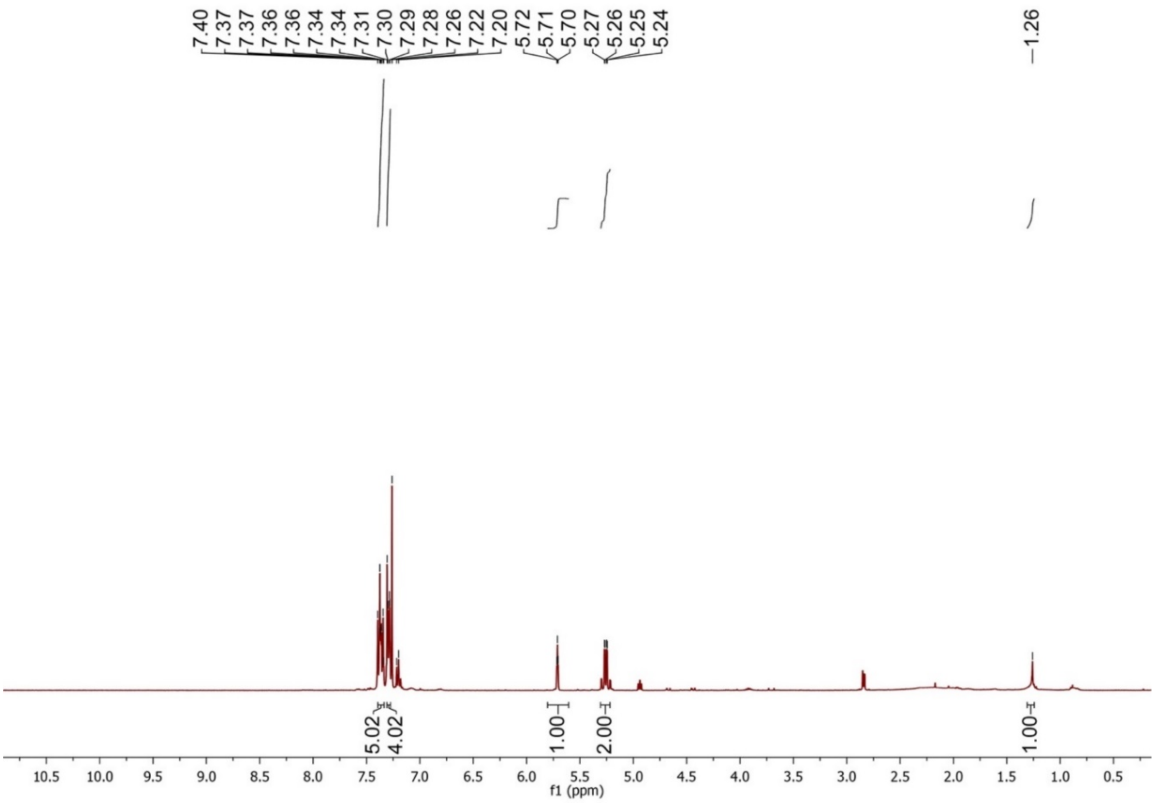

$^{13}C\{^{1}H\}$ NMR (101 MHz, $CDCl_3$)

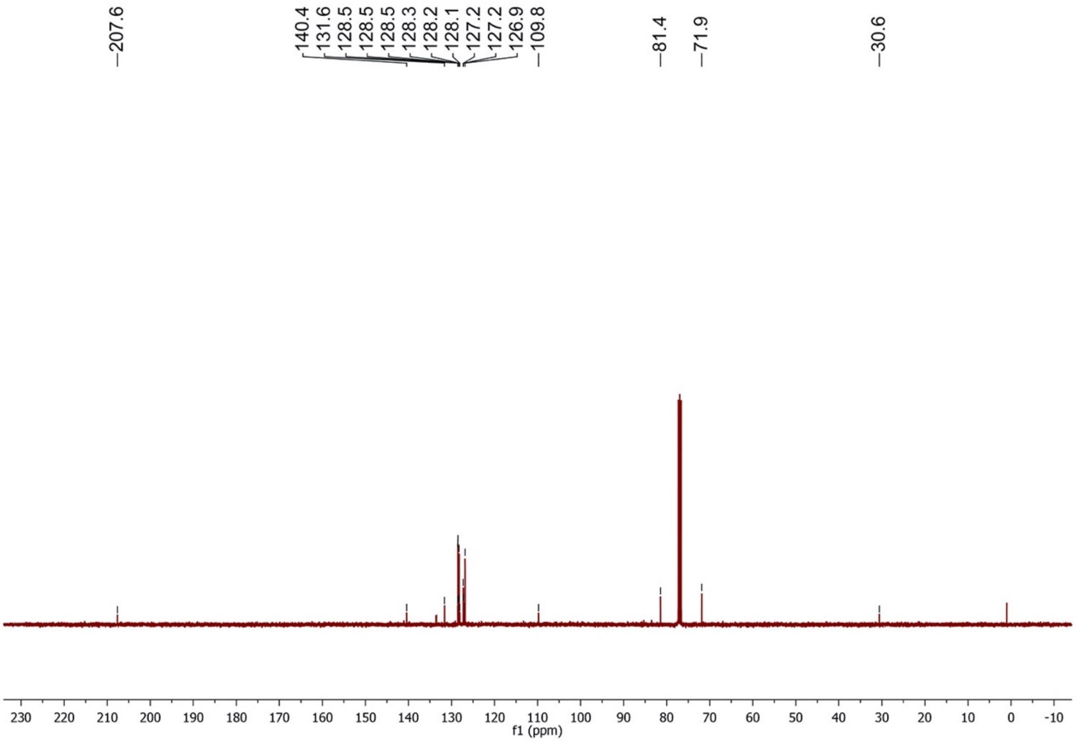

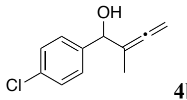

4b ^{1}H NMR (401 MHz, $CDCl_3$)

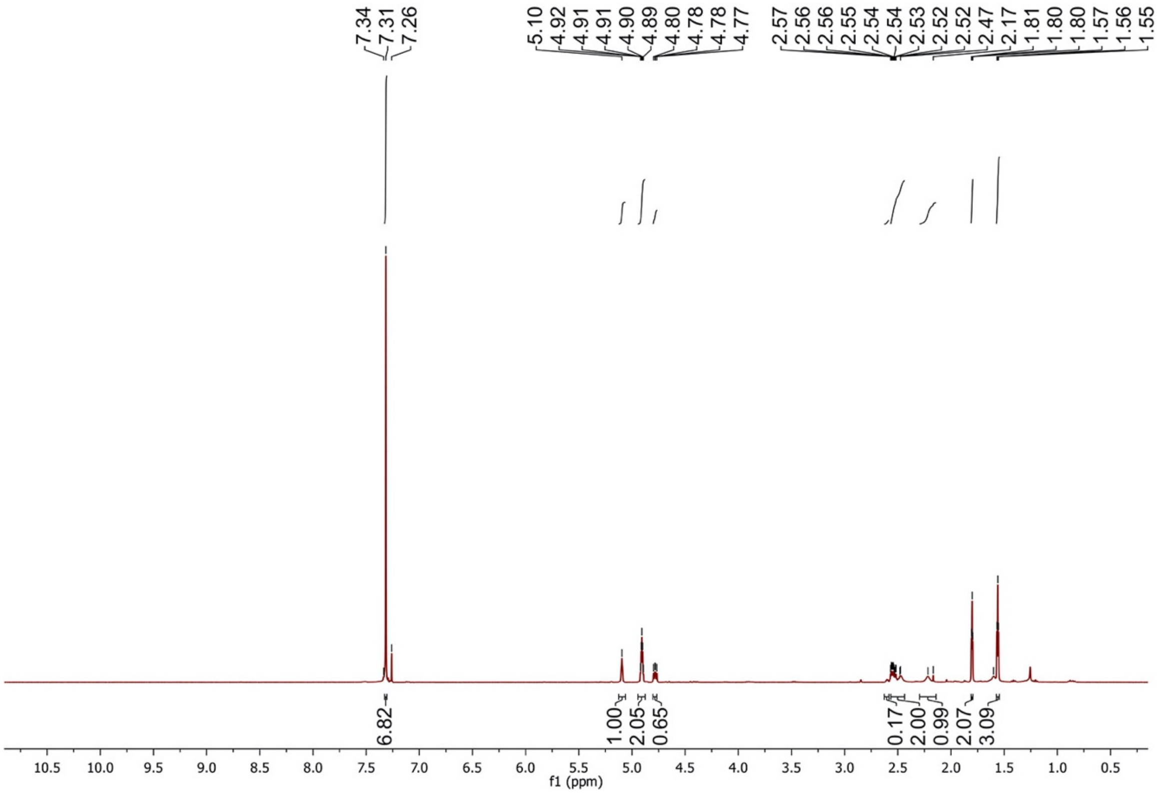

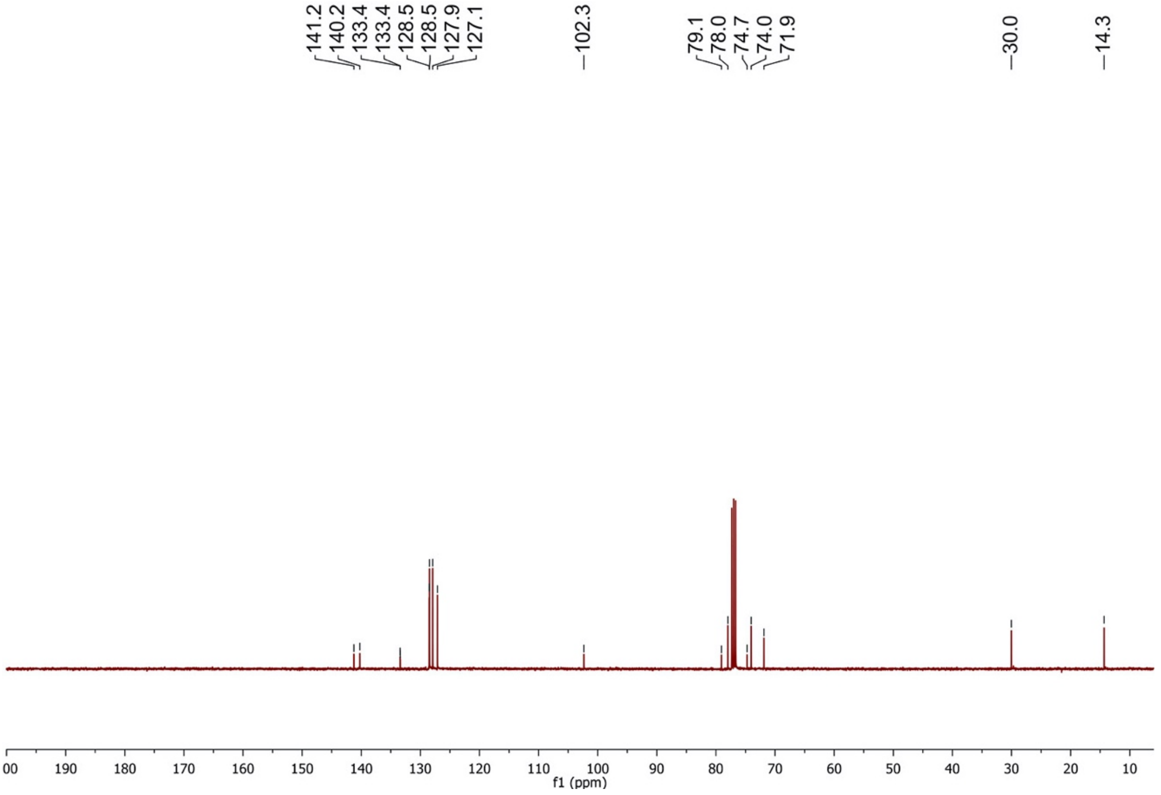

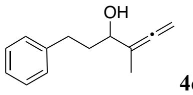

4c ^{1}H NMR (401 MHz, $CDCl_3$)

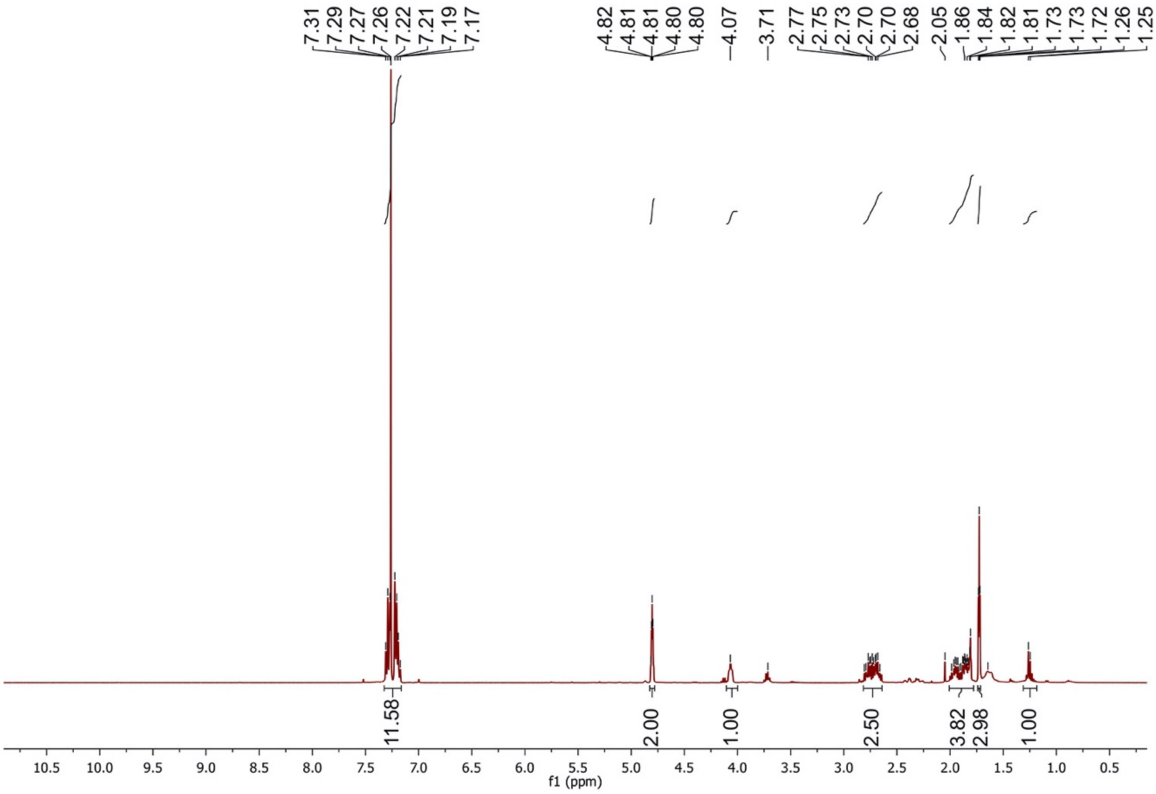

^{13}C{^{1}H} NMR (101 MHz, $CDCl_3$)

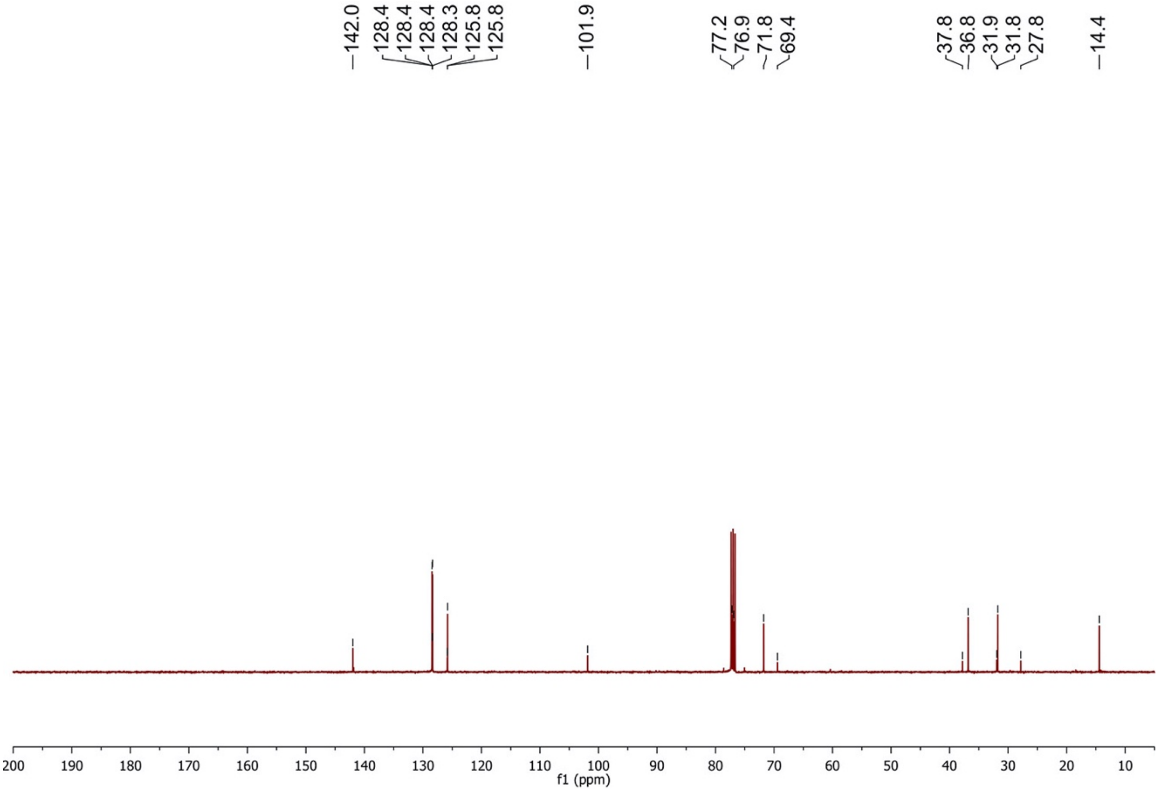

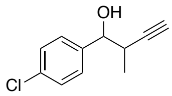

5a ^{1}H NMR (401 MHz, $CDCl_3$)

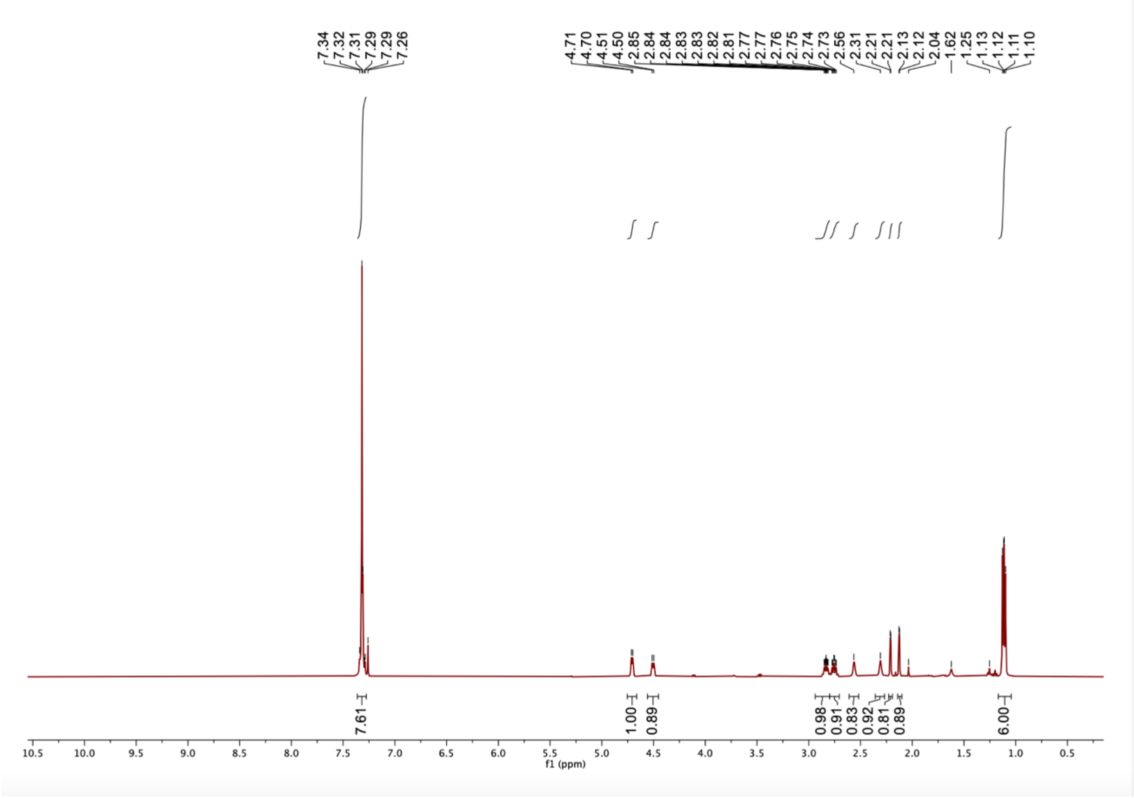

$^{13}C\{^{1}H\}$ NMR (101 MHz, $CDCl_3$)

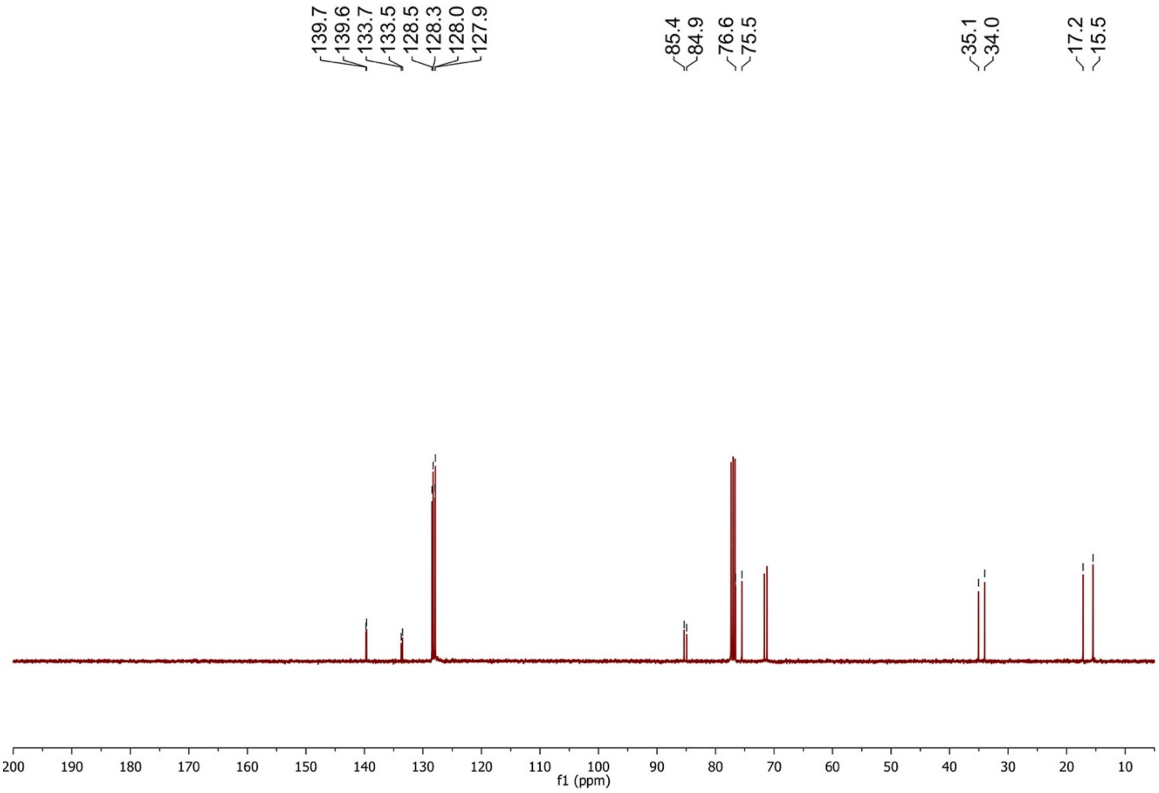

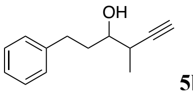

5b ^{1}H NMR (401 MHz, $CDCl_3$)

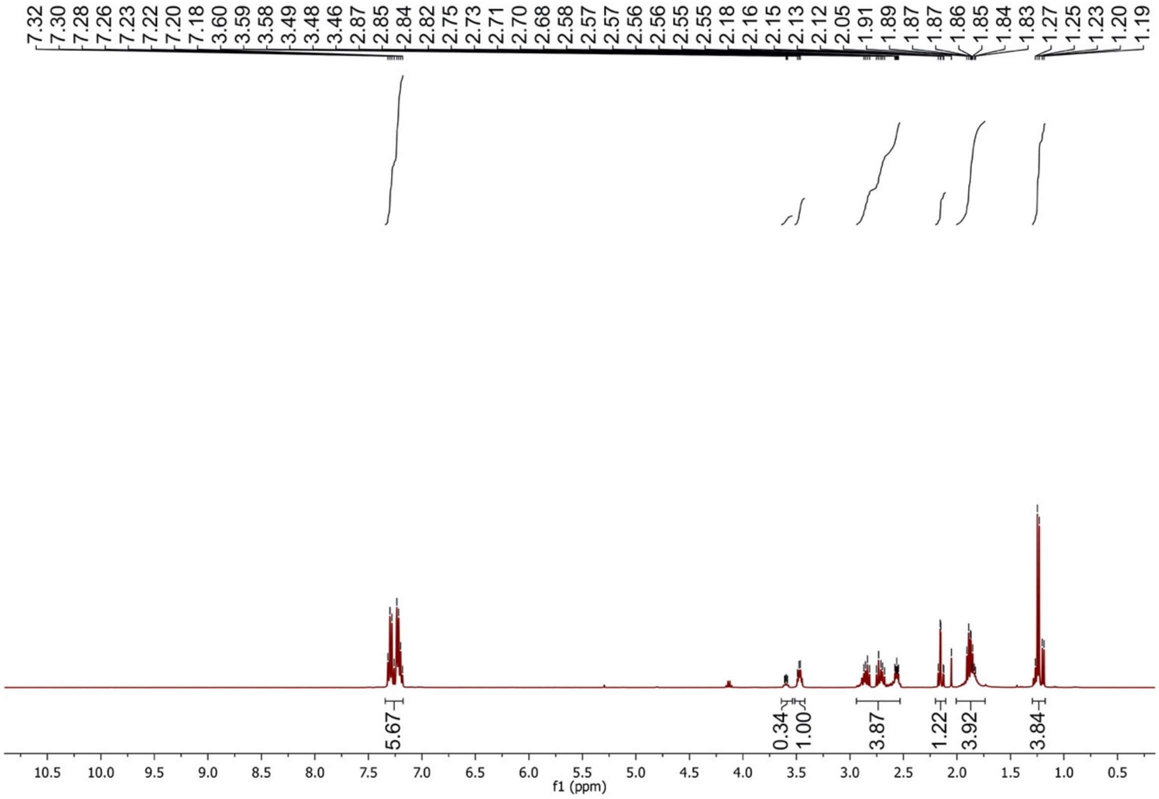

$^{13}C\{^{1}H\}$ NMR (101 MHz, $CDCl_3$)

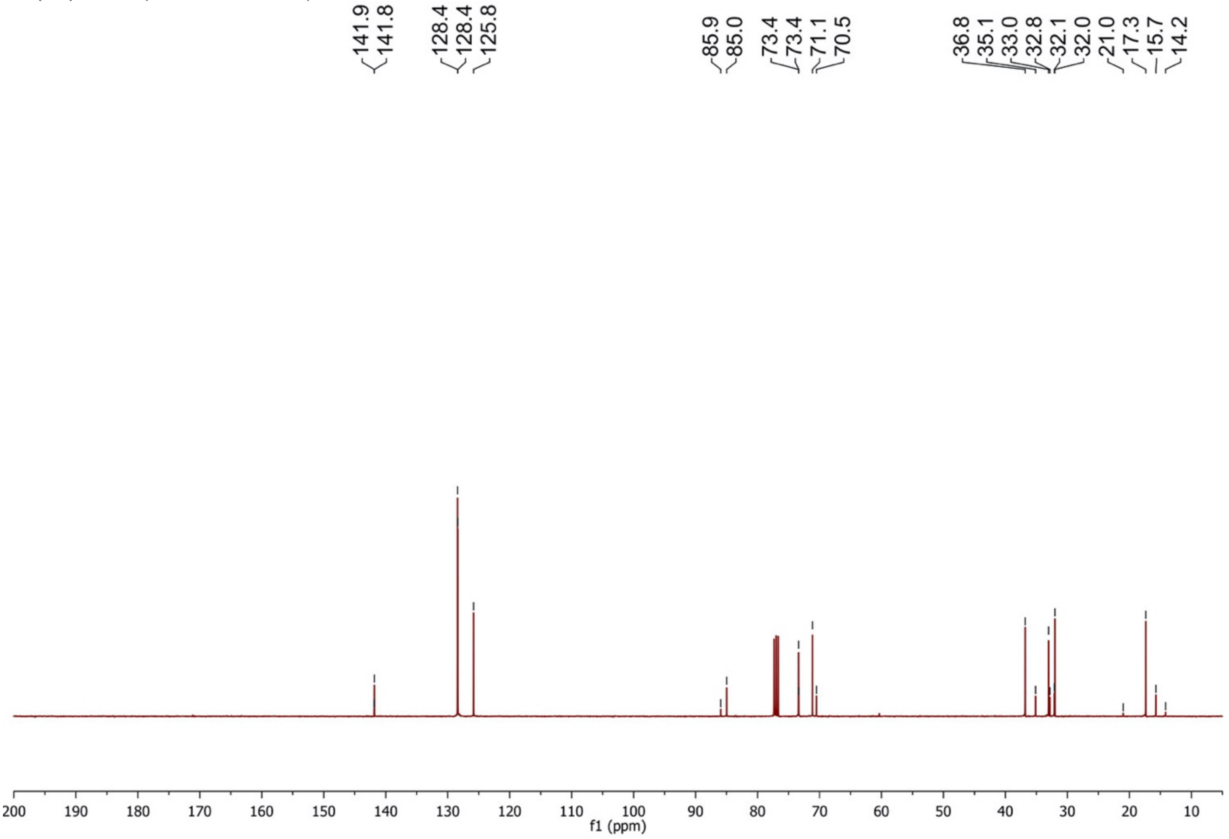

Supplement: Supplementary file 1 — jo1c00521_si_001.pdf [file jo1c00521_si_001.pdf]
